# Asymmetric Organocatalytic Homologation: Access to Diverse Chiral Trifluoromethyl Organoboron Species

**DOI:** 10.1002/chem.202202059

**Published:** 2022-08-18

**Authors:** Ramasamy Jayarajan, Tautvydas Kireilis, Lars Eriksson, Kálmán J. Szabó

**Affiliations:** ^1^ Department of Organic Chemistry Stockholm University SE-106 91 Stockholm Sweden; ^2^ Department of Materials and Environmental Chemistry Stockholm University SE106 91 Stockholm Sweden

**Keywords:** asymmetric homologation, enantioselective, organoboron, organocatalysis, organofluorine

## Abstract

A broad range of aliphatic, aromatic, and heterocyclic boronic acids were successfully homologated using trifluorodiazoethane in the presence of BINOL derivatives to provide the corresponding chiral trifluoromethyl containing boronic acid derivatives in high yields and excellent enantioselectivity. The in situ conversion of the chiral transient boronic acids to the corresponding alcohols or *β*‐CF3 carboxylates are also demonstrated.

## Graphical abstract

Highly enantioselective α‐trifluoromethylated aliphatic, aromatic, and heterocyclic boronic acids and boronates were synthesized from simple feedstocks.

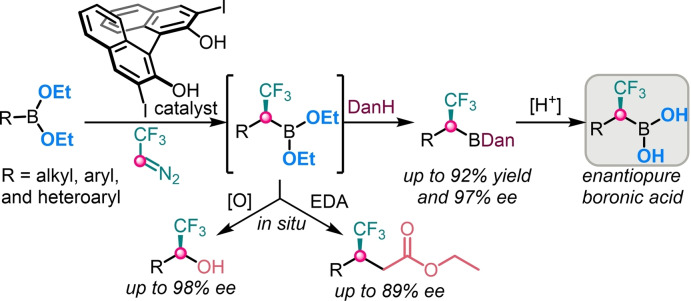

## Introduction

**Figure 1:**
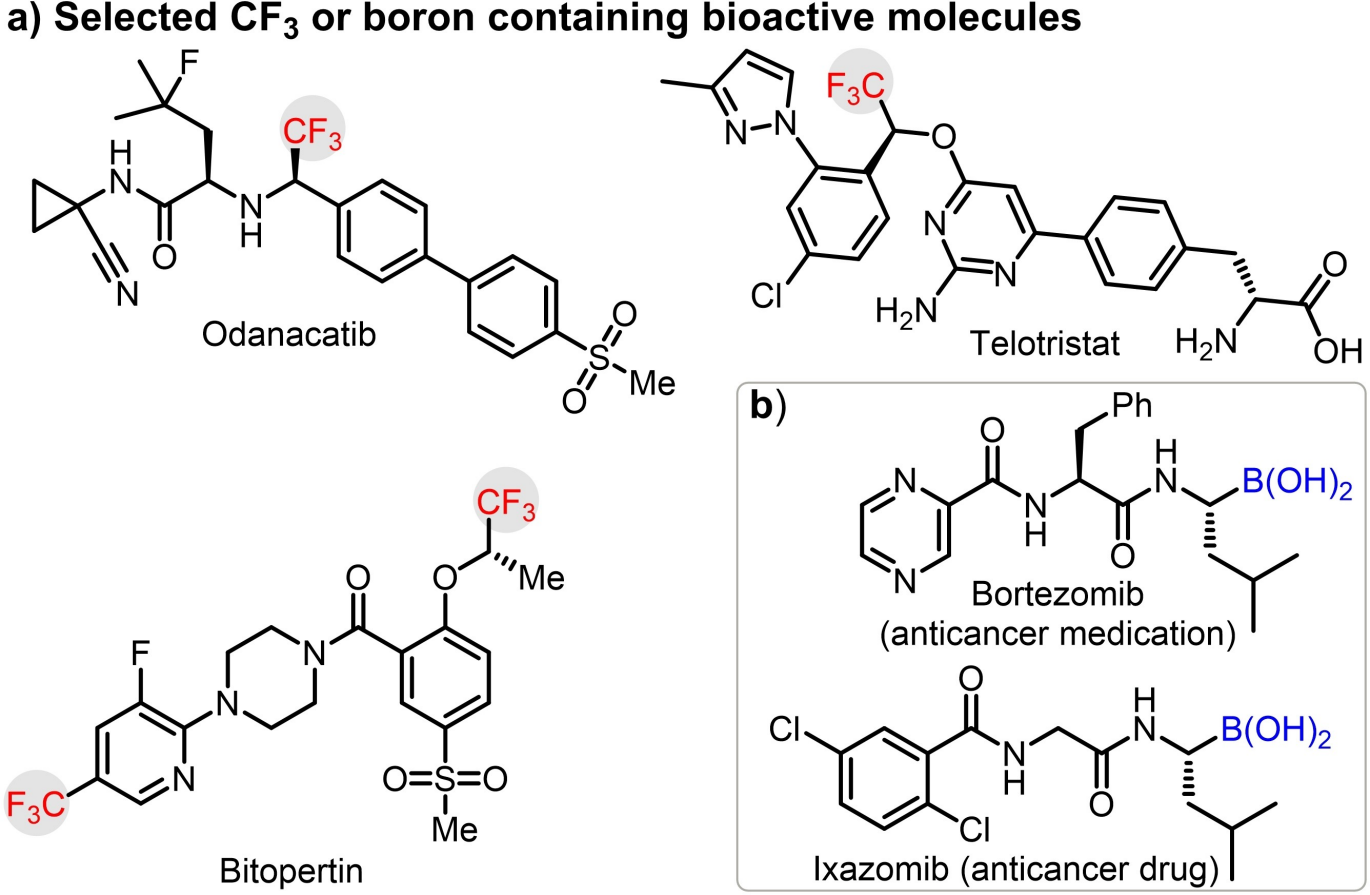
Bioactive trifluoromethylated and boronic acid derivatives.

Developing catalytic methods to incorporate fluorinated subunits into organoboron compounds is a key interest in the contemporary research area owing to its potential pharmaceutical, agrochemical, and material applications.^[1]^ The chiral CF_3_ motif is a privileged substructure found in various drug candidates, such as Odanacatib,^[2]^ Telotristat,^[3]^ and Bitopertin^[4]^ (Figure 1a). Further, the boron bound carbon stereocenters is an integral part of clinically approved anticancer drugs^[5]^ such as Bortezomib^[6]^ and Ixazomib^[7]^ (Figure 1b). Despite the synthetic and biological importance of trifluoromethylated organoboranes, their asymmetric preparations have remained a challenge. Classical hydroboration^[8]^ is a possible method to generate α‐CF_3_ organoboranes. However, isolation of products obtained by hydroboration is difficult due to stability problems.

**Scheme 1:**
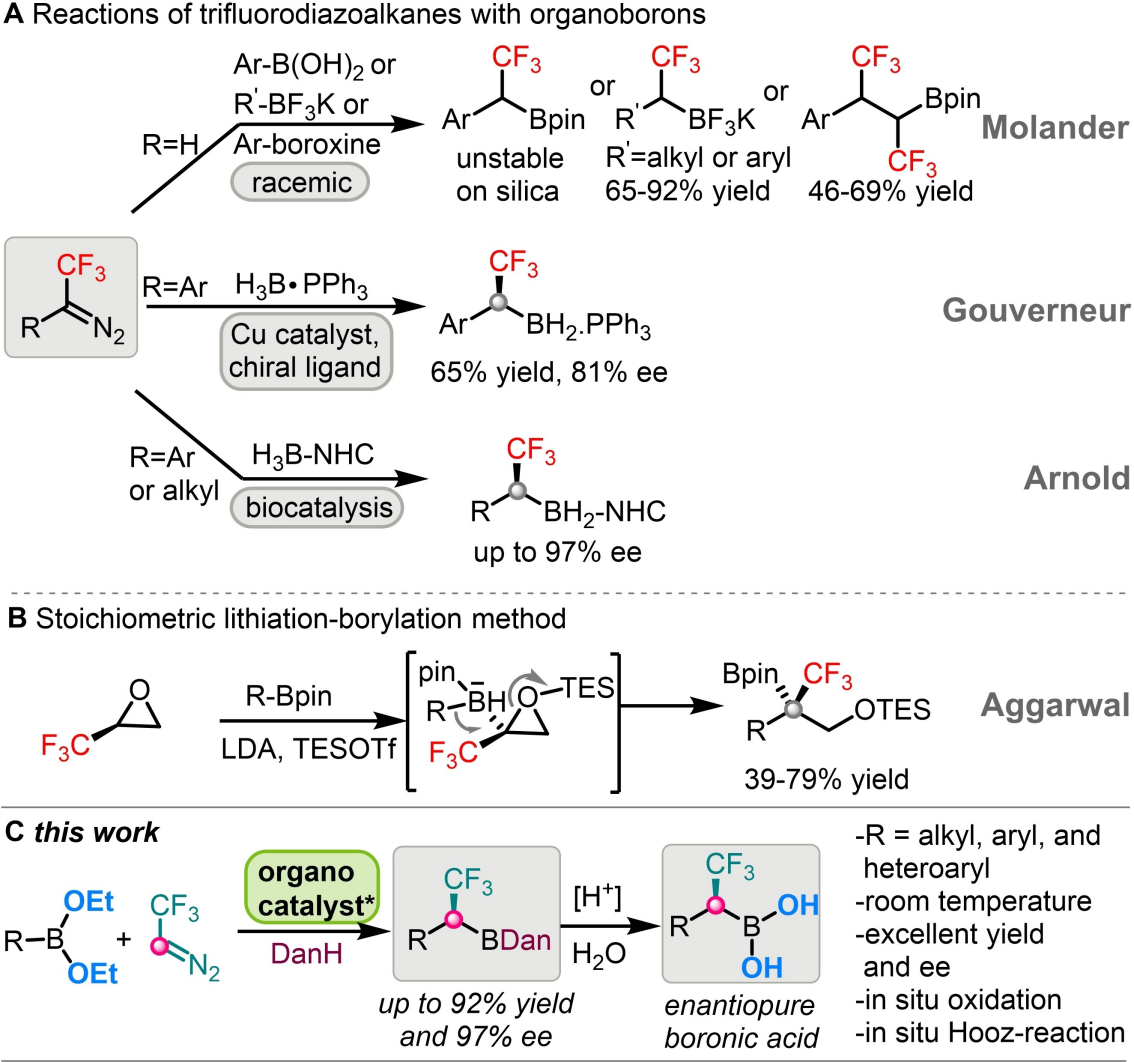
Methods to access enantioenriched α‐CF_3_ organoborons

Trifluorodiazoethane has been proven to be a valuable reagent for the rapid synthesis of various classes of CF_3_‐substituted organic molecules.^[9]^ Molander and co‐workers^[10]^ reported a metal‐free (racemic) homologation method to prepare α‐trifluoromethylated alkyl or aryl boron compounds using CF_3_CHN_2_ as a nucleophilic partner. The α‐trifluoromethylated products were isolated as trifluoroborates since the pinacol boronates were prone to oxidation upon purification on silica gel chromatography.^[10]^ Further, their subsequent development demonstrated that the mono or bis trifluoromethylation could be modulated by the fine‐tuning of substitution at boron center (Scheme 1A).^[11]^ Wang,^[12]^ Valdes^[13]^ and Ley^[14]^ also reported useful applications based on homologation of organoboron species with diazo compounds. Gouverneur and co‐workers reported a nice example for copper‐catalyzed enantioselective insertion of CF_3_‐carbene intermediate into the B−H bond.^[15]^ However, this method is limited to aryl‐substituted trifluorodiazo compounds and the only asymmetric α‐CF_3_ boron derivative was isolated with moderate enantioselectivity (81 % ee) (Scheme 1A).

A biocatalytic insertion into B−H bonds of H_3_B‐NHC with trifluorodiazoalkanes was reported by Arnold and co‐workers for the synthesis of chiral α‐trifluoromethylated alkyl‐ and benzylboron‐NHC derivatives with excellent enantioselectivities (up to 97 % ee) (Scheme 1A).^[16]^ In addition, Aggarwal and co‐workers^[17]^ reported a ring‐opening lithiation–borylation route to tertiary α‐trifluoromethylated boronates (Scheme 1B). In general, transformations based on 1,2‐borotropic migration proved to be a very successful strategy for synthesis of chiral organoboron species.[^15^, ^16^, ^17^, ^18^]

We recently reported a new organocatalytic method for the synthesis of chiral α‐CF_3_ allyl boronic acids from alkenyl boroxines by asymmetric homologation strategy.^[19]^ Motivated by this approach and to expand the versatility of the strategy, herein we demonstrate a route to generate a divergent set of chiral α‐CF_3_ containing organoboron reagents from commercially available alkyl, aryl, and heterocyclic boronic acids (Scheme 1C).

## Results and Discussion

The optimal conditions for the asymmetric homologation involved using diethyl phenylboronate **1 a** with 3 equiv. of **2**, and 30 mol % catalyst **3** (Table 1, entry 1). The homologated unstable chiral α‐CF_3_ benzylboronic ester **4 a** was protected with diaminonaphthalene^[20]^ (DanH) to give **5 a** in 87 % yield with 94 % ee. Practically unchanged yield (85 %) and enantioselectivity (94 %) were obtained with 20 mol % catalyst **3** (entry 2) and a slightly lowered selectivity (91 %) with 72 % yield was found with 10 mol % **3** (entry 3). Replacement of iodo‐BINOL **3** with bromo‐BINOL (entry 4) led to decreased yield (77 %) and similar enantioselectivity (93 % ee). Use of unsubstituted BINOL gave 76 % yield and relatively low selectivity (79 % ee) with opposite the enantiomer (entry 5). The absence of catalyst (entry 6) led to a slow racemic reaction (yield 6 %), indicating a low reactivity of diethyl phenylboronate **1 a** precursor toward **2**. When phenyl‐Bpin was used as the substrate in the presence of **3** (entry 7), no reaction was observed. Replacement of diethyl phenylboronate **1 a** with diisopropyl phenylboronate (entry 8) led to a substantial decrease of the yield (22 %) but with still a good selectivity (91 % ee). A complex reaction mixture was obtained, when the reaction was performed with either PhB(OH)_2_ (as received from a commercial source) or boroxine, (prepared by Dean‐Stark apparatus) (entries 9–10). When boroxine was used with an additive 2 equiv. of EtOH (entry 11) the reaction provided 28 % yield and relatively low selectivity (82 % ee). This was a major difference from the asymmetric homologation of vinyl boroxines with **2**, which proceeded with high yield and selectivity.^[19]^ Without molecular sieves, the reaction still proceeded smoothly with excellent yield and ee (entry 12). However, the molecular sieves were used in all the reactions to absorb any trace of moisture for standardization of the reaction conditions. Changing DCM to toluene (entry 13) leads to slightly lowering the yield and the ee.

**Table 1:** Optimal reaction condition for asymmetric homologation.^[a]^

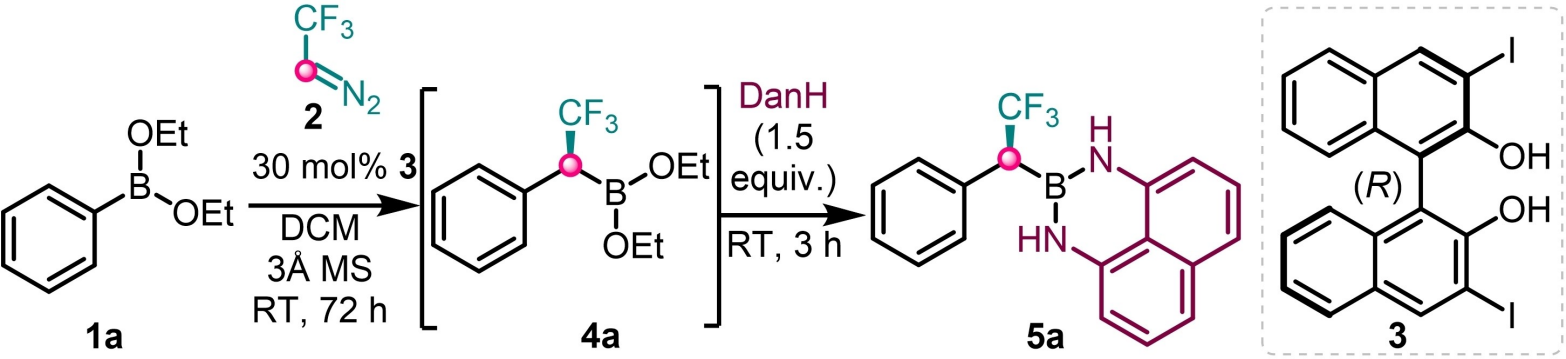			
Entry	Change from standard condition	Yield [%]^[b]^	ee [%]
1	No change	87	94
2	20 mol % of catalyst **3**	85	94
3	10 mol % of catalyst **3**	72	91
4	(R)‐bromo‐BINOL instead of **3**	77	93
5	(R)‐(+)‐1,1′‐Bi(2‐naphthol) instead of **3**	76	79
6	No catalyst **3** is used	6 (13)	nd
7	PhBpin instead of **1 a**	nd	nd
8	PhB(^*i*^PrO)_2_ instead of **1 a**	22 (43)	91
9	PhB(OH)_2_ instead of **1 a**	(18)	nd
10	Boroxine instead of **1 a**	(5)	nd
11	Boroxine instead of **1 a**+2 equiv. EtOH	28	82
12	No molecular sieves (MS) added	83	94
13	Toluene as a solvent	85	90

[a] Unless otherwise stated: **1 a** (0.1 mmol), **2** (0.3 mmol) and **3** (0.03 mmol, 30 mol %) in 1.0 mL of DCM stirred at room temperature for 72 h, and then DanH (0.15 mmol) was added. [b] Isolated yields and the NMR yields are given in parentheses.

**Scheme 2:**
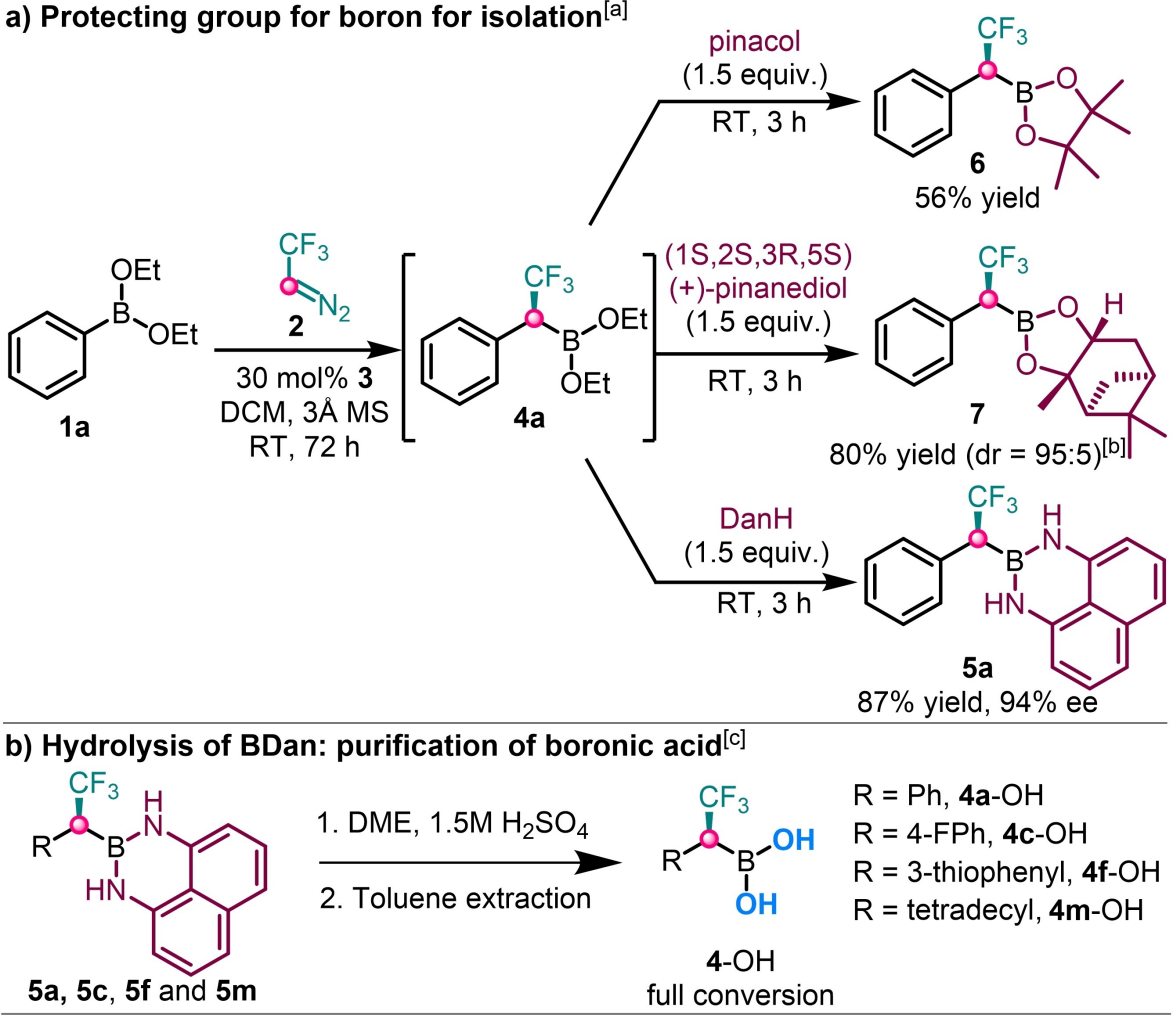
Conversion of boronate functionality. [a] Unless otherwise stated: the homologation was done using **1 a** (0.1 mmol), **2** (0.3 mmol) and **3** (0.03 mmol, 30 mol %) in 1.0 mL of DCM at r.t. for 72 h [b] d.r. based on ^19^F NMR. [c] **5** (0.1 mmol) was hydrolyzed with 1.5 M H_2_SO_4_ in DME, and then **4** was extracted with toluene under Ar.

Under the optimal conditions, we investigated the effects of different protecting groups on boron (Scheme 2a). The protection with aliphatic diols, such as pinacol and pinanediol could be achieved with acceptable yields. However, the Bpin product **6** was unstable on silica gel and obtained only 56 % yield after flash chromatography. The purification losses are probably due to oxidation of the Bpin group. The product Bpinane **7** was obtained by 80 % yield using commercially available optically pure pinanediol (Scheme 2b). An advantage of preparing a pinanediol derivative, such as **7** is that the ee of the reaction can be determined on the basis of d.r. obtained from the ^19^F NMR spectrum. The accuracy of the determination of the ee by this method is, of course, lower than by chiral chromatography (see ee determined for **5 a**). Yet, preparation of pinanediol derivatives is very simple (Scheme 2b) and the ee of a homologated product can be determined even if the chromatography analysis of the BDan derivative is not possible by any reason. Dan‐protected boron compound **5 a** was separated by silica chromatography and was obtained as a bench‐stable crystalline solid. The Bdan derivative **5 a** could be hydrolyzed under acidic conditions to generate the enantiopure free‐boronic acid (Scheme 2b). The generality of this deprotection was evaluated with another aromatic (**5 c**), heterocyclic (**5 f**), and aliphatic (**5 m**) α‐CF_3_ chiral Bdan derivatives. All of them provided a free boronic acid in full conversion (Scheme 2b). Thus, the crude and oxidation sensitive diethyl ester **4 a** can be purified by Bdan protection (**5 a**) followed by silica gel chromatography and subsequent hydrolysis (Scheme 2b) to obtain **4 a**‐OH, which is also highly oxidation sensitive. Conversely, Bpin **6** and pinanediol derivative **7** could not be hydrolyzed to obtain purified organoboronic acid derivatives.

**Scheme 3:**
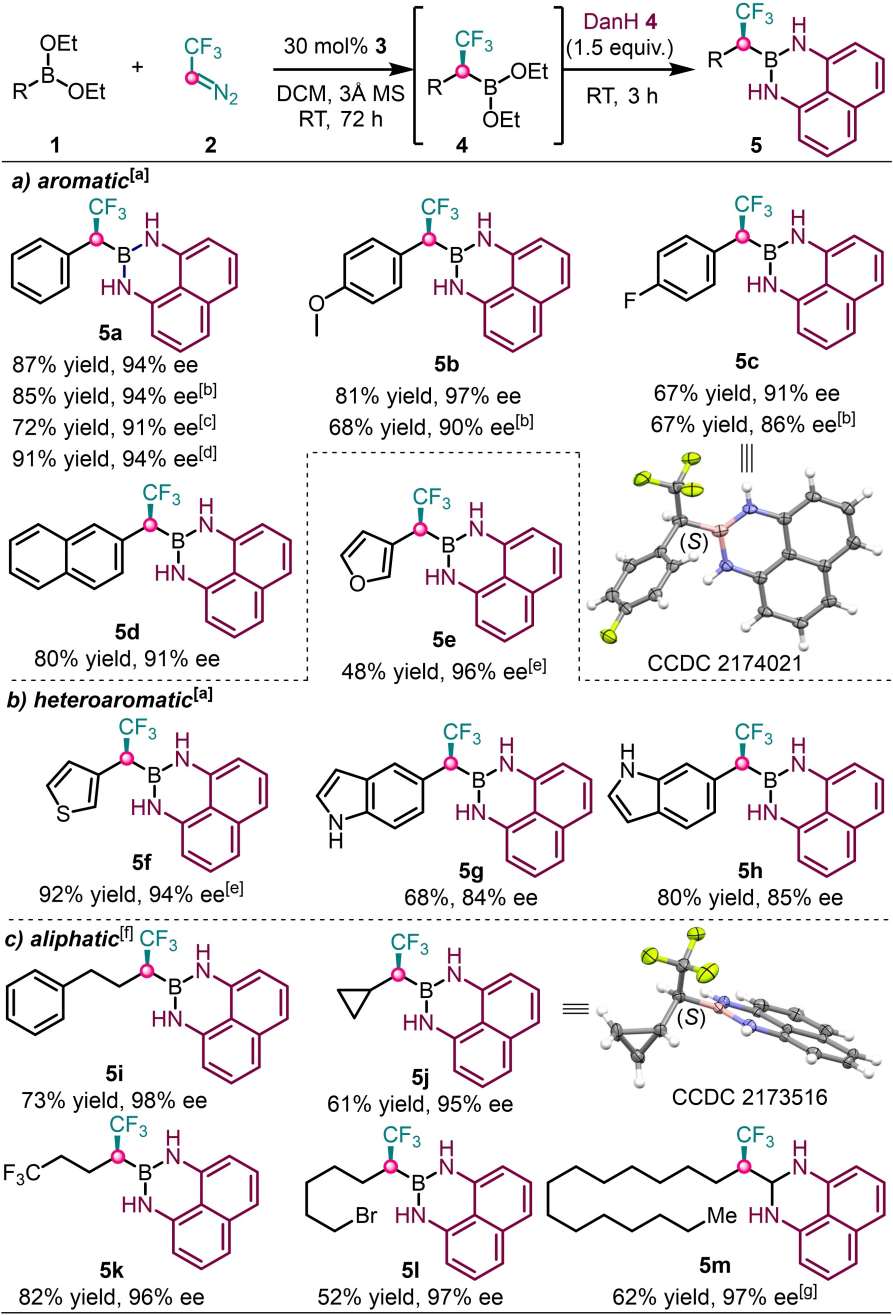
Scope of asymmetric homologation. [a] Unless otherwise stated the reactions were conducted at room temperature for 72 h using 30 mol % catalyst and 3 h for DanH protection. [b] with 20 mol % catalyst. [c] with 10 mol % catalyst. [d] 1 mmol scale. [e] reaction time 48 h. [f] reactions at 50 °C for 48 h and 2 h for DanH protection. [g] reaction time 54 h.

The substrate scope of the reaction was studied under the above optimal conditions (Table 1). The reactions of diethyl arylboronates with electron‐donating and withdrawing groups on the aromatic ring readily gave the corresponding α‐CF_3_ Bdan derivatives **5 b**–**5 c** in good yields and ee values (Scheme 3a). Despite the similar results for the reaction of **1 a** with 30 and 20 mol % catalyst loading (entries 1–2), a different trend was observed for reaction of substituted derivatives **1 b** and **1 c**. Either the yield (**5 b**) or the selectivity (**5 c**) dropped when the catalyst loading was decreased. Thus, we further proceeded with a 30 mol % catalyst to scale‐up the reaction (1 mmol) as well as for the studies of the scope of the reaction. The absolute configuration (S) of **5 c** was determined by X‐ray crystallography. The 2‐naphthylboronic ester **1 d** also reacted smoothly to provide **5 d** in good yield (80 %) and high selectivity (91 % ee). In addition, even heteroaromatic boron precursors underwent asymmetric homologation to deliver the corresponding chiral α‐CF_3_ Bdan derivatives (Scheme 3b). 3‐Furyl (**1 e**) and 3‐thiophenyl (**1 f**) boronic esters reacted faster than **1 a**, and thus the reactions were carried out for 48 h. The 5‐ and 6‐ indolylboronic esters (**5 g**–**5 h**) were obtained with lower selectivity (84–85 % ee) than **5 a** (94 % ee).

Not only aromatic but even aliphatic boronic esters reacted readily to deliver the corresponding chiral α‐CF_3_ Bdan derivatives. However, the reaction conditions had to be slightly modified to get high yields and selectivities (Scheme 3c). Thus, the reaction temperature for homologation of aliphatic boronic esters is increased to 50 °C. The required increase of the reaction temperature from room temperature to 50 °C is probably due to the lower reactivity of aliphatic boronic esters compared to the corresponding aromatic analogs. Phenethyl (**5 i**) and cyclopropyl (**5 j**) α‐CF_3_ Bdan derivatives were obtained in good yields (73 and 61 %) and excellent selectivities (98 and 95 % ee). The absolute configuration of **5 j** (S) obtained by X‐ray diffraction. Based on the absolute configuration determined for **5 d** and **5 j** and the similarities of the substrates and the reaction conditions, we assume the same absolute configuration of the other products as well. The trifluoromethyl (**5 k)**, the bromo (**5 l**) and sterically bulkier tetradecyl (**5 m)** substituted α‐CF_3_ Bdan derivatives were readily obtained in good yields (52–82 %) and excellent ee values (96–97 % ee).

**Scheme 4:**
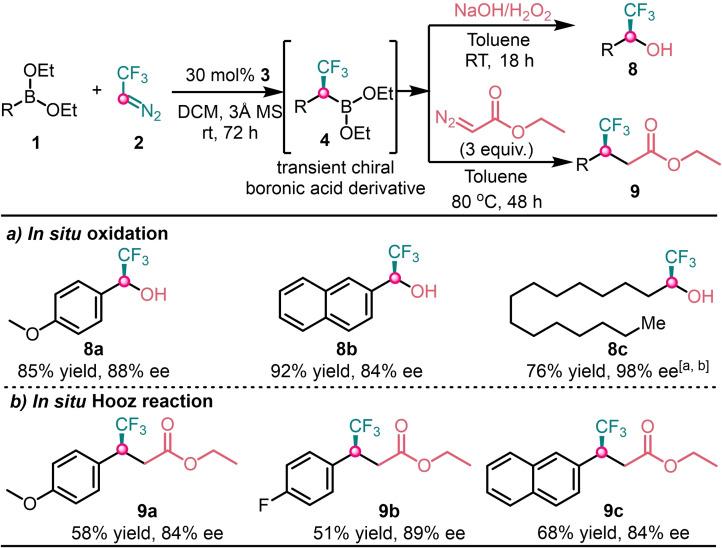
Interconversion of transient chiral boronic acid derivative. [a] the homologation reaction was carried out at 50 °C for 54 h. [b] oxidation was done in THF for 24 h.

To demonstrate the synthetic utility of the present method we tested the in situ oxidation and Hooz‐type^[21]^ reaction with transient chiral boronic acid derivative **4** (Scheme 4). The stereoselective in situ oxidation of chiral aryl and alkyl boron compounds yielded the corresponding chiral α‐CF_3_ aryl and alkyl alcohols **8 a**–**8 c** with 76–92 % yields and 84–98 % ee (Scheme 4a). In situ Hooz‐type reactions were performed with ethyl diazoacetate (EDA) to obtain chiral *β*‐CF_3_ carboxylate derivatives **9 a**–**9 c** in moderate yields and selectivities.

**Scheme 5:**
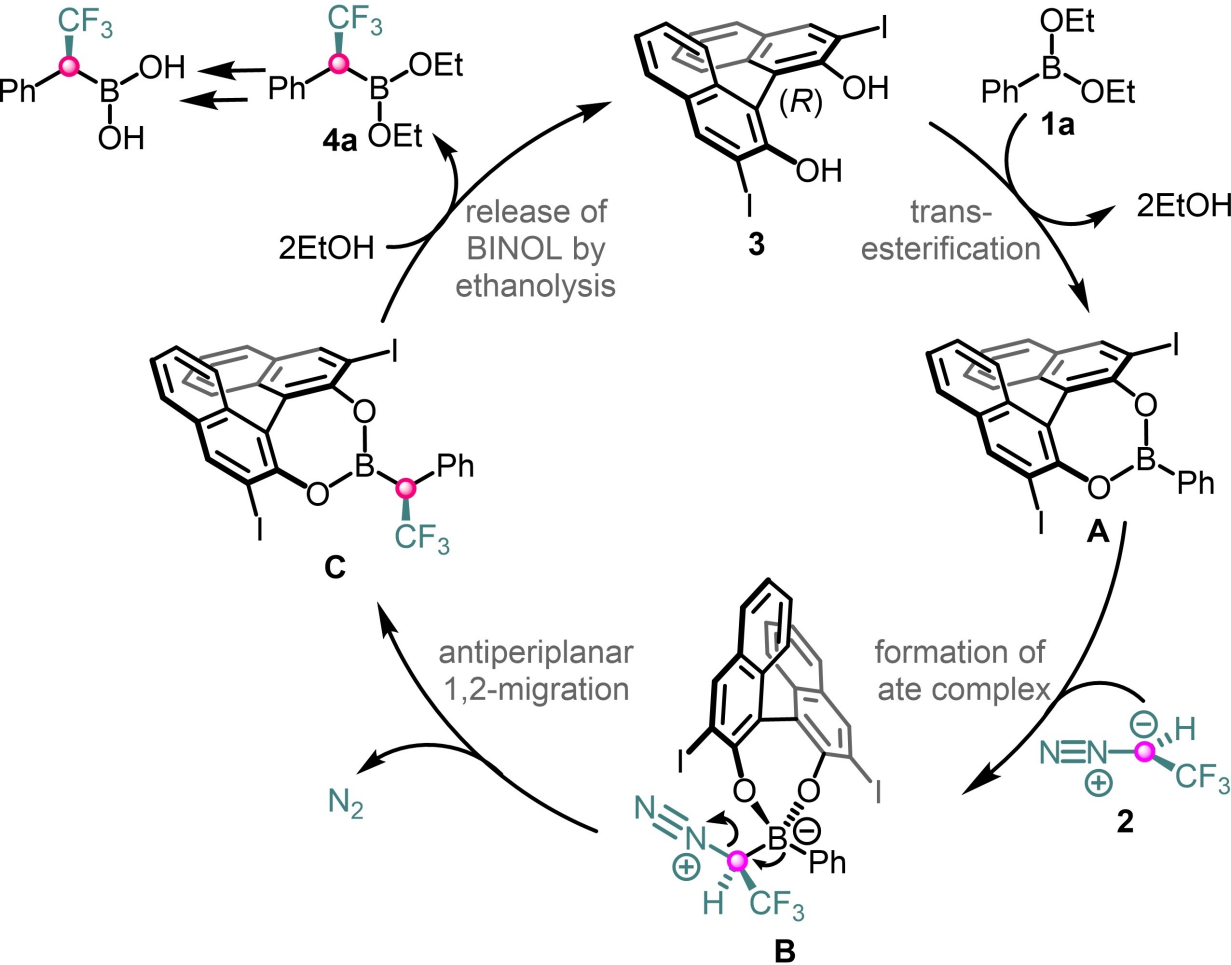
Catalytic cycle for enantioselective 1,2‐borotropic migration.

The proposed catalytic cycle (Scheme 5) is partially based on our previous assumptions^[19]^ as well as on DFT modelling for homologation of vinylboronates by Wang, Wei and co‐workers.^[22]^ The initial transesterification of BINOL **3** with **1 a** leads to chiral boron intermediate **A**. Intermediate **A** is more reactive than **1 a**. A possible explanation is that the exchange of the alkyl group (**1 a**) to an aromatic moiety (**A**) on the boron substantially increases its Lewis acidity. This BINOL ester activation is essential for the high enantioselectivity as the reaction of **1 a** with **2** gives the racemic product with poor yield (Table 1, entry 6). A relatively facile transesterification is also important. The rate of transesterification of various boronic esters is apparently different. This could be the explanation that **1 a** reacted with high yield under the applied reaction conditions (Table 1 entry 1), while bulkier isopropyl ester gave **5 a** with low yield (entry 8) and the pinacol chelated analog did not reacted at all (entry 7). Intermediate **A** and **2** forms ate complex **B** in the stereoinduction step of the process. Then the alkyl or aryl group undergoes stereoselective 1,2‐migration to afford **C**. Subsequently, ethanolysis of **C** gives the product **4 a** and recovers the catalyst (**3**).

## Conclusion

In summary, we have presented a versatile method for the asymmetric homologation of alkyl, aryl, and heterocyclic boronic acids using BINOL as a catalyst. Application of trifluorodiazoethane **2** as a nucleophilic partner successfully delivered a variety of α‐trifluoromethylated boronic acid derivatives. In situ functional group transformation or consecutive reaction with transient chiral boronic acid delivered the corresponding chiral α‐CF_3_ alcohols or *β*‐CF_3_ carboxylates with good enantioselectivity.

## Experimental Section

### General procedure for the asymmetric homologation

Method A for the synthesis of **5 a**–**5 h**: A reaction tube was charged with catalyst **3** (0.03 mmol) and brought into the glovebox. Diethyl arylboronate **1** **(a**–**h)** (0.1 mmol, 1 equiv.), CF_3_‐diazomethane **2** (0.3 mmol, 3 equiv.) in dichloromethane and molecular sieves (20 mg) were added. The total volume of reaction mixture was maintained to 1 mL. The reaction mixture was stirred at room temperature for 72 h and then DanH (0.15 mmol, 1.5 equiv.) was added inside a glovebox and the reaction mixture stirred for another 4 h at room temperature. The product was isolated by silica gel chromatography.

Method B for the synthesis of **5 i**–**5 m**: A stock solution of diethyl alkylboronate **1** **(i**–**m)** in dichloromethane (0.33 mL, 0.1 mmol, 1 equiv.) was added to a reaction tube containing catalyst **3** (0.03 mmol). Then CF_3_‐diazomethane **2** (0.3 mmol, 3 equiv.) in dichloromethane and molecular sieves (20 mg) were added. The total volume of reaction mixture was maintained to 0.8 mL and was stirred at 50 °C for 48 h. Then, DanH (0.15 mmol) was added inside a glovebox and the reaction mixture was stirred for another 2 h at room temperature. The product was isolated by silica gel chromatography.

All other experimental data and characterization is provided in the Supporting Information.

Deposition Numbers 2174021 (for **5 c**) and 2173516 (for **5 j**) contain the supplementary crystallographic data for this paper. These data are provided free of charge by the joint Cambridge Crystallographic Data Centre and Fachinformationszentrum Karlsruhe Access Structures service.

## Conflict of interest

The authors declare no conflict of interest.

1

## Supporting information

As a service to our authors and readers, this journal provides supporting information supplied by the authors. Such materials are peer reviewed and may be re‐organized for online delivery, but are not copy‐edited or typeset. Technical support issues arising from supporting information (other than missing files) should be addressed to the authors.

Supporting Information

## Data Availability

The data that support the findings of this study are available in the supplementary material of this article.
