# Supplementary material for: Asymmetric Organocatalytic Homologation: Access to Diverse Chiral Trifluoromethyl Organoboron Species
Source: Chemistry. 2022 Aug 18;28(58):e202202059. doi: 10.1002/chem.202202059 (PMC9804810; doi:10.1002/chem.202202059)
Supplement: Supplementary file 1 — Supporting Information [file CHEM-28-0-s001.pdf]

Chemistry–A European Journal

Supporting Information

Asymmetric Organocatalytic Homologation: Access to Diverse Chiral Trifluoromethyl Organoboron Species

Ramasamy Jayarajan, Tautvydas Kireilis, Lars Eriksson, and Kálmán J. Szabó*

Table of contents

1. General information 2
2. Experimental procedure 2
   Preparation of diethyl arylboronate 2
   Preparation of diethyl alkylboronate 3
   Hydrolysis of BDan derivative 4
   General procedure C: In situ oxidation of chiral boronic ester 7
   General procedure D: In situ Hooz-type reaction 7
3. NMR Spectra 36
4. References 85

1. General information

All reactions were performed under inert atmosphere unless otherwise stated. All boronic acids were obtained from commercial sources. Iodo-BINOL **3** was prepared by a reported procedure.[1] For column chromatography, silica gel (35-70 microns) was used. TLC was performed using aluminium backed plates pre-coated (0.25 mm) with Silica Gel 60 F254 with a suitable solvent system and was visualized using UV fluorescence and/or developed with permanganate stain. All isolated compounds were characterized by ^{1}H, ^{13}C, ^{19}F and ^{11}B NMR spectroscopy using Bruker 400 MHz and 500 MHz spectrometers. Copies of the NMR spectra can be found in the end of this Supporting Information. All ^{1}H NMR experiments were reported in units, parts per million (ppm), and were measured relative to the signals for residual chloroform (7.26 ppm) in the deuterated solvent, unless otherwise stated. All ^{13}C NMR spectra were reported in ppm relative to deuterated chloroform (77.16 ppm), unless otherwise stated. All crude NMR analysis were performed by using trifluorotoluene as the internal standard. High resolution mass spectrometry (HRMS) was obtained using the APCI and ESI techniques. X-ray diffraction was performed using a Bruker D8 ADVANCE kappa geometry diffractometer equipped with a Bruker Photon 100 CMOS detector.

Chiral SFC analysis: Unless otherwise stated all the enantiomeric excess analysis were performed with chiral SFC. Chiral SFC analysis was performed using Chiralpak IA-3, Chiralpak IB N-3, Chiralpak IC-3 and Chiralcel OJ-3, eluting with MeOH/CO_2 or ^{i}PrOH/CO_2 (SFC) and monitored by DAD (Diode Array Detector). Retention times (tR) are quoted in minutes.

Chiral GC analysis: Chiral GC was performed for products **9a** and **9b** using a GC-MS (30 m column, helium gas carrier at 1 mL/min, constant pressure).

2. Experimental procedure

Preparation of diethyl arylboronates 1a-1h:

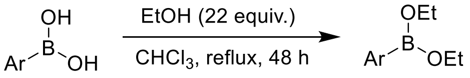

1a - 1h

1a, Ar = C_6H_5
1b, Ar = 4-OMe C_6H_4
1c, Ar = 4-F C_6H_4
1d, Ar = 2-naphthyl
1e, Ar = 3-furyl
1f, Ar = 3-thiophenyl
1g, Ar = 5-indolyl
1e, Ar = 6-indolyl

Diethyl arylboronate was prepared by using reported procedure.[2] Aryl boronic acid (4 mmol) was refluxed with chloroform (12.5 ml) and ethanol (6.3 mL) in a round bottom flask that fitted with Soxhlet apparatus containing molecular sieves (4 Å). After 48 h the excess solvent was removed under the vacuum to yield the corresponding diethyl arylboronate **1a-1h** which was used for homologation reactions without further purifications. The diisopropyl arylboronate (Table 1, entry 8) was prepared by the same procedure using isopropyl alcohol.

Preparation of diethyl alkylboronates 1i-1m:

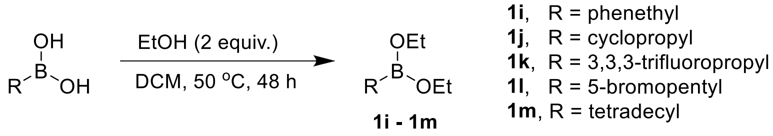

Alkylboronic acid (3.5 mmol) and molecular sieves (3 Å) were taken in a round bottom flask. Dichloromethane (4 mL), ethanol (7 mmol) were added and heated at 50 °C for 24 h. After 24 hours the excess solvent was carefully removed under reduced pressure to provide the corresponding diethyl alkylboronate **1i – 1m** (note that compounds **1j** and **1k** are volatile). The stock solution was prepared in dichloromethane (0.33 mmol/mL) and used for homologation reaction without further purification.

Preparation of 2-diazo-1,1,1-trifluoroethane (2):

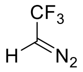

Compound **2** was synthesized according to a slightly modified procedure reported by Molander and co-workers.[3] Trifluoroethylamine hydrochloride (1.355 g, 10 mmol) and sodium nitrite (0.759 g, 11 mmol) were taken in a round bottom flask and purged with argon. Dichloromethane (10 mL) was added to the round bottom flask, cooled to 0 °C and stirred for 30 min. Then, degassed water (1.5 mL, 83 mmol) was added and the mixture was stirred for 2 h at 0 °C, then for an additional 30 min at 10 °C. The organic layer was transferred to another round bottom flask containing anhydrous sodium sulphate (5 g) and stirred for 1 h at 0 °C. The obtained compound **2** was transferred to a vial containing activated molecular sieves (3 Å pellets) and stored in a freezer for the further use. The concentration of compound **2** was analysed by ^{19}F NMR using trifluorotoluene as an internal standard.

General procedure A: Asymmetric homologation of diethyl arylboronate (5a-5h):

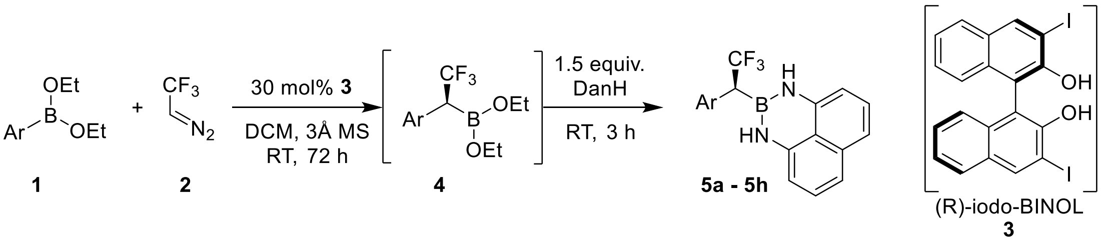

A reaction tube was charged with catalyst **3** (0.03 mmol) and brought into the glovebox. Diethyl arylboronate **1** (0.1 mmol), CF_3-diazomethane **2** (0.3 mmol) in dichloromethane and molecular sieves (20 mg) were added. The total volume of reaction mixture was maintained to 1 mL. The reaction mixture was stirred at room temperature for 72 h and DanH (0.15 mmol) was added inside the glovebox and stirred for another 4 h at room temperature. The product was isolated by silica gel chromatography.

General procedure B: Asymmetric homologation of diethyl alkylboronate (5i-5m):

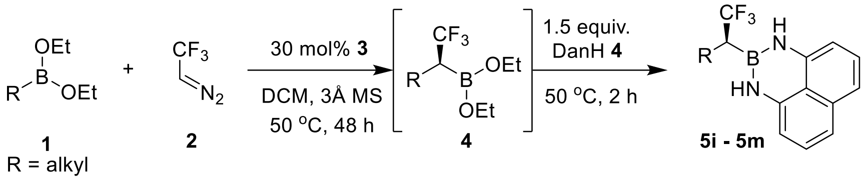

A reaction tube was charged with catalyst **3** (0.03 mmol) and brought into the glovebox. Diethyl alkylboronate **1** (0.1 mmol, 0.33 mL) in dichloromethane, CF_3-diazomethane **2** (0.3 mmol) in dichloromethane and molecular sieves (20 mg) were added. The total volume of reaction mixture was maintained to 0.8 mL. The reaction mixture was stirred at 50 °C for 48 h and then DanH (0.15 mmol) was added inside the glovebox and stirred for another 2 h at RT. The product was isolated by silica gel chromatography.

Preparation of racemates: The racemic mixtures of CF_3-homologated BDan products were prepared by following the above procedures A or B except that an equimolar mixture of (R) and (S) catalyst **3** was used. The minor deviations from 50/50 ratio of the S/R enantiomers may be due to weighing errors.

Hydrolysis of BDan derivative:

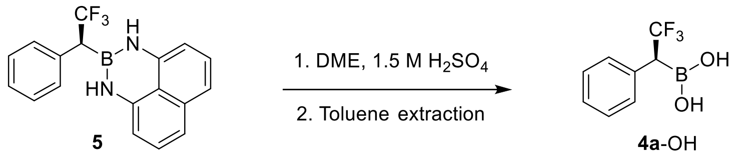

Benzyl BDan **5a** (0.1 mmol) was taken in a reaction tube under argon. Degassed dimethoxyethane (0.6 mL) was added followed by 1.5M sulfuric acid (0.5 mL). The mixture was stirred overnight at room temperature. Then, 0.5 M hydrochloric acid and toluene-d_8 was added and the mixture was shaken vigorously. The organic layer was passed through a phase separator. Conversion of BDan **5a** into free boronic acid **4a-**OH was found to be >99% based on ^{19}F NMR analysis (see ^{1}H, ^{13}C, ^{19}F and ^{11}B NMR below). A similar procedure was followed for the hydrolysis of the other BDan compounds (**5c, 5f** and **5m**).

^{1}H NMR (toluene-d_8, 400 MHz) of compound **4a**-OH

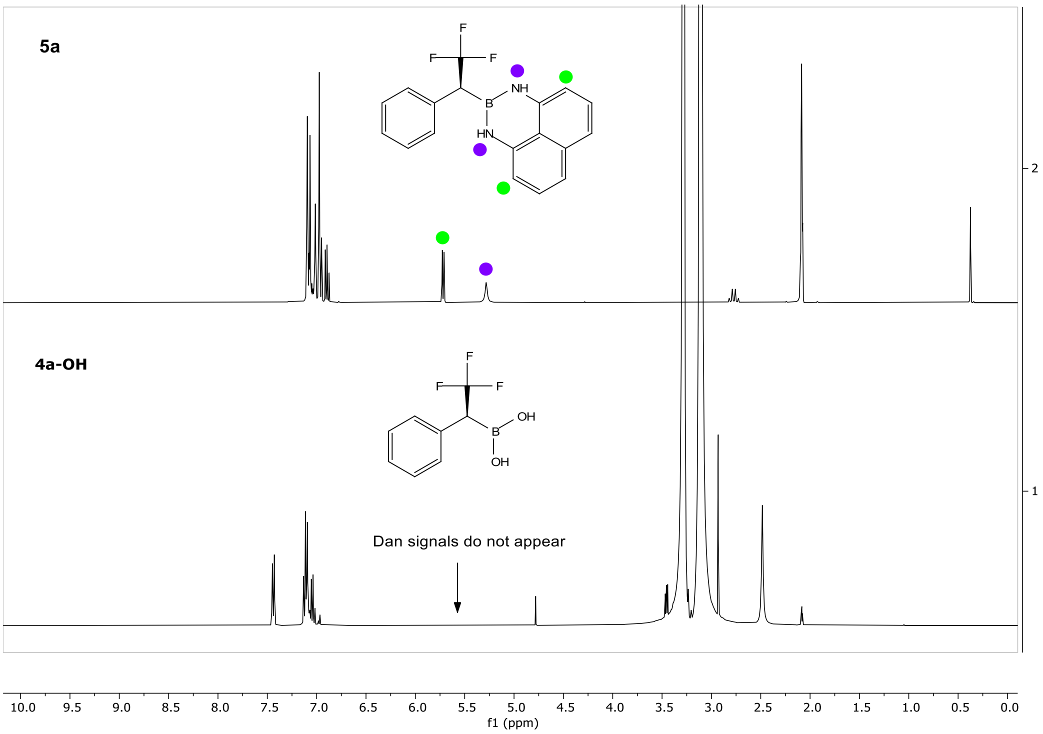

^{13}C NMR (toluene-d_8, 101 MHz) of compound **4a**-OH

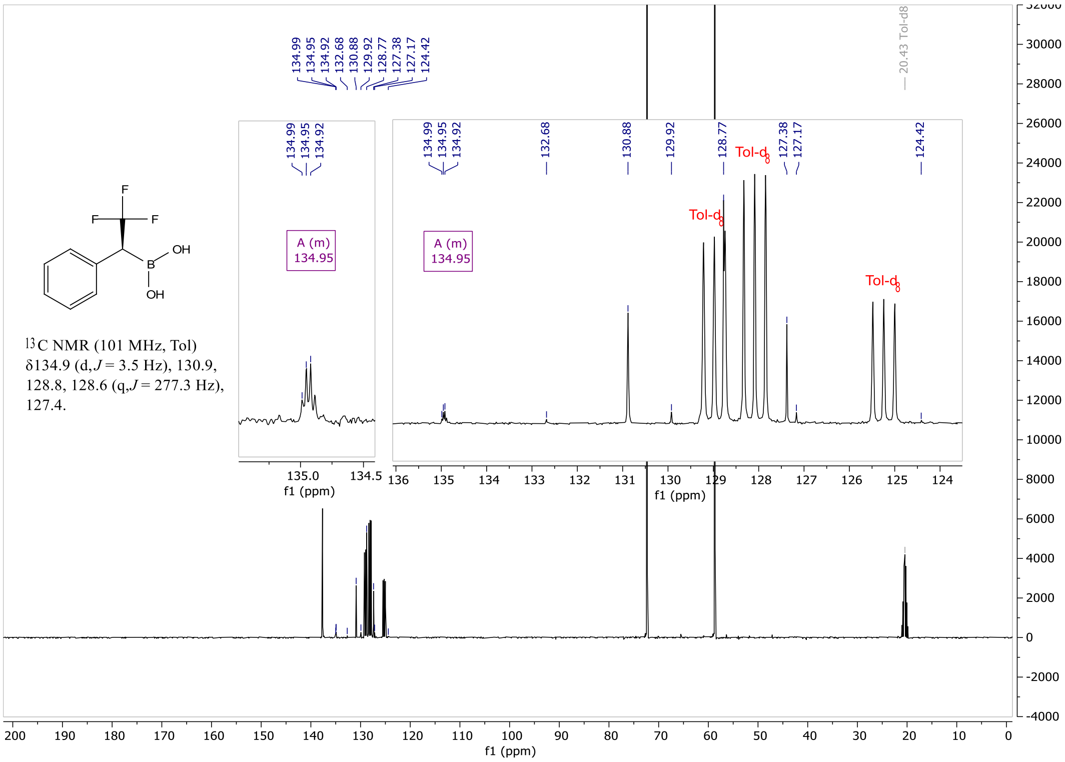

^{19}F NMR (toluene-d$_8$, 377 MHz) of compound **4a**-OH

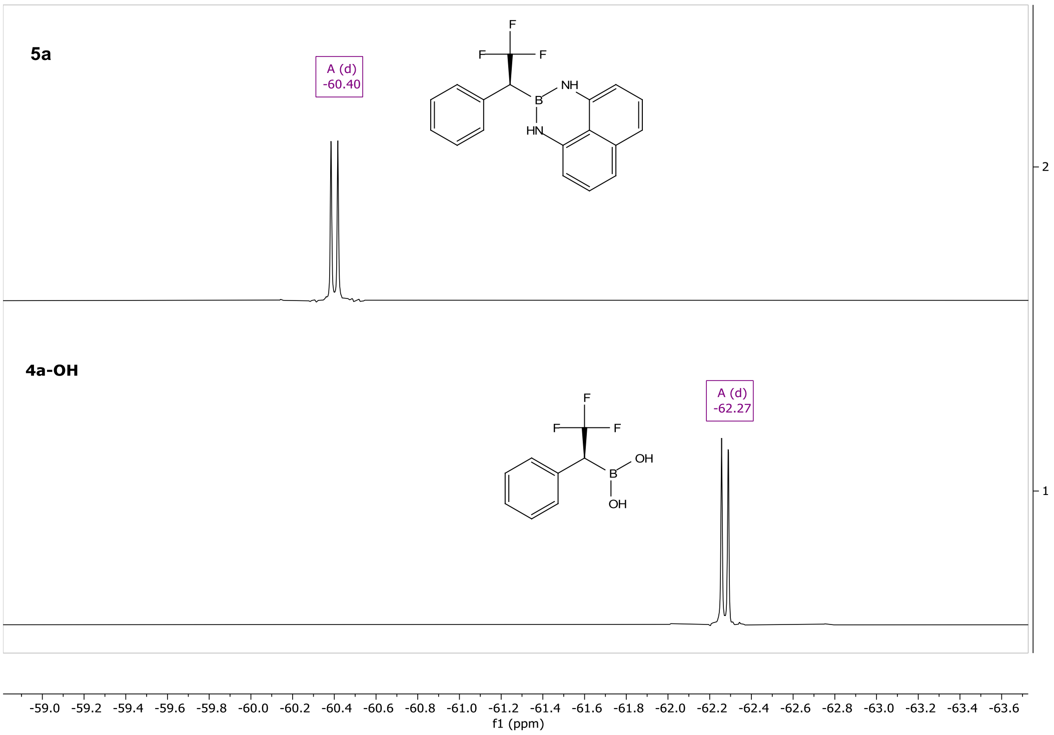

^{11}B NMR (toluene-d$_8$, 128 MHz) of compound **4a**-OH

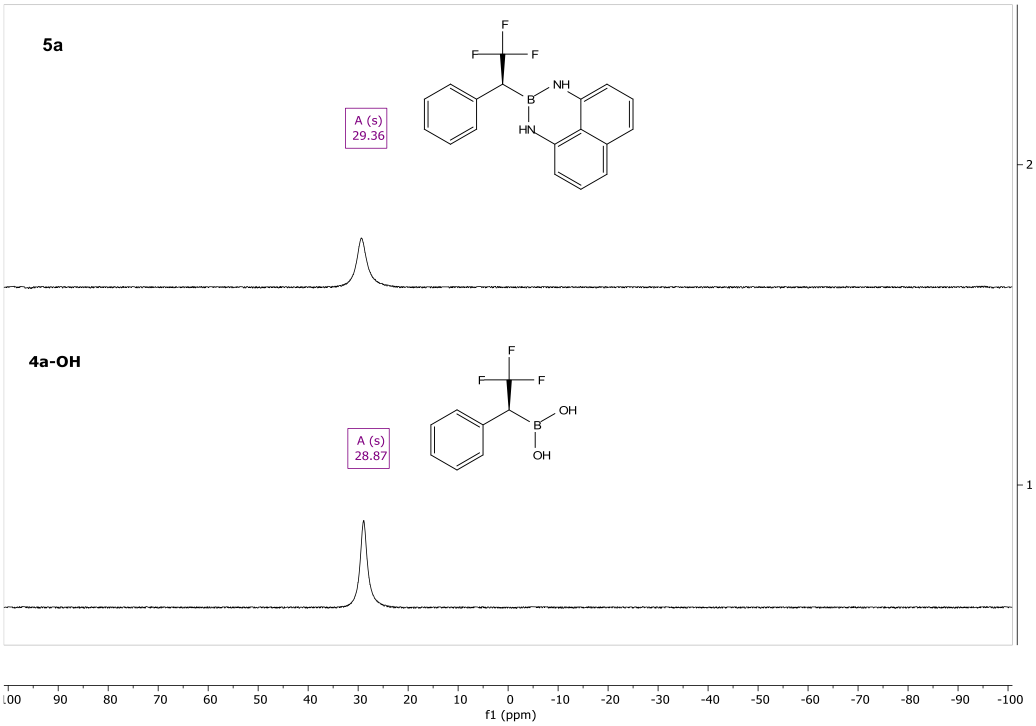

General procedure C: In situ oxidation of chiral boronic ester

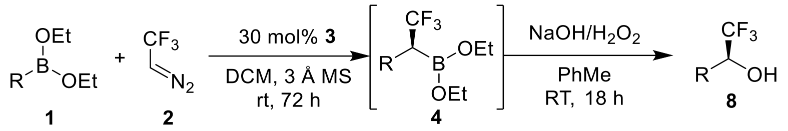

Compound **8** was prepared by the general procedure A except that after 72 h, dichloromethane was evaporated by Ar blow untill the volume reduced to 0.1 mL and toluene (0.5 mL) was added. Then, 3M NaOH (0.3 mmol, 0.1 mL) and H_2O_2 (0.3 mmol, 0.1 mL, 35 wt. % in H_2O) were added under 0 ^oC. Then, the reaction mixture was stirred at room temperature for overnight. The reaction mixture was acidified with 1M HCl and water (3 mL) was added. The organic layer was extracted with diethyl ether and the aqueous layer was washed 3 times with diethyl ether. The combined organic phase was passed through phase separator and the solvent was evaporated under reduced pressure. The chiral CF_3-alcohol was isolated by silica gel chromatography.

General procedure D: In situ Hooz-type reaction

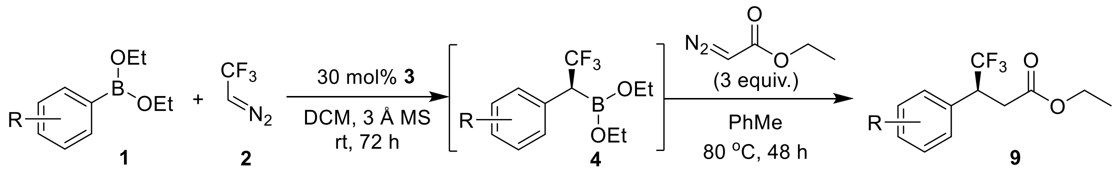

The general procedure A was used except that after 72 h, dichloromethane was evaporated by argon blow and toluene (0.65 mL) was added. Subsequently, molecular sieves (20 mg) and ethyl diazoacetate (0.3 mmol, 0.032 mL) were added and the reaction was stirred at 80 °C for 48 h. Then the reaction mixture was quenched with saturated ammonium chloride solution (0.5 mL). Deionized water (1 mL) was added and the organic phase was extracted with diethyl ether. Combined organic layer was passed through the phase separator and concentrated under vacuum. The crude mixture was purified by silica gel chromatography to provide compound **9**.

(S)-2-(2,2,2-trifluoro-1-phenylethyl)-2,3-dihydro-1H-naphtho[1,8-de][1,3,2]diazaborinine (5a):

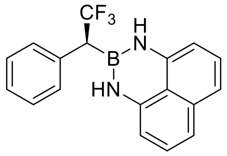

This compound was obtained according to the above general procedure A. Product **5a** was isolated in 87% yield (28.3 mg, 0.087 mmol) as white solid by silica gel chromatography using pentane/dichloromethane 4:1 solvent system as eluent. Product **5a** was isolated in 91% yield (298.0 mg, 0.914 mmol) as white solid by silica gel chromatography using pentane/dichloromethane 4:1 solvent system as eluent (1 mmol scale reaction); mp 135-138 °C.

^{1}H NMR (400 MHz, $CDCl_3$) δ 7.41 – 7.33 (m, 5H), 7.09 (dt, *J* = 14.9, 8.1 Hz, 4H), 6.30 (d, *J* = 7.0 Hz, 2H), 5.68 (s, 2H), 3.28 (q, *J* = 12.2 Hz, 1H). **^{13}C NMR** (101 MHz, $CDCl_3$) δ 140.1, 136.3, 133.3 (q, *J* = 3.0 Hz), 130.1, 129.3, 128.0, 127.8 (q, *J* = 278.0 Hz), 127.7, 119.8, 118.5, 106.5, 41.5. **^{19}F NMR** (377 MHz, $CDCl_3$) δ -60.49 (d, *J* = 12.3 Hz).**^{11}B NMR** (128 MHz, $CDCl_3$) δ 29.08. **HRMS** (pos. APCI) *m/z* calcd for $C_{18}H_{15}BF_3N_2$: 327.1278 $[M+H]^+$; found: 327.1280. $[\alpha]_D^{27}$ −53.2 (*c* 0.25, $CHCl_3$).

Determination of *ee* by Chiral SFC: Daicel CHIRALPAK OJ-3, 25 °C, 0.46 cm ϕ, 25 cm column, 10% *i*PrOH in CO_2, flow rate: 1.2 mL/min; tR: 5.06 min (minor enantiomer), 8.40 min (major enantiomer); ee = 94%.

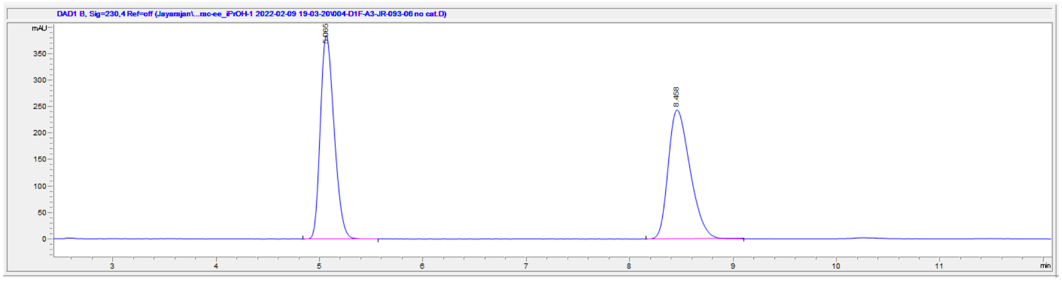

Signal 2: DAD1 B, Sig=230,4 Ref=off

Peak #	RetTime [min]	Type	Width [min]	Area [mAU*s]	Height [mAU]	Area %
1	5.065	BV R	0.1438	3551.77124	384.24765	49.7778
2	8.458	BB	0.2279	3583.48535	243.27785	50.2222
Totals :				7135.25659	627.52550	

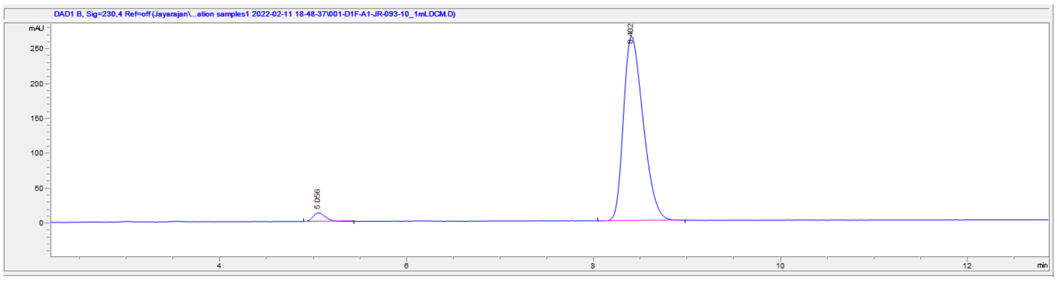

Signal 2: DAD1 B, Sig=230,4 Ref=off

Peak #	RetTime [min]	Type	Width [min]	Area [mAU*s]	Height [mAU]	Area %
1	5.056	BB	0.1395	114.50291	12.43042	2.8787
2	8.402	BB	0.2268	3863.07935	263.87424	97.1213
Totals :				3977.58225	276.30466	

(S)-2-(2,2,2-trifluoro-1-(4-methoxyphenyl)ethyl)-2,3-dihydro-1H-naphtho[1,8-de][1,3,2]diazaborinine (5b):

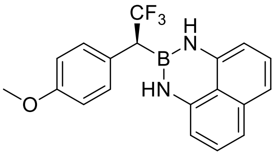

This compound was obtained according to the above general procedure A. Product **5b** was isolated in 81% yield (29.0 mg, 0.081 mmol) as white solid by silica gel chromatography using pentane/dichloromethane 3:1 solvent system as eluent; mp 121-123 °C.

^{1}H NMR (400 MHz, $CDCl_3$) δ 7.25 (d, *J* = 8.2 Hz, 2H), 7.08 (dt, *J* = 15.8, 8.1 Hz, 4H), 6.93 (d, *J* = 8.6 Hz, 2H), 6.30 (d, *J* = 7.0 Hz, 2H), 5.68 (s, 2H), 3.83 (s, 3H), 3.21 (q, *J* = 12.3 Hz, 1H). **^{13}C NMR** (101 MHz, $CDCl_3$) δ 159.4, 140.2, 136.3, 131.3, 127.9 (q, *J* = 277.9 Hz), 127.7, 125.0 (q, *J* = 3.2 Hz), 119.80, 118.5, 114.7, 106.5, 55.4, 40.6. **^{19}F NMR** (377 MHz, $CDCl_3$) δ -61.04 (d, *J* = 12.3 Hz). **^{11}B NMR** (128 MHz, $CDCl_3$) δ 29.15. **HRMS** (pos. APCI) *m/z* calcd for $C_{19}H_{17}BF_3N_2O$: 357.1384 $[M+H]^+$; found: 357.1385. $[\boldsymbol{\alpha}]_{\boldsymbol{D}}^{\mathbf{26}}$ −46.8 (c 0.25, $CHCl_3$).

Determination of *ee* by Chiral SFC: Daicel CHIRALPAK OJ-3, 25 °C, 0.46 cm *ϕ*, 25 cm column, 20% *i*PrOH in CO_2, flow rate: 1.2 mL/min; *t*R: 8.75 min (minor enantiomer), 15.49 min (major enantiomer); *ee* = 97%.

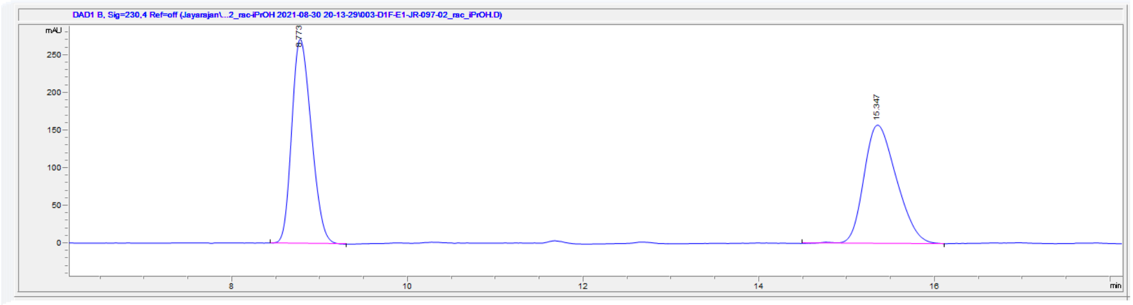

Signal 2: DAD1 B, Sig=230,4 Ref=off

Peak #	RetTime [min]	Type	Width [min]	Area [mAU*s]	Height [mAU]	Area %
1	8.773	BB	0.2405	4199.30859	271.37534	50.7833
2	15.347	VB R	0.3922	4069.76611	157.43924	49.2167
Totals :				8269.07471	428.81458	

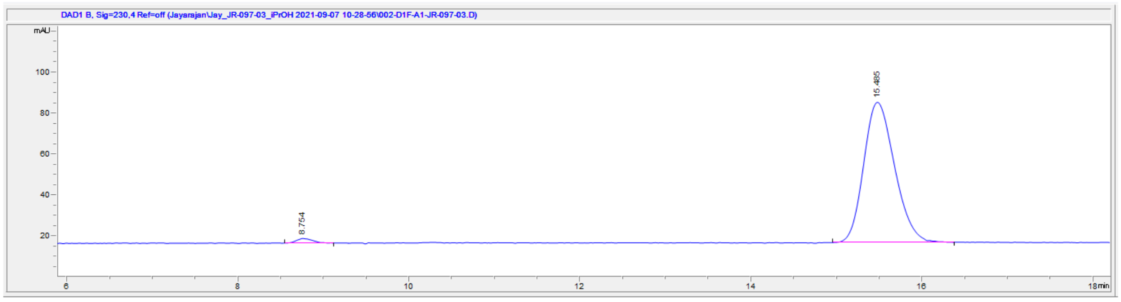

Signal 2: DAD1 B, Sig=230,4 Ref=off

Peak #	RetTime [min]	Type	Width [min]	Area [mAU*s]	Height [mAU]	Area %
1	8.754	BB	0.1663	30.07767	2.27393	1.7079
2	15.485	BB	0.3779	1730.97107	68.70319	98.2921
Totals :				1761.04873	70.97712	

(S)-2-(2,2,2-trifluoro-1-(4-fluorophenyl)ethyl)-2,3-dihydro-1H-naphtho[1,8-de][1,3,2]diazaborinine (5c):

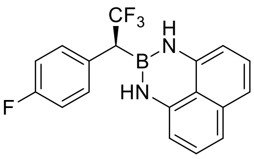

This compound was obtained according to the above general procedure A. Product **5c** was isolated in 67% yield (23.1 mg, 0.067 mmol) as white solid by silica gel chromatography using pentane/dichloromethane 4:1 solvent system as eluent; mp 111-114 °C.

^{1}H NMR (400 MHz, $CDCl_3$) δ ^{1}H NMR (400 MHz, $CDCl_3$) δ 7.31 (dd, *J* = 8.1, 5.5 Hz, 2H), 7.10 (p, *J* = 7.8 Hz, 6H), 6.31 (d, *J* = 7.0 Hz, 2H), 5.66 (s, 2H), 3.26 (q, *J* = 12.1 Hz, 1H). **^{13}C NMR** (101 MHz, $CDCl_3$) 162.6 (d, *J* = 247.3 Hz), 134.0, 136.3, 131.8 (d, *J* = 8.1 Hz), 129.0 (d, *J* = 3.1 Hz), 127.7, 124.9 (d, *J* = 277.8 Hz), 119.8, 118.7, 116.3 (d, *J* = 21.5 Hz), 106.6, 40.6. **^{19}F NMR** (377 MHz, $CDCl_3$) δ -60.94 (d, *J* = 12.1 Hz), -113.96 – -114.29 (m). **^{11}B NMR** (128 MHz, $CDCl_3$) δ 29.72. **HRMS** (pos. APCI) *m/z* calcd for $C_{18}H_{14}BF_4N_2$: 345.1184 $[M+H]^+$; found: 345.1186. $[\alpha]_D^{28}$ –57.6 (c 0.25, $CHCl_3$).

Determination of *ee* by Chiral SFC: Daicel CHIRALPAK OJ-3, 25 °C, 0.46 cm ϕ, 25 cm column, 10% *i*PrOH in CO_2, flow rate: 1.2 mL/min; *t*R: 5.33min (minor enantiomer), 7.36 min (major enantiomer); *ee* = 91%.

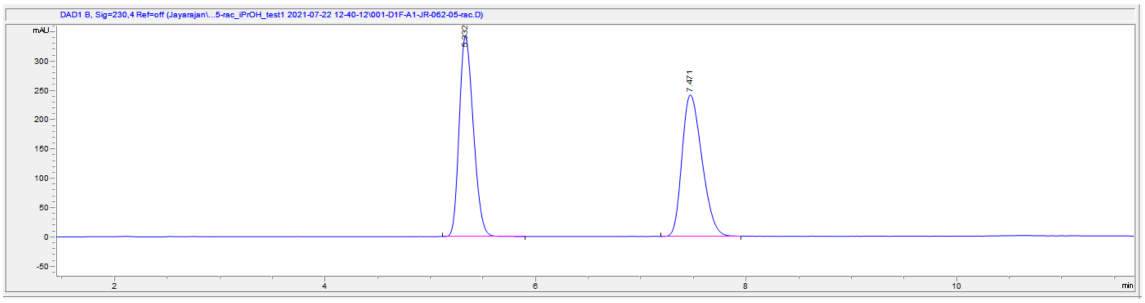

Signal 2: DAD1 B, Sig=230,4 Ref=off

Peak #	RetTime [min]	Type	Width [min]	Area [mAU*s]	Height [mAU]	Area %
1	5.332	BB	0.1448	3170.84448	339.89767	50.0636
2	7.471	BB	0.2056	3162.78711	240.17598	49.9364
Totals :				6333.63159	580.07365	

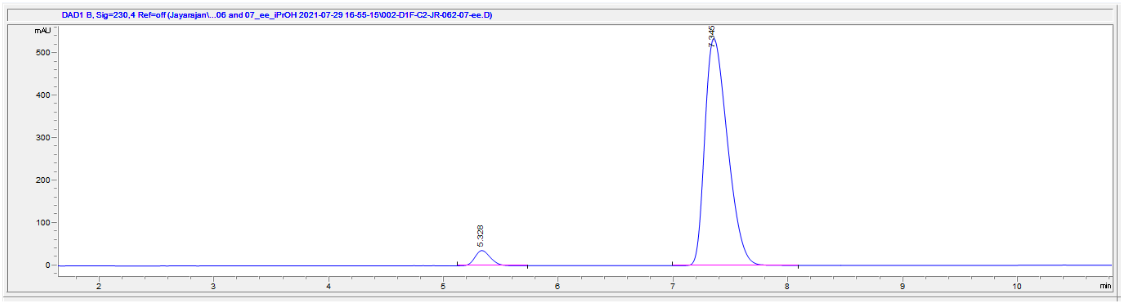

Signal 2: DAD1 B, Sig=230,4 Ref=off

Peak #	RetTime [min]	Type	Width [min]	Area [mAU*s]	Height [mAU]	Area %
1	5.328	BB	0.1459	336.34665	35.71892	4.3982
2	7.345	BB	0.2137	7311.06104	534.26135	95.6018
Totals :				7647.40768	569.98027	

X-ray structure of 5c (CCDC number: 2174021):

Sample preparation (vapor diffusion method): A small vial containing 10 mg of compound **5c** or **5j** in 0.2 mL of 5:1 (pentane:EA) was placed inside the big vial. The outer vial was filled with pentane and capped tightly. Over two weeks in the freezer, the formation of crystals was observed.

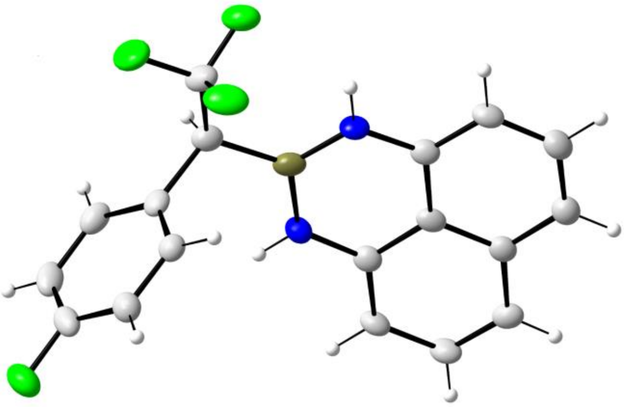

Table 1 Crystal data and structure refinement for compound 5c

Empirical formula	$C_{18}H_{13}BF_4N_2$
Formula weight	344.11
Temperature / K	100
Crystal system	monoclinic
Space group	P 2_1 (nr. 4)
a / Å	10.9753(8)
b / Å	5.8509(4)
c / Å	12.1185(9)
α / °	90
β / °	99.252(4)
γ / °	90
Volume / $Å^3$	768.07(10)
Z	2
ρ_{calc} / g cm^{-3}	1.488
μ / mm^{-1}	1.043
F(000)	352
Crystal size / mm^3	0.10 · 0.07 · 0.03
Radiation	CuKα (λ = 1.54184 Å)
2θ range for data collection / °	7.37 to 119.30
Index ranges	-12 ≤ h ≤ 12, -6 ≤ k ≤ 6, -13 ≤ l ≤ 13
Reflections collected	5641 [R_{int} = 0.0575, R_{sigma} = 0.0675]
Unique reflections	2168 (all data), 2002(I ≥ 2σ(I))
Data / restraints / parameters	2168, 1, 227
Goodness of fit on F^2	1.05
Final R indexes [I ≥ 2σ(I)]	R1 = 0.0439, wR2 = 0.1114
Final R indexes [all data]	R1 = 0.0492, wR2 = 0.1157
Largest diff peak and hole / e $Å^{-3}$	-0.210 … 0.245

(S)-2-(2,2,2-trifluoro-1-(naphthalen-2-yl)ethyl)-2,3-dihydro-1H-naphtho[1,8-de][1,3,2]diazaborinine (5d):

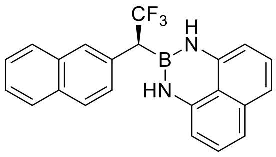

This compound was obtained according to the above general procedure A. Product **5d** was isolated in 80% yield (30.1 mg, 0.08 mmol) as white solid by silica gel chromatography using pentane/dichloromethane 4:1 solvent system as eluent; mp 142-145 °C.

^{1}H NMR (400 MHz, $CDCl_3$) δ 7.88 (t, *J* = 9.7 Hz, 3H), 7.82 (s, 1H), 7.65 – 7.51 (m, 2H), 7.45 (d, *J* = 8.4 Hz, 1H), 7.13 – 7.06 (m, 4H), 6.29 (d, *J* = 6.9 Hz, 2H), 5.72 (s, 2H), 3.46 (q, *J* = 12.2 Hz, 1H). **^{13}C NMR** (101 MHz, $CDCl_3$) δ 140.1, 136.3, 133.6, 132.8, 130.7 (q, *J* = 3.2 Hz), 129.3, 129.1, 128.0, 127.9, 127.7, 127.6, 126.8, 126.6, 125.1 (q, *J* = 278.2 Hz), 119.8, 118.6, 106.6, 41.8. **^{19}F NMR** (377 MHz, $CDCl_3$) δ -60.15 (d, *J* = 12.2 Hz).**^{11}B NMR** (128 MHz, $CDCl_3$) δ 29.43. **HRMS** (pos. APCI) *m/z* calcd for $C_{22}H_{17}BF_3N_2$: 377.1435 $[M+H]^+$; found: 377.1436. $[\boldsymbol{\alpha}]_D^{27}$ –79.2 (c 0.25, $CHCl_3$).

Determination of *ee* by Chiral SFC: Daicel CHIRALPAK OJ-3, 25 °C, 0.46 cm ϕ, 25 cm column, 20% *i*PrOH in CO_2, flow rate: 1.2 mL/min; *t*R: 7.08 min (minor enantiomer), 13.20 min (major enantiomer); *ee* = 91%.

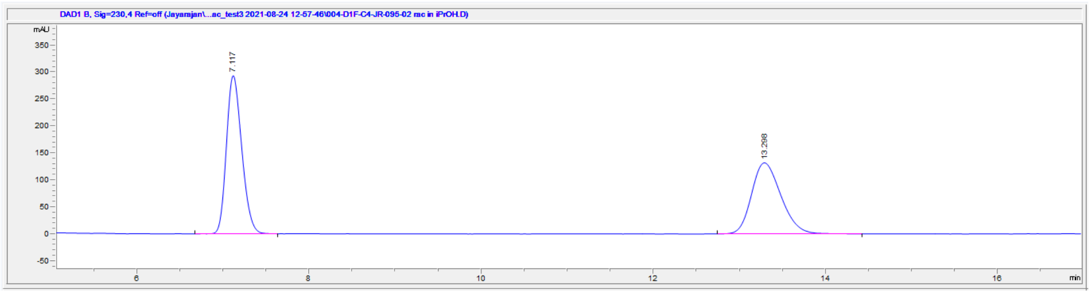

Signal 2: DAD1 B, Sig=230,4 Ref=off

Peak #	RetTime [min]	Type	Width [min]	Area [mAU*s]	Height [mAU]	Area %
1	7.117	BB	0.1927	3626.61694	292.45029	53.6640
2	13.298	BB	0.3679	3131.38428	131.47639	46.3360
Totals :				6758.00122	423.92668	

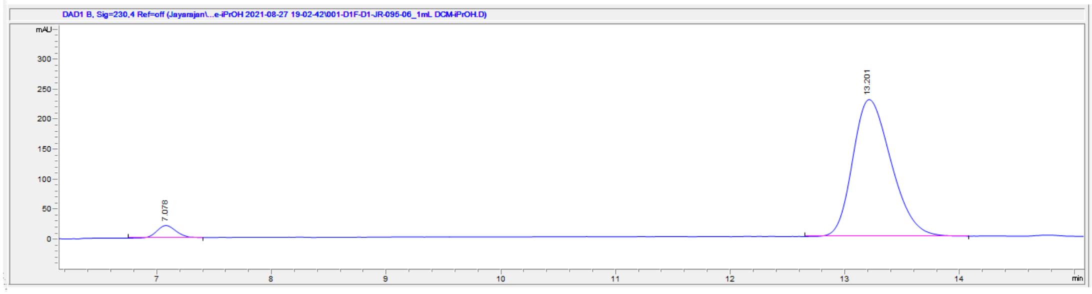

Signal 2: DAD1 B, Sig=230,4 Ref=off

Peak #	RetTime [min]	Type	Width [min]	Area [mAU*s]	Height [mAU]	Area %
1	7.078	BB	0.1838	245.56694	20.78687	4.4138
2	13.201	BB	0.3638	5318.00195	228.28757	95.5862
Totals :				5563.56889	249.07444	

(S)-2-(2,2,2-trifluoro-1-(furan-3-yl)ethyl)-2,3-dihydro-1H-naphtho[1,8-de][1,3,2]diazaborinine (5e):

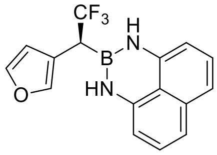

This compound was obtained according to the above general procedure A except that the reaction time was 48 h. Product **5e** was isolated in 48% yield (15.2 mg, 0.048 mmol) as viscous oil by silica gel chromatography using pentane/dichloromethane 4:1 solvent system as eluent.

^{1}H NMR (400 MHz, $CDCl_3$) δ 7.48 (t, *J* = 1.7 Hz, 1H), 7.45 (s, 1H), 7.13 – 7.05 (m, 4H), 6.42 (s, 1H), 6.32 (dd, *J* = 7.1, 1.2 Hz, 2H), 5.74 (s, 2H), 3.21 (q, *J* = 11.9 Hz, 1H). **^{13}C NMR** (101 MHz, $CDCl_3$) δ 144.0, 141.3, 140.1, 136.3, 127.7, 127.5 (q, *J* = 277.6 Hz), 119.9, 118.6, 116.8 (q, *J* = 3.5 Hz), 111.3, 106.6, 31.3. **^{19}F NMR** (377 MHz, $CDCl_3$) δ -61.87 (d, *J* = 11.9 Hz). **^{11}B NMR** (128 MHz, $CDCl_3$) δ 28.89. **HRMS** (pos. APCI) *m/z* calcd for $C_{16}H_{13}BF_3N_2O$: 317.1070 $[M+H]^+$; found: 317.1057. $[\alpha]_D^{26}$ –20.0 (*c* 0.25, $CHCl_3$).

Determination of *ee* by Chiral SFC: Daicel CHIRALPAK OJ-3, 25 °C, 0.46 cm *ϕ*, 25 cm column, 15% *i*PrOH in CO_2, flow rate: 1.2 mL/min; *t*R: 3.43 min (minor enantiomer), 3.88 min (major enantiomer); *ee* = 96%.

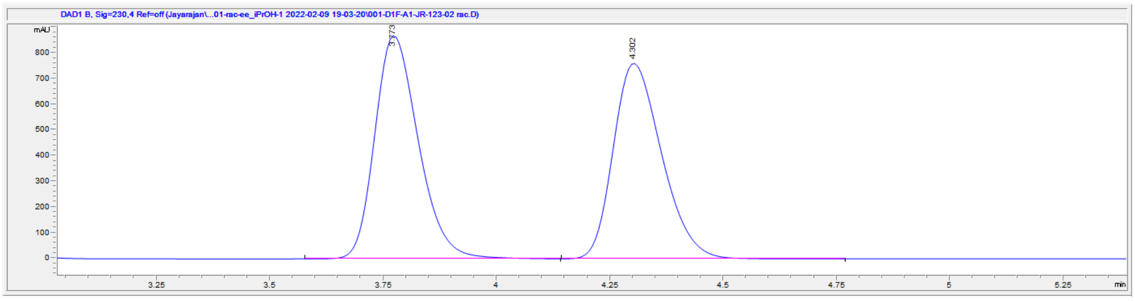

Signal 2: DAD1 B, Sig=230,4 Ref=off

Peak #	RetTime [min]	Type	Width [min]	Area [mAU*s]	Height [mAU]	Area %
1	3.773	BB	0.1018	5798.26563	869.56647	50.7104
2	4.302	BB	0.1144	5635.81738	760.90668	49.2896
Totals :				1.14341e4	1630.47314	

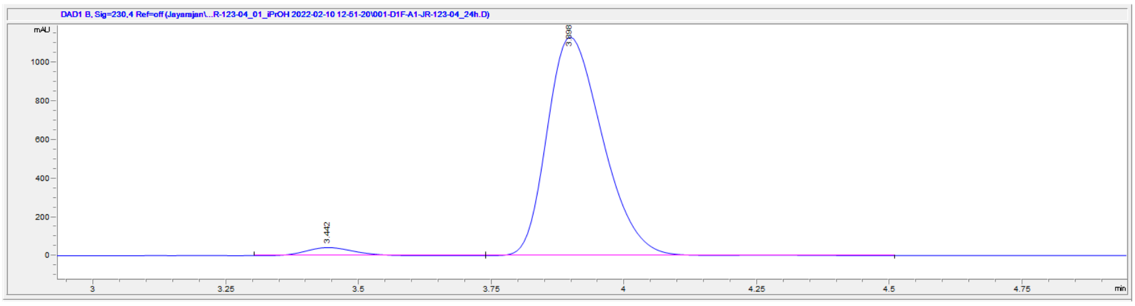

Signal 2: DAD1 B, Sig=230,4 Ref=off

Peak #	RetTime [min]	Type	Width [min]	Area [mAU*s]	Height [mAU]	Area %
1	3.432	BB	0.0974	235.42691	37.42129	2.2388
2	3.881	BB	0.1152	1.02802e4	1407.19031	97.7612
Totals :				1.05156e4	1444.61160	

(S)-2-(2,2,2-trifluoro-1-(thiophen-3-yl)ethyl)-2,3-dihydro-1H-naphtho[1,8-de][1,3,2]diazaborinine (5f):

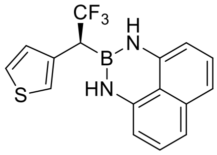

This compound was obtained according to the above general procedure A except that the reaction time was 48 h. Product **5f** was isolated in 92% yield (30.5 mg, 0.092 mmol) as white solid by silica gel chromatography using pentane/dichloromethane 4:1 solvent system as eluent; mp 107-111 °C.

^{1}H NMR (400 MHz, $CDCl_3$) δ 7.40 (dd, *J* = 5.0, 3.0 Hz, 1H), 7.24 (d, *J* = 2.0 Hz, 1H), 7.13 – 7.05 (m, 5H), 6.31 (dd, *J* = 7.0, 1.2 Hz, 2H), 5.70 (s, 2H), 3.44 (q, *J* = 12.1 Hz, 1H). **^{13}C NMR** (101 MHz, $CDCl_3$) δ 140.1, 136.3, 132.6 (t, *J* = 3.2 Hz), 128.6, 127.7, 127.5 (q, *J* = 277.7 Hz), 126.9, 124.2, 119.9, 118.6, 106.6, 36.7. **^{19}F NMR** (377 MHz, $CDCl_3$) δ -61.26 (d, *J* = 12.1 Hz). **^{11}B NMR** (128 MHz, $CDCl_3$) δ 28.94. **HRMS** (pos. APCI) *m/z* calcd for $C_{16}H_{13}BF_3N_2S$: 333.0842 $[M+H]^+$; found: 333.0842. $[\alpha]_D^{29}$ −35.2 (*c* 0.25, $CHCl_3$).

Determination of *ee* by Chiral SFC: Daicel CHIRALPAK OJ-3, 25 °C, 0.46 cm *ϕ*, 25 cm column, 15% *i*PrOH in CO_2, flow rate: 1.2 mL/min; *t*R: 5.67 min (minor enantiomer), 6.30 min (major enantiomer); *ee* = 94%.

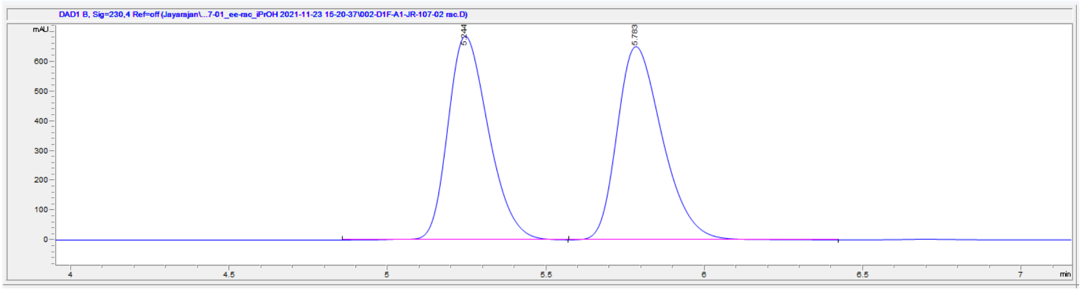

Signal 2: DAD1 B, Sig=230,4 Ref=off

Peak #	RetTime [min]	Type	Width [min]	Area [mAU*s]	Height [mAU]	Area %
1	5.244	BB	0.1379	6092.18848	684.12836	48.6288
2	5.783	BB	0.1537	6435.75244	648.73981	51.3712
Totals :				1.25279e4	1332.86816	

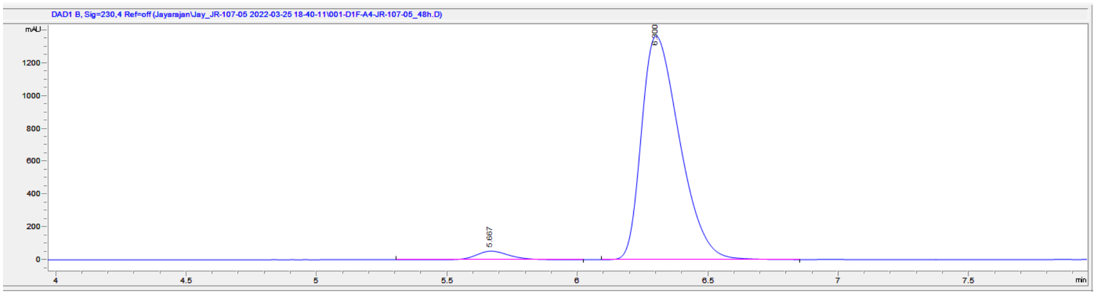

Signal 2: DAD1 B, Sig=230,4 Ref=off

Peak #	RetTime [min]	Type	Width [min]	Area [mAU*s]	Height [mAU]	Area %
1	5.667	BB	0.1346	459.25327	52.20573	3.2221
2	6.300	BB	0.1562	1.37938e4	1361.17871	96.7779
Totals :				1.42531e4	1413.38444	

(S)-2-(2,2,2-trifluoro-1-(1H-indol-5-yl)ethyl)-2,3-dihydro-1H-naphtho[1,8-de][1,3,2]diazaborinine (5g):

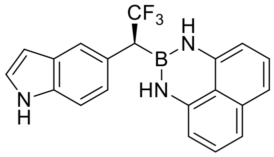

This compound was obtained according to the above general procedure A. Product **5g** was isolated in 68% yield (24. 8 mg, 0.068 mmol) as yellowish solid by silica gel chromatography using pentane/ethyl acetate 5:1 solvent system as eluent. mp = 156 - 162 °C.

^{1}H NMR (400 MHz, $CDCl_3$) δ 8.17 (s, 1H), 7.62 (s, 1H), 7.40 (d, *J* = 8.4 Hz, 1H), 7.25 – 7.24 (t, 1H), 7.15 (d, 1H), 7.12 – 7.04 (m, 4H), 6.58 – 6.57 (m, 1H), 6.28 (dd, *J* = 7.1, 1.1 Hz, 2H), 5.74 (s, 2H), 3.36 (q, *J* = 12.4 Hz, 1H). **^{13}C NMR** (101 MHz, $CDCl_3$) δ 140.4, 136.3, 135.4, 128.5, 128.2 (q, *J* = 277.9 Hz), 127.7, 125.2, 124.3 (q, *J* = 3.1 Hz), 124.1, 122.6, 119.8, 118.3, 111.8, 106.4, 102.8, 41.3. **^{19}F NMR** (377 MHz, $CDCl_3$) δ -60.70 (d, *J* = 12.4 Hz).**^{11}B NMR** (128 MHz, $CDCl_3$) δ 29.34. **HRMS** (pos. APCI) *m/z* calcd for $C_{20}H_{16}BF_3N_3$: 366.1387 $[M+H]^+$; found: 366.1374. $[\boldsymbol{\alpha}]_D^{27}$ –49.6 (*c* 0.25, $CHCl_3$).

Determination of *ee* by Chiral SFC: Daicel CHIRALPAK OJ-3, 25 °C, 0.46 cm ϕ, 25 cm column, 20% *i*PrOH in CO_2, flow rate: 1.2 mL/min; *t*R: 14.90 min (minor enantiomer), 25.57 min (major enantiomer); *ee* = 84%.

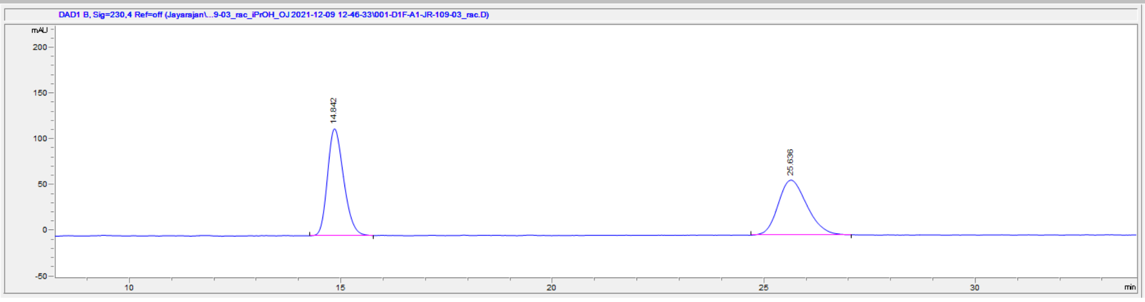

Signal 2: DAD1 B, Sig=230,4 Ref=off

Peak #	RetTime [min]	Type	Width [min]	Area [mAU*s]	Height [mAU]	Area %
1	14.842	BB	0.4024	3113.35059	117.03306	52.1901
2	25.636	BB	0.6584	2852.05420	60.23441	47.8099
Totals :				5965.40479	177.26747	

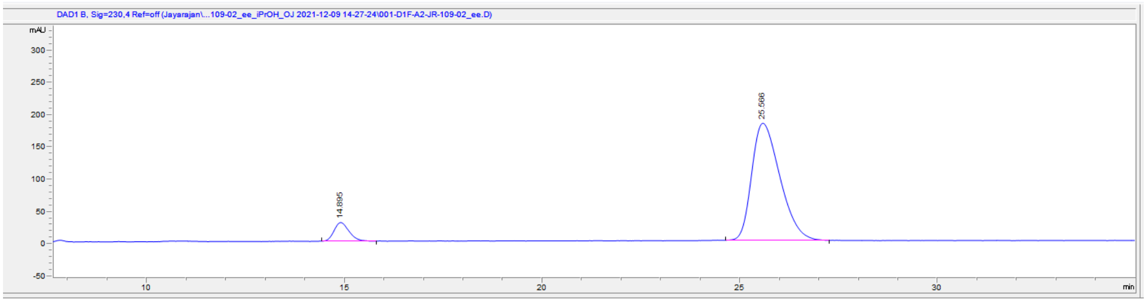

Signal 2: DAD1 B, Sig=230,4 Ref=off

Peak #	RetTime [min]	Type	Width [min]	Area [mAU*s]	Height [mAU]	Area %
1	14.895	BB	0.3978	759.04425	28.77012	7.7773
2	25.566	BB	0.7414	9000.70117	181.25148	92.2227
Totals :				9759.74542	210.02160	

(S)-2-(2,2,2-trifluoro-1-(1H-indol-6-yl)ethyl)-2,3-dihydro-1H-naphtho[1,8-de][1,3,2]diazaborinine (5h):

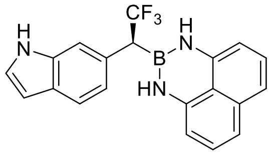

This compound was obtained according to the above general procedure A. Product **5a** was isolated in 80% yield (29.3 mg, 0.080 mmol) as yellowish solid by silica gel chromatography using pentane/ethyl acetate 5:1 solvent system as eluent; mp 150-156 °C.

^{1}H NMR (400 MHz, $CDCl_3$) δ 8.26 (s, 1H), 7.81 (d, *J* = 8.1 Hz, 1H), 7.49 (s, 1H), 7.38 – 7.36 (t, 1H), 7.26 – 7.19 (m, 5H), 6.73 (s, 1H), 6.41 (d, *J* = 7.0 Hz, 2H), 5.87 (s, 2H), 3.50 (q, *J* = 12.4 Hz, 1H). **^{13}C NMR** (101 MHz, $CDCl_3$) δ 140.3, 136.3, 136.2, 130.9 (d, *J* = 277.2 Hz), 127.7, 127.5, 126.9 – 126.1 (m), 125.1, 122.3, 121.5, 119.8, 118.4, 112.6, 106.5, 102.7.**^{19}F NMR** (377 MHz, $CDCl_3$) δ -60.48 (d, *J* = 12.4 Hz). **^{11}B NMR** (128 MHz, $CDCl_3$) δ 29.87. **HRMS** (pos. APCI) *m/z* calcd for $C_{20}H_{16}BF_3N_3$: 366.1387 $[M+H]^+$; found: 366.1391. $[\alpha]_D^{28}$ –21.6 (*c* 0.25, $CHCl_3$).

Determination of *ee* by Chiral SFC: Daicel CHIRALPAK OJ-3, 25 °C, 0.46 cm ϕ, 25 cm column, 20% *i*PrOH in CO_2, flow rate: 1.2 mL/min; *t*R: 15.18 min (minor enantiomer), 50.166 min (major enantiomer); *ee* = 85%.

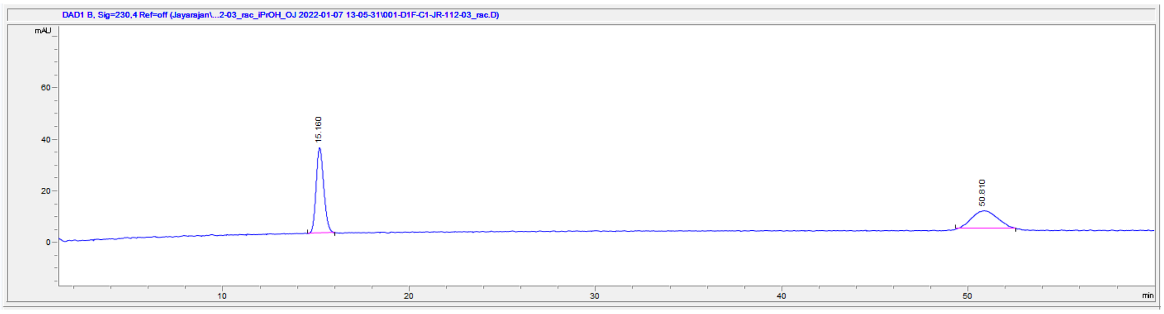

Signal 2: DAD1 B, Sig=230,4 Ref=off

Peak #	RetTime [min]	Type	Width [min]	Area [mAU*s]	Height [mAU]	Area %
1	15.160	BB	0.4096	952.88086	33.32740	57.8029
2	50.810	BB	1.1533	695.61957	7.11050	42.1971
Totals :				1648.50043	40.43791	

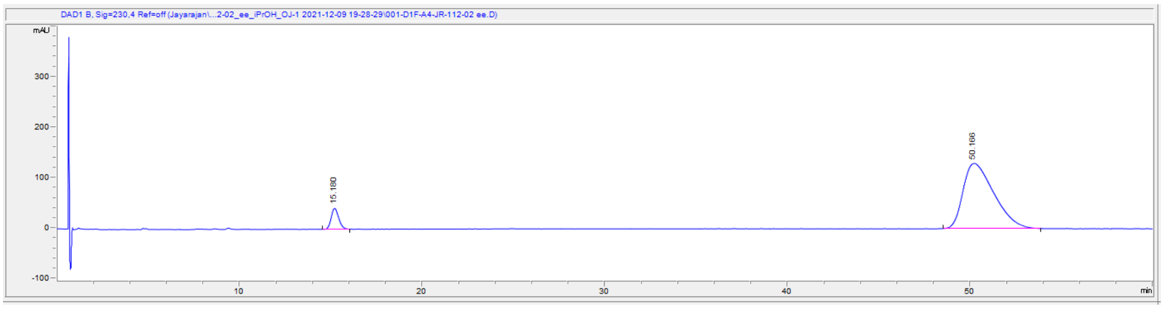

Signal 2: DAD1 B, Sig=230,4 Ref=off

Peak #	RetTime [min]	Type	Width [min]	Area [mAU*s]	Height [mAU]	Area %
1	15.180	BB	0.4247	1192.35938	41.56178	7.3277
2	50.166	BB	1.3782	1.50796e4	128.96387	92.6723
Totals :				1.62720e4	170.52565	

(S)-2-(1,1,1-trifluoro-4-phenylbutan-2-yl)-2,3-dihydro-1H-naphtho[1,8-de][1,3,2]diazaborinine (5i):

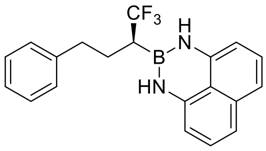

This compound was obtained according to the above general procedure B. Product **5i** was isolated in 73% yield (25.7 mg, 0.073 mmol) as colourless oil by silica gel chromatography using pentane/dichloromethane 5:1 solvent system as eluent.

^{1}H NMR (400 MHz, $CDCl_3$) δ 7.35 - 7.32 (m, 2H), 7.24 – 7.21 (m, 3H), 7.16 – 7.07 (m, 4H), 6.35 (d, J = 7.1 Hz, 2H), 5.68 (s, 2H), 2.97 – 2.79 (m, 1H), 2.77 – 2.69 (m, 1H), 2.25 – 2.16 (m, 1H), 2.01 – 1.92 (m, 1H), 1.90 – 1.78 (m, 1H). **^{13}C NMR** (101 MHz, $CDCl_3$) δ 140.8, 140.2, 136.3, 129.0 (q, J = 277.7 Hz), 128.8, 128.5, 127.7, 126.6, 120.0, 118.5, 106.4, 34.6, 32.9, 27.2 (q, J = 3.3 Hz). **^{19}F NMR** (377 MHz, $CDCl_3$) δ –62.39 (d, J = 11.3 Hz). **^{11}B NMR** (128 MHz, $CDCl_3$) δ 29.32. **HRMS** (pos. APCI) *m/z* calcd for $C_{20}H_{19}BF_3N_2$: 355.1591 $[M+H]^+$; found: 355.1586. $[\alpha]_D^{27}$ –56.4 (c 0.25, $CHCl_3$).

Determination of *ee* by Chiral SFC: Daicel CHIRALCEL OJ-3, 25 °C, 0.3 cm ϕ, 15 cm column, 6.2% MeOH in CO_2, flow rate: 0.8 mL/min; *t*R: 38.89 min (major enantiomer), 41.23 min (minor enantiomer); *ee* = 98%.

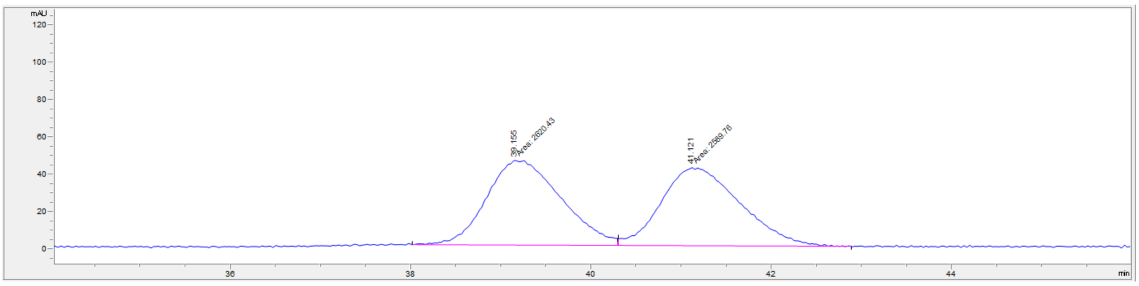

Signal 2: DAD1 B, Sig=230,4 Ref=off

Peak #	RetTime [min]	Type	Width [min]	Area [mAU*s]	Height [mAU]	Area %
1	39.155	MM	0.9629	2620.43213	45.35657	50.2943
2	41.121	MM	1.0255	2589.76001	42.08895	49.7057
Totals :				5210.19214	87.44552	

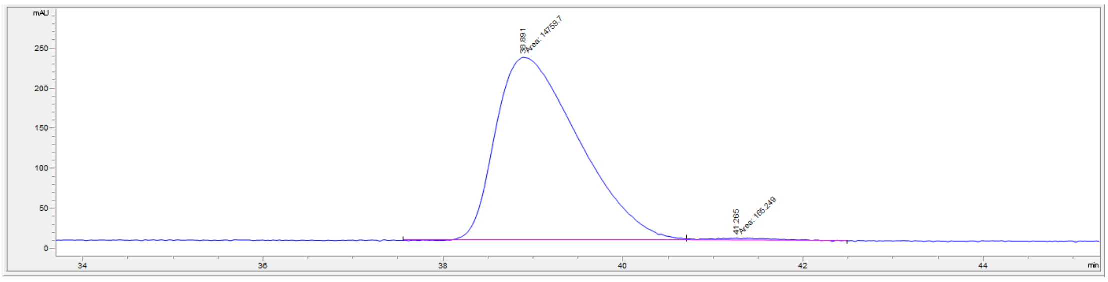

Signal 2: DAD1 B, Sig=230,4 Ref=off

Peak #	RetTime [min]	Type	Width [min]	Area [mAU*s]	Height [mAU]	Area %
1	38.891	MM	1.0756	1.47597e4	228.70052	98.8928
2	41.265	MM	0.9411	165.24881	2.92640	1.1072

(S)-2-(1-cyclopropyl-2,2,2-trifluoroethyl)-2,3-dihydro-1H-naphtho[1,8-de][1,3,2]diazaborinine (5j):

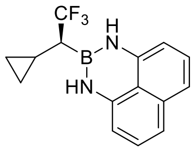

This compound was obtained according to the above general procedure B. Product **5j** was isolated in 61% yield (17.8 mg, 0.061 mmol) as white solid by silica gel chromatography using pentane/dichloromethane 5:1 solvent system as eluent; mp 107-110 °C.

^{1}H NMR (400 MHz, $CDCl_3$) δ 7.15 – 7.06 (m, 4H), 6.36 (d, J = 7.2 Hz, 2H), 5.88 (s, 2H), 1.15 (p, *J* = 11.2 Hz, 1H), 1.02 - 0.93 (m, 1H), 0.77 - 0.68 (m, *J* = 7.9 Hz, 2H), 0.48 - 0.44 (m, 1H), 0.25 - 0.18 (m, 1H). **^{13}C NMR** (101 MHz, $CDCl_3$) δ 140.4, 136.4, 129.08 (q, *J* = 278.6 Hz), 127.7, 120.0, 118.4, 106.4, 38.0, 7.2 (q, *J* = 4.2 Hz), 5.6, 3.6. **^{19}F NMR** (377 MHz, $CDCl_3$) δ –62.45 (d, *J* = 11.4 Hz). **^{11}B NMR** (128 MHz, $CDCl_3$) δ 29.61. After multiple attempts it was not possible to obtain HRMS data for this compound due to resistance to ionization. **GC-MS** m/z: 290 (M^+, 11), 186 (100), 185 (36), 166 (13), 140 (6). $[\alpha]_D^{24}$ –52.4 (*c* 0.25, $CHCl_3$).

Determination of *ee* by Chiral SFC: Daicel CHIRALPAK IA-3, 25 °C, 0.46 cm ϕ, 25 cm column, 5% MeOH in CO_2, flow rate: 2.0 mL/min; *t*R: 16.62 min (major enantiomer), 17.56 min (minor enantiomer); *ee* = 95%.

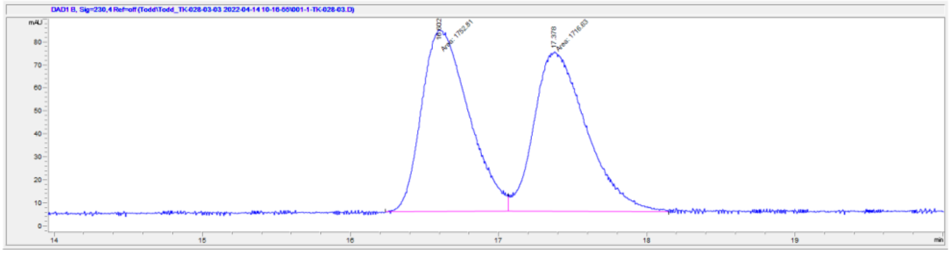

Signal 2: DAD1 B, Sig=230,4 Ref=off

Peak #	RetTime [min]	Type	Width [min]	Area [mAU*s]	Height [mAU]	Area %
1	16.602	MM	0.3702	1752.81213	78.92216	50.5214
2	17.378	MM	0.4118	1716.63367	69.48366	49.4786
Totals :				3469.44580	148.40582	

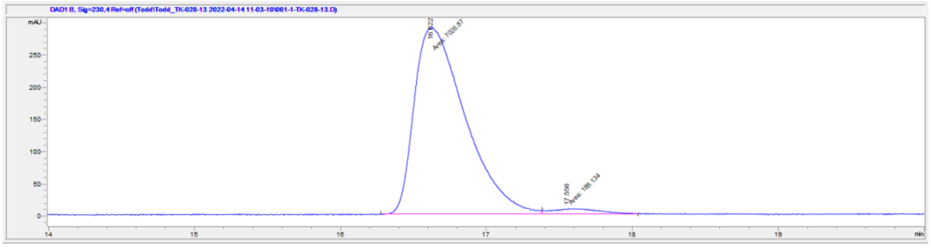

Signal 2: DAD1 B, Sig=230,4 Ref=off

Peak #	RetTime [min]	Type	Width [min]	Area [mAU*s]	Height [mAU]	Area %
1	16.622	MM	0.4052	7026.86670	289.05322	97.3925
2	17.556	MM	0.3558	188.13425	8.81172	2.6075
Totals :				7215.00095	297.86494	

Method for the crystal growth is given in page 11.
X-ray structure of 5j (CCDC number: 2173516):

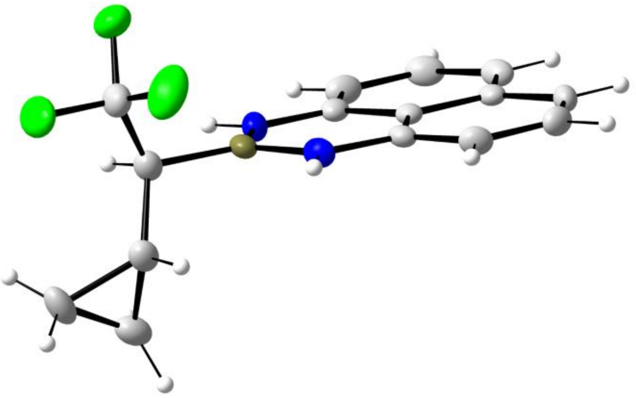

Table 2 Crystal data and structure refinement for compound 5j

Empirical formula	$C_{15}H_{14}BF_3N_2$
Formula weight	290.09
Temperature / K	100
Crystal system	monoclinic
Space group	$P2_1$ (nr. 4)
a / Å	10.3769(6)
b / Å	11.5454(6)
c / Å	11.3343(6)
α / °	90
β / °	93.8460(10)
γ / °	90
Volume / $Å^3$	1354.85(13)
Z	2 and Z' = 2 which means 4 unique molecules in cell.
ρ_{calc} / g cm^{-3}	1.422
μ / mm^{-1}	0.956
F(000)	600
Crystal size / mm^3	0.20 · 0.05 · 0.03
Radiation	CuKα (λ = 1.54184 Å)
2θ range for data collection / °	7.82 to 140.11
Index ranges	$-12 \leq h \leq 12$, $-14 \leq k \leq 14$, $-13 \leq l \leq 13$
Reflections collected	43193 [R_{int} = 0.0500, R_{sigma} = 0.0286]
Unique reflections	5120 (all data), 5067(I ≥ 2σ(I))
Data / restraints / parameters	5120, 1, 379
Goodness of fit on F^2	1.059
Final R indexes [I ≥ 2σ(I)]	R1 = 0.0370, wR2 = 0.0985
Final R indexes [all data]	R1 = 0.0373, wR2 = 0.0988
Largest diff peak and hole / e $Å^{-3}$	-0.234 … 0.399

(S)-2-(1,1,1,5,5,5-hexafluoropentan-2-yl)-2,3-dihydro-1H-naphtho[1,8-de][1,3,2]diazaborinine (5k):

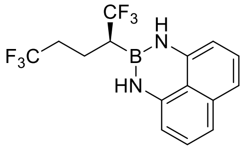

This compound was obtained according to the above general procedure B. Product **5k** was isolated in 82% yield (28.2 mg, 0.082 mmol) as colourless oil by silica gel chromatography using pentane/dichloromethane 4:1 solvent system as eluent. Traces of the Dan-protected starting material were present in the pure sample due to similar polarity to **5k**.

^{1}H NMR (400 MHz, $CDCl_3$) δ 7.17 – 7.09 (m, 4H), 6.38 (dd, J = 7.0, 1.3 Hz, 2H), 5.74 (s, 2H), 2.32 – 2.20 (m, 2H), 2.14 – 2.03 (m, 1H), 1.97 – 1.85 (m, 2H). **^{13}C NMR** (101 MHz, $CDCl_3$) δ 139.8, 136.3, 128.5 (q, J = 277.83 Hz), 127.7, 126.5 (q, J = 276.48 Hz), 120.1, 118.9, 106.7, 32.8 (q, J = 29.1 Hz), 18.7 (p, J = 3.6 Hz). **^{19}F NMR** (377 MHz, $CDCl_3$) δ -62.55 (d, J = 10.5 Hz), -66.39 (t, J = 10.5 Hz). **^{11}B NMR** (128 MHz, $CDCl_3$) δ 29.36. **HRMS** (pos. APCI) *m/z* calcd for $C_{15}H_{14}BF_6N_2$: 347.1151 [*M*+H]$^+$; found: 347.1151. $[\alpha]_D^{25}$ −2.0 (*c* 0.25, $CHCl_3$).

Determination of *ee* by Chiral SFC: Daicel CHIRALPAK IB N-3, 25 °C, 0.3 cm ϕ, 15 cm column, 3% iPrOH in CO_2, flow rate: 0.8 mL/min. Sample was prepared in iPrOH for SFC injection; *t*R: 9.24 min (minor enantiomer), 10.15 min (major enantiomer); *ee* = 96%.

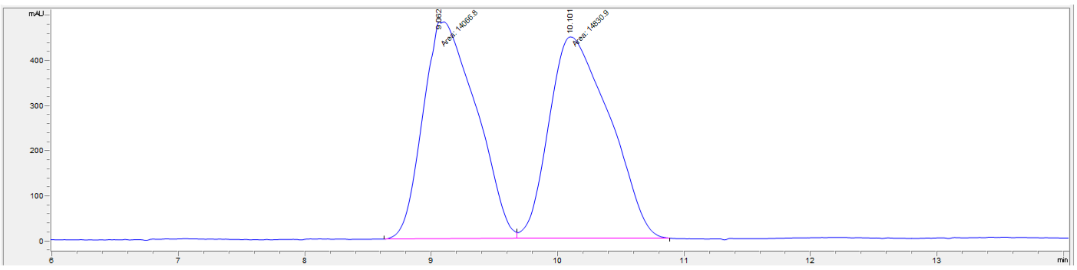

Signal 2: DAD1 B, Sig=230,4 Ref=off

Peak #	RetTime [min]	Type	Width [min]	Area [mAU*s]	Height [mAU]	Area %
1	9.062	MM	0.4888	1.40668e4	479.68018	48.6780
2	10.101	MM	0.5545	1.48309e4	445.76376	51.3220
Totals :				2.88977e4	925.44394	

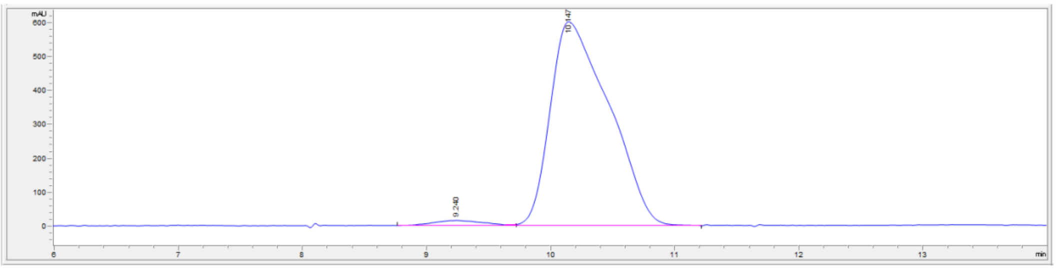

Signal 2: DAD1 B, Sig=230,4 Ref=off

Peak #	RetTime [min]	Type	Width [min]	Area [mAU*s]	Height [mAU]	Area %
1	9.240	BV E	0.3365	416.97714	15.08549	2.0644
2	10.147	VB R	0.4680	1.97811e4	600.92035	97.9356
Totals :				2.01980e4	616.00584	

(S)-2-(7-bromo-1,1,1-trifluoroheptan-2-yl)-2,3-dihydro-1H-naphtho[1,8-de][1,3,2]diazaborinine (5l):

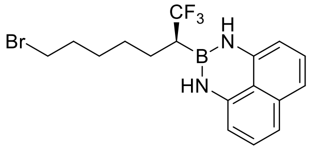

This compound was obtained according to the above general procedure B, except that dichloromethane was evaporated and pentane was added to the crude reaction mixture for the silica gel chromatography. Product **5l** was isolated in 52% yield (20.7 mg, 0.052 mmol) as colourless oil by silica gel chromatography using pentane/dichloromethane 5:1 solvent system as eluent.

^{1}H NMR (400 MHz, $CDCl_3$) δ 7.15 – 7.06 (m, 4H), 6.37 (d, *J* = 7.2 Hz, 2H), 5.74 (s, 2H), 3.40 (t, *J* = 6.6 Hz, 1H), 1.90 – 1.76 (m, 4H), 1.68 – 1.61 (m, 1H), 1.55 – 1.40 (m, 4H). **^{13}C NMR** (101 MHz, $CDCl_3$) δ 140.2, 136.3, 128.9 (q, J = 277.6 Hz), 127.5, 120.0, 118.5, 106.4, 33.9, 32.4, 28.2, 27.9, 25.8 (q, J = 3.3 Hz). **^{19}F NMR** (377 MHz, $CDCl_3$) δ -62.56 (d, *J* = 11.1 Hz). **^{11}B NMR** (128 MHz, $CDCl_3$) δ 29.67. **HRMS** (pos. APCI) *m/z* calcd for $C_{17}H_{20}BBrF_3N_2$: 399.0853 $[M+H]^+$; found: 399.0869. $[\alpha]_D^{26}$ −26.4 (*c* 0.25, $CHCl_3$).

Determination of *ee* by Chiral SFC: Daicel CHIRALPAK IB N-3, 25 °C, 0.3 cm ϕ, 15 cm column, 15% MeOH in CO_2, flow rate: 0.8 mL/min.; *t*R: 9.96 min (minor enantiomer), 11.17 min (major enantiomer); *ee* = 97%.

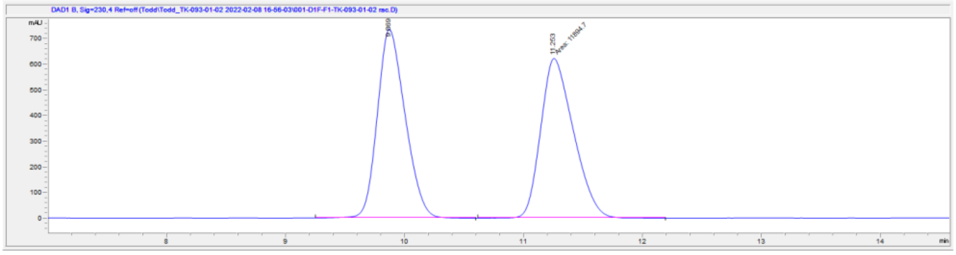

Signal 2: DAD1 B, Sig=230,4 Ref=off

Peak #	RetTime [min]	Type	Width [min]	Area [mAU*s]	Height [mAU]	Area %
1	9.869	BB	0.2467	1.19149e4	736.62738	50.0424
2	11.253	MM	0.3183	1.18947e4	622.85944	49.9576
Totals :				2.38097e4	1359.48682	

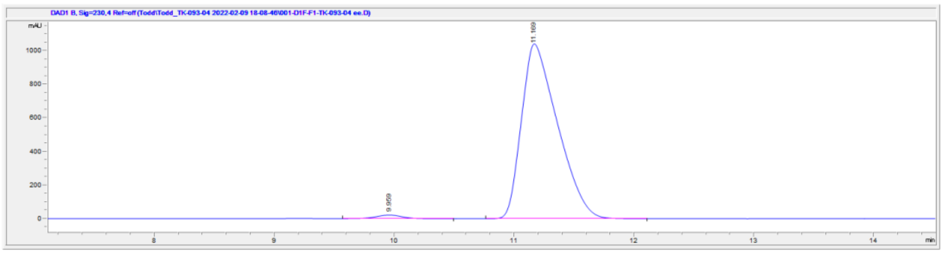

Signal 2: DAD1 B, Sig=230,4 Ref=off

Peak #	RetTime [min]	Type	Width [min]	Area [mAU*s]	Height [mAU]	Area %
1	9.959	BB	0.2292	323.58093	21.30427	1.4610
2	11.169	BB	0.3199	2.18249e4	1044.10669	98.5390
Totals :				2.21485e4	1065.41096	

(S)-2-(1,1,1-trifluorohexadecan-2-yl)-2,3-dihydro-1H-naphtho[1,8-de][1,3,2]diazaborinine (5m):

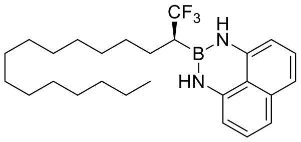

This compound was obtained according to the above general procedure B except that the reaction time was 54 h. Product **5m** was isolated in 62% yield (27.7 mg, 0.062 mmol) as colourless liquid by silica gel chromatography using pentane/dichloromethane 13:1 solvent system as eluent.

^{1}H NMR (400 MHz, $CDCl_3$) δ 7.15 – 7.06 (m, 4H), 6.36 (d, *J* = 7.2 Hz, 2H), 5.74 (s, 2H), 1.86 – 1.74 (m, 2H), 1.65 – 1.59 (m, 1H), 1.49 - 1.26 (m, 24 H), 0.89 (t, *J* = 6.7 Hz, 3H). **^{13}C NMR** (101 MHz, $CDCl_3$) δ 140.3, 136.3, 129.0 (q, J = 277.6 Hz), 127.7, 120.0, 118.4, 106.4, 34.0, 32.1, 29.84, 29.83, 29.81, 29.79, 29.78, 29.75, 29.7, 29.50, 29.46, 28.9, 25.9 (q, J = 3.3 Hz), 22.8, 14.3. **^{19}F NMR** (377 MHz, $CDCl_3$) δ -62.58 (d, *J* = 11.1 Hz). **^{11}B NMR** (128 MHz, $CDCl_3$) δ 30.04. **HRMS** (pos. APCI) *m/z* calcd for $C_{26}H_{39}BF_3N_2$: 447.3158 $[M+H]^+$; found: 447.3154. $[\alpha]_D^{25}$ –20.4 (*c* 0.25, $CHCl_3$).

Determination of *ee* by Chiral SFC: Daicel CHIRALPAK IB N-3, 25 °C, 0.3 cm ϕ, 15 cm column, 10% MeOH in CO_2, flow rate: 0.8 mL/min; *t*R: 13.48 min (minor enantiomer), 14.86 min (major enantiomer); *ee* = 97%.

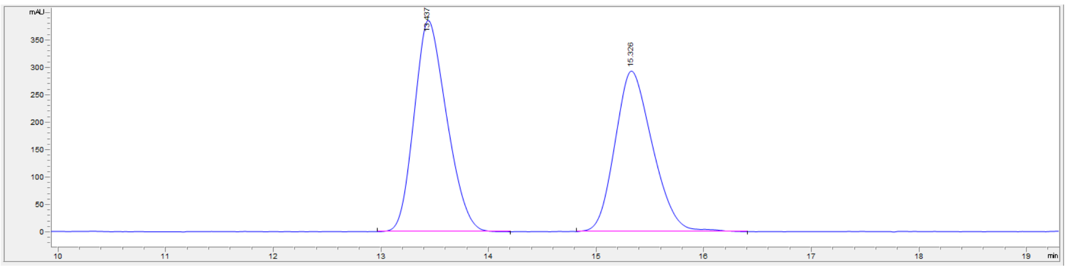

Signal 2: DAD1 B, Sig=230,4 Ref=off

Peak #	RetTime [min]	Type	Width [min]	Area [mAU*s]	Height [mAU]	Area %
1	13.437	BB	0.3214	8111.24756	385.66913	53.3249
2	15.326	BV R	0.3689	7099.73730	292.74622	46.6751
Totals :				1.52110e4	678.41534	

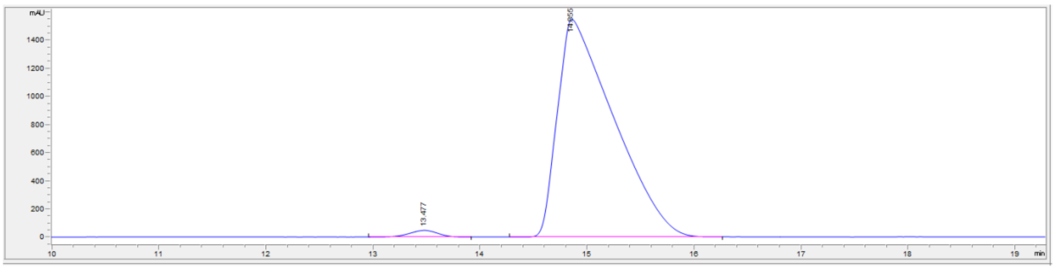

Signal 2: DAD1 B, Sig=230,4 Ref=off

Peak #	RetTime [min]	Type	Width [min]	Area [mAU*s]	Height [mAU]	Area %
1	13.477	BB	0.2928	870.40936	46.84511	1.5200
2	14.855	VV R	0.5129	5.63931e4	1541.19067	98.4800
Totals :				5.72635e4	1588.03578	

(S)-4,4,5,5-tetramethyl-2-(2,2,2-trifluoro-1-phenylethyl)-1,3,2-dioxaborolane (6):

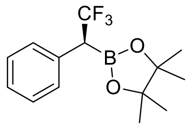

This compound was obtained according to the above general procedure A except that after 72 h, pinacol was used instead of DanH. Product **6** was isolated in 56% yield (16.1 mg, 0.056 mmol) as colorless oil by silica gel chromatography using pentane/DCM 4:1 as eluent.

^{1}H NMR (400 MHz, $CDCl_3$) δ 7.40 – 7.28 (m, 6H), 3.20 (q, *J* = 11.5 Hz, 1H), 1.26 (d, *J* = 14.9 Hz, 12H). **^{13}C NMR** (101 MHz, $CDCl_3$) δ 132.3 (q, *J* = 3.0 Hz), 130.4, 128.8, 127.7, 126.9 (q, *J* = 277.6 Hz), 84.7, 38.7, 24.7, 24.6. **^{19}F NMR** (377 MHz, $CDCl_3$) δ -62.71 (d, *J* = 11.5 Hz). **^{11}B NMR** (128 MHz, $CDCl_3$) δ 31.15. **HRMS** (pos. ESI) *m/z* calcd for $C_{14}H_{18}BF_3O_2Na$: 309.1247 [*M*+Na]$^+$; found: 309.1247.

(3aS,4S,6S,7aR)-3a,5,5-trimethyl-2-((S)-2,2,2-trifluoro-1-phenylethyl)hexahydro-4,6-methanobenzo[d][1,3,2]dioxaborole (7):

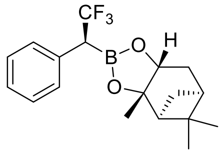

This compound was obtained according to the above general procedure A except that after 72 h, (+)-pinanediol (0.15 mmol) was used instead of DanH. Product **7** was isolated in 80% yield (27.2 mg, 0.080 mmol) as viscous oil by silica gel chromatography using pentane/DCM 4:1 as eluent.

^{1}H NMR (400 MHz, $CDCl_3$) δ 7.41 – 7.27 (m, 5H), 4.38 (dd, *J* = 8.8, 2.0 Hz, 1H), 3.26 (q, *J* = 11.6 Hz, 1H), 2.34 (ddt, *J* = 13.9, 8.9, 2.4 Hz, 1H), 2.23 (dtd, *J* = 11.1, 6.1, 2.3 Hz, 1H), 2.17 – 2.05 (m, 1H), 1.91 (tt, *J* = 5.8, 3.0 Hz, 1H), 1.85 (ddd, *J* = 14.6, 3.2, 2.1 Hz, 1H), 1.38 (s, 3H), 1.29 (s, 3H), 1.09 (d, *J* = 11.1 Hz, 1H), 0.84 (s, 3H). **^{13}C NMR** (101 MHz, $CDCl_3$) δ 132.4 (q, *J* = 3.2 Hz), 130.4, 128.8, 127.7, 127.0 (q, *J* = 277.7 Hz), 87.1, 78.7, 51.2, 39.5, 38.3, 35.4, 28.5, 27.1, 26.5, 24.1. **^{19}F NMR** (377 MHz, $CDCl_3$) δ -62.50 (d, *J* = 11.6 Hz). **^{11}B NMR** (128 MHz, $CDCl_3$) δ 30.53. **HRMS** (pos. ESI) *m/z* calcd for $C_{18}H_{22}BF_3O_2Na$: 361.1560 [*M*+Na]$^+$; found: 361.1554.
The diasteromeric ratio of the compound **7** was 95:5 determined on the basis of ^{19}F NMR spectrum.

(S)-2,2,2-trifluoro-1-(4-methoxyphenyl)ethan-1-ol (8a):

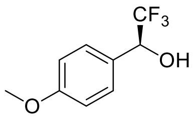

This compound was obtained according to the above general procedure C. Product **8a** was isolated in 85% yield (17.5 mg, 0.085 mmol) as colourless oil by silica gel chromatography using pentane/dichloromethane 2:1 to 1:1 solvent system as eluent. The spectral data are in line with the literature values.[4]

^{1}H NMR (400 MHz, $CDCl_3$) δ 7.40 (d, J = 8.5 Hz, 2H), 6.93 (d, J = 8.6 Hz, 2H), 5.00 – 4.94 (m, 1H), 3.83 (s, 3H), 2.50 (d, J = 4.4 Hz, 1H). **^{13}C NMR** (101 MHz, $CDCl_3$) δ 160.7, 128.9, 126.2, 124.5 (q, J = 281.9 Hz), 114.2, 72.7 (q, J = 32.1 Hz), 55.5. **^{19}F NMR** (377 MHz, $CDCl_3$) δ -78.56 (d, J = 6.7 Hz). **GC-MS** m/z: 206 (M^+, 11), 137 (100), 109 (56), 94 (60), 77 (64), 69 (40). $[\alpha]_D^{26}$ +26.0 (c 0.25, $CHCl_3$).

Determination of *ee* by Chiral SFC: Daicel CHIRALPAK OJ-3, 25 °C, 0.46 cm ϕ, 25 cm column, 2% *i*PrOH in CO_2, flow rate: 1.2 mL/min; *t*R: 4.95 min (minor enantiomer), 6.02 min (major enantiomer); *ee* = 88%.

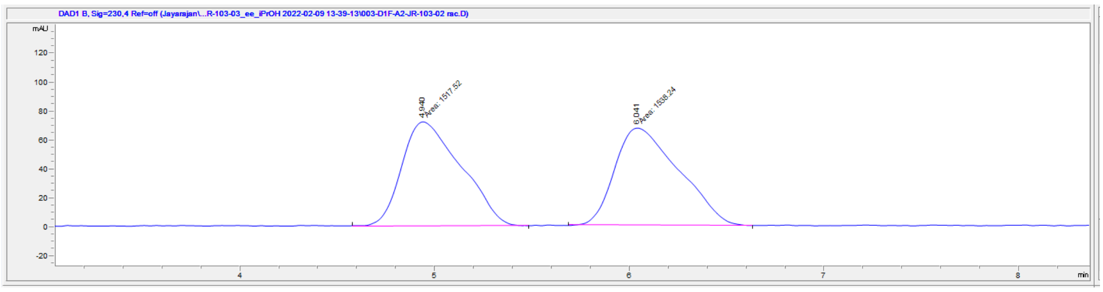

Signal 2: DAD1 B, Sig=230,4 Ref=off

Peak #	RetTime [min]	Type	Width [min]	Area [mAU*s]	Height [mAU]	Area %
1	4.940	MM	0.3522	1517.51672	71.81053	49.6609
2	6.041	MM	0.3825	1538.24292	67.02351	50.3391
Totals :				3055.75964	138.83405	

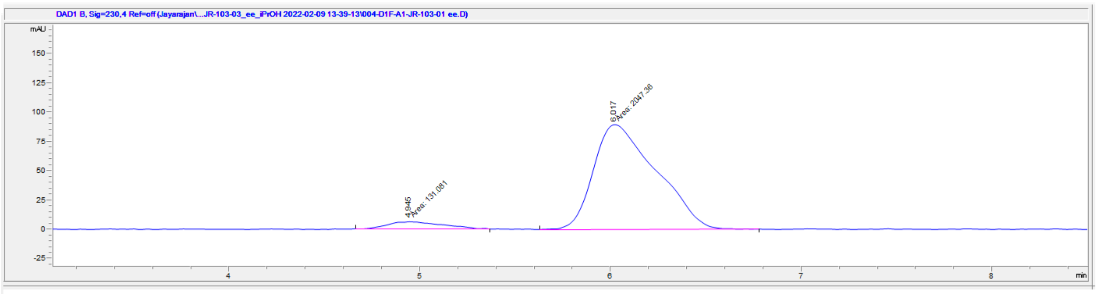

Signal 2: DAD1 B, Sig=230,4 Ref=off

Peak #	RetTime [min]	Type	Width [min]	Area [mAU*s]	Height [mAU]	Area %
1	4.945	MM	0.3442	131.08057	6.34700	6.0172
2	6.017	MM	0.3816	2047.35706	89.41631	93.9828
Totals :				2178.43762	95.76331	

(S)-2,2,2-trifluoro-1-(naphthalen-2-yl)ethan-1-ol (8b):

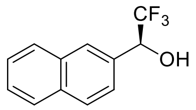

This compound was obtained according to the above general procedure C. Product **8b** was isolated in 92% yield (20.8 mg, 0.092 mmol) as colourless oil by silica gel chromatography using pentane/dichloromethane 2:1 to 1:1 solvent system as eluent. The spectral data are in line with the literature values.[4]

^{1}H NMR (400 MHz, $CDCl_3$) δ 7.95 (s, 1H), 7.93 – 7.81 (m, 3H), 7.63 – 7.50 (m, 3H), 5.92 – 4.48 (m, 1H), 2.79 (d, J = 4.6 Hz, 1H). **^{13}C NMR** (101 MHz, $CDCl_3$) δ 133.9, 133.0, 131.4, 128.7, 128.4, 127.9, 127.5, 127.0, 126.7, 124.5 (q, J = 282.2 Hz), 124.5, 73.2 (q, J = 32.0 Hz). **^{19}F NMR** (377 MHz, $CDCl_3$) δ -77.99 (d, J = 6.7 Hz). **GC-MS** m/z: 226 (M^+, 11), 157 (29), 129 (100), 128 (62), 102 (9). $[\alpha]_D^{27}$ +23.2 (*c* 0.5, $CHCl_3$).

Determination of *ee* by Chiral SFC: Daicel CHIRALPAK OJ-3, 25 °C, 0.46 cm ϕ, 25 cm column, 10% *i*PrOH in CO_2, flow rate: 1.2 mL/min; *t*R: 3.03 min (minor enantiomer), 5.52 min (major enantiomer); *ee* = 84%.

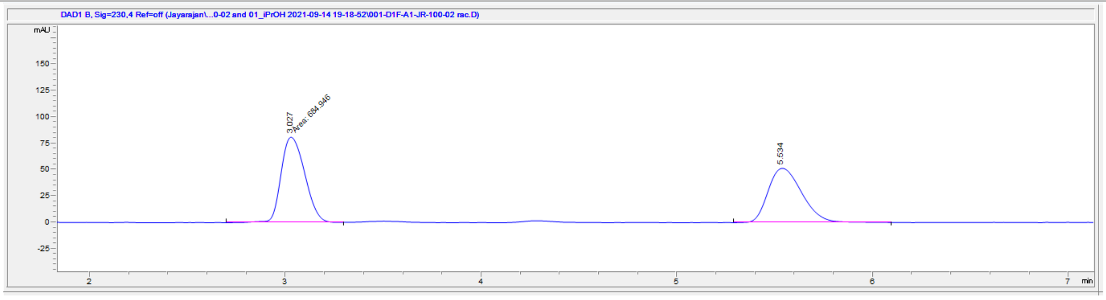

Signal 2: DAD1 B, Sig=230,4 Ref=off

Peak #	RetTime [min]	Type	Width [min]	Area [mAU*s]	Height [mAU]	Area %
1	3.027	MF	0.1409	684.94629	81.04879	52.7142
2	5.534	BB	0.1913	614.41290	51.44877	47.2858
Totals :				1299.35919	132.49756	

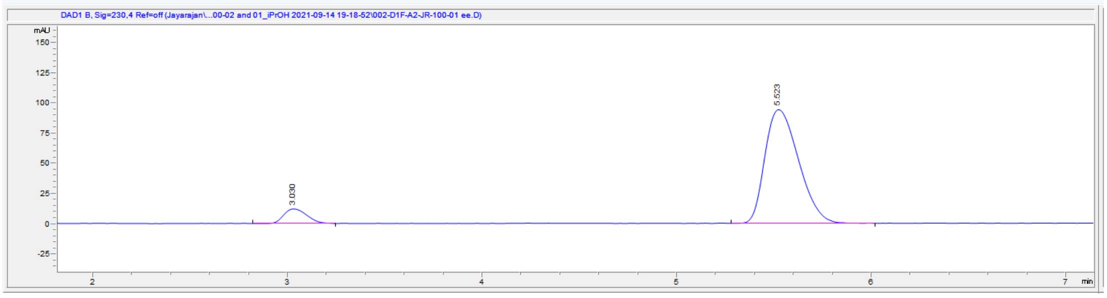

Signal 2: DAD1 B, Sig=230,4 Ref=off

Peak #	RetTime [min]	Type	Width [min]	Area [mAU*s]	Height [mAU]	Area %
1	3.030	BB	0.1353	100.86756	12.09640	8.1659
2	5.523	BV R	0.1907	1134.35840	94.03508	91.8341
Totals :				1235.22596	106.13148	

(S)-1,1,1-trifluorohexadecan-2-ol (8c):

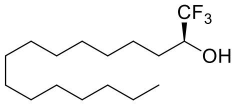

General procedure B was used for the homologation except that the reaction time was 54 h. Then, the dichloromethane solvent was reduced to 0.1 mL by argon blow and tetrahydrofuran (0.4 mL) solvent was added. Subsequently, a premixed 3M NaOH (0.3 mmol, 0.1 mL) and H_2O_2 (0.5 mmol, 0.1 mL, 35 wt. % in H_2O) solution was added at 0 °C. After 6 h of reaction time, another portion of hydrogen peroxide (0.3 mL) was added and stirred for 18 h. The reaction mixture was acidified with 1M HCl and 3 mL water was added. The organic layer was extracted with diethyl ether and the aqueous layer was washed 3 times with diethyl ether. The combined organic phase was passed through phase separator and the solvent was evaporated under reduced pressure. Product **8c** was isolated in 76% yield (22.4 mg, 0.076 mmol) as pale-yellow solid by silica gel chromatography using pentane/ethyl acetate 30:1 solvent system as eluent; mp 41 – 43 °C.

^{1}H NMR (400 MHz, $CDCl_3$) δ 3.91 (dtd, *J* = 9.9, 6.6, 3.4 Hz, 1H), 2.05 (d, *J* = 6.0 Hz, 1H), 1.74 – 1.67 (m, 1H), 1.63 – 1.54 (m, 2H), 1.42 – 1.26 (m, 23H), 0.88 (t, *J* = 6.7 Hz, 3H). **^{13}C NMR** (101 MHz, $CDCl_3$) δ 125.3 (q, *J* = 281.8 Hz), 70.7 (q, *J* = 30.9 Hz), 32.0, 29.8, 29.8, 29.8, 29.7, 29.7, 29.7, 29.6, 29.5, 29.5, 29.3, 25.0, 22.8, 14.2. **^{19}F NMR** (377 MHz, $CDCl_3$) δ -80.09 (d, *J* = 6.6 Hz). After multiple attempts it was not possible to obtain proper HRMS data for this compound. **GC-MS** m/z: 275 (44), 225 (46), 197 (96), 183 (64), 147 (100). $[\alpha]_D^{25}$ −14.8 (*c* 0.25, $CHCl_3$).

We were not able not obtain useful spectral data (by SFC / GC / HPLC) for the enantiopurity of **8c**. Thus, the ee of the product **8c** was determined on the basis of the ^{19}F NMR spectrum of the (S)-camphorsulfonyl ester derivative **8c-1**.

(S)-1,1,1-trifluorohexadecan-2-yl((1S,4R)-7,7-dimethyl-2-oxobicyclo[2.2.1]heptan-1-yl)methanesulfonate (8c-1):

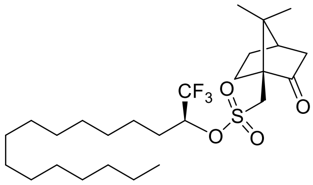

In a reaction tube, compound **8c** (14.5 mg, 0.049 mmol) and DMAP (1.2 mg, 0.01 mmol) were dissolved in dichloromethane (0.8 mL). Then, triethylamine (8 µL, 0.059 mmol) and S-camphorsulfonyl chloride (24.5 mg, 0.098 mmol) were added. The reaction mixture was stirred at RT for 18 h. Product **8c-1** was isolated in 89 % yield (22.2 mg, 0.044 mmol) as colourless oil by silica gel chromatography using pentane/ethyl acetate 30:1 solvent system as eluent.

^{1}H NMR (400 MHz, $CDCl_3$) δ 4.92 (h, *J* = 6.3 Hz, 1H), 3.74 (d, *J* = 15.0 Hz, 1H), 3.11 (d, *J* = 15.0 Hz, 1H), 2.50 – 2.38 (m, 2H), 2.14 – 2.08 (m, 2H), 1.96 (d, *J* = 18.5 Hz, 1H), 1.84 – 1.79 (m, 2H), 1.67 (ddd, *J* = 13.9, 9.4, 4.6 Hz, 1H), 1.60 – 1.51 (m, 1H), 1.45 (ddd, *J* = 13.1, 9.3, 3.9 Hz, 2H), 1.37 - 1.25(m, 22H), 1.14 (s, 3H), 0.90 – 0.86 (m, 6H). **^{13}C NMR** (101 MHz, $CDCl_3$) δ 213.9, 123.3 (q, *J* = 281.1 Hz), 76.8 (q, *J* = 32.7 Hz), 58.2, 49.2, 47.9, 43.0, 42.6, 32.1, 29.83, 29.81, 29.79, 29.76, 29.71, 29.6, 29.5, 29.3, 29.1, 28.6, 26.1, 25.3, 24.5, 22.8, 20.1, 19.8, 14.3. **^{19}F NMR** (377 MHz, $CDCl_3$) δ -76.42 (d, *J* = 6.2 Hz). **HRMS** (pos. ESI) *m/z* calcd for $C_{26}H_{45}F_3O_4SNa$: 533.2883 $[M+Na]^+$; found: 533.2882. $[\alpha]_D^{26}$ +18.8 (*c* 0.25, $CHCl_3$). Diastereomeric ratio of compound **8c-1** was 99:1 based on the ^{19}F NMR spectral data.

Ethyl (S)-4,4,4-trifluoro-3-(4-methoxyphenyl)butanoate (9a):

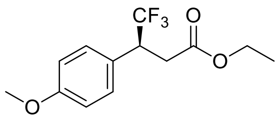

This compound was obtained according to the above general procedure D. Product **9a** was isolated in 58% yield (15.9 mg, 0.058 mmol) by silica gel chromatography using gradient of petroleum ether/Et_2O (100/0 to 90/10) to provide the compound as a colourless oil. The spectral data are in line with the literature values.[5]

^{1}H NMR (400 MHz, $CDCl_3$) δ 7.24 (d, *J* = 8.7 Hz, 2H), 6.90 – 6.86 (m, 2H), 4.10 – 4.01 (m, 2H), 3.91 – 3.83 (m, 1H), 3.80 (s, 3H), 2.99 (dd, *J* = 16.1, 5.0 Hz, 1H), 2.84 (dd, *J* = 16.1, 10.0 Hz, 1H), 1.14 (t, *J* = 7.1 Hz, 3H). **^{13}C NMR** (101 MHz, $CDCl_3$) δ 170.2, 159.8, 130.2, δ 126.6 (q, *J* = 279.6 Hz), 125.8 (q), 114.2, 61.1, 55.4, 45.5 (q, *J* = 27.8 Hz), 34.7 (d, *J* = 2.2 Hz), 14.2. **^{19}F NMR** (377 MHz, $CDCl_3$) δ -70.68. (d, *J* = 9.3 Hz). **GC-MS** m/z: 276 (M^+, 35), 256 (33), 228 (85), 189 (92), 184 (100). $[\alpha]_D^{26}$ +16.8 (*c* 0.25, $CHCl_3$).

Determination of *ee* by Chiral GC: Column Chiraldex β-DM column, 50 to 170 °C at 1 °C /min), *t*R = 64.67 min (major enantiomer), 63.93 (minor enantiomer); ee = 84%.

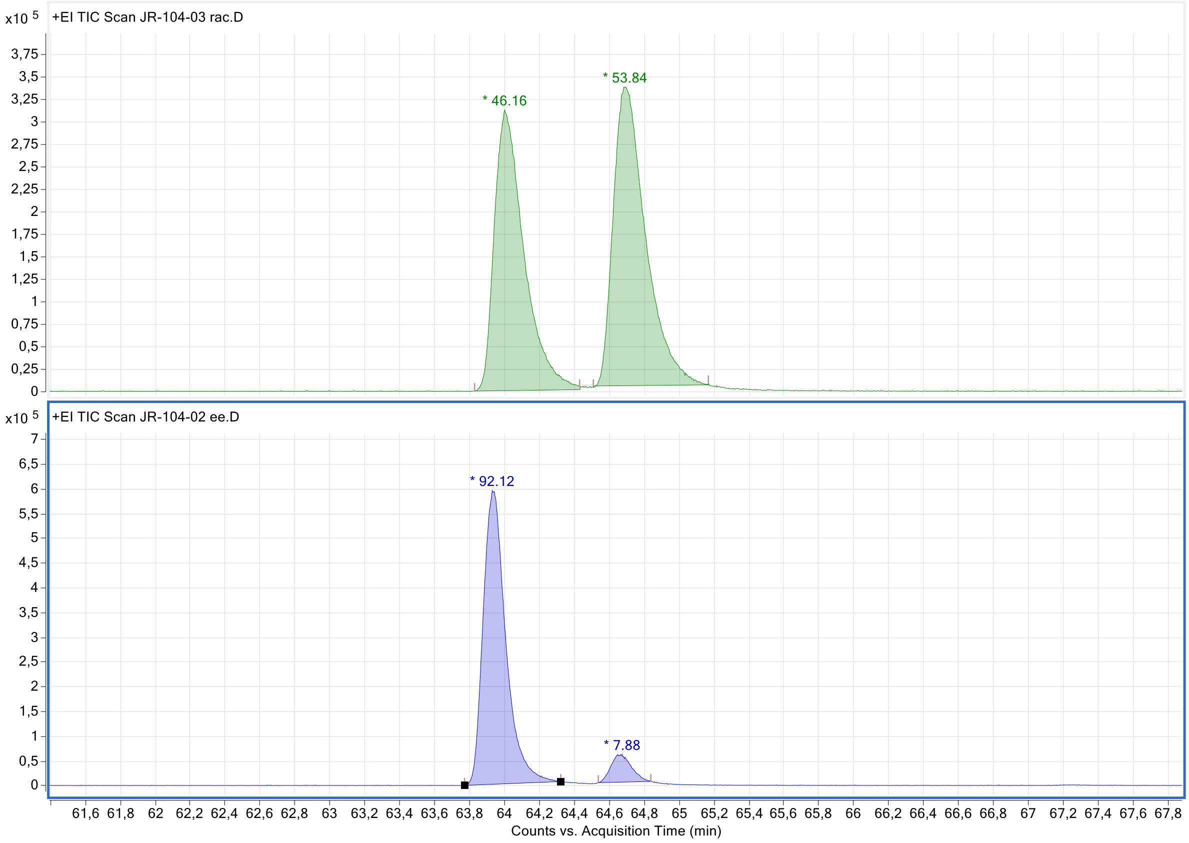

Ethyl (S)-4,4,4-trifluoro-3-(4-fluorophenyl)butanoate (9b):

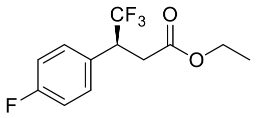

This compound was obtained according to the above general procedure D. Product **9b** was isolated in 51% yield (13.5 mg, 0.051 mmol) as by silica gel chromatography using gradient of petroleum ether/Et_2O (100/0 to 90/10) to provide the compound as a colourless oil. The spectral data are in line with the literature values.[5]

^{1}H NMR (400 MHz, $CDCl_3$) δ 7.31 (dd, *J* = 8.6, 5.3 Hz, 2H), 7.08 – 7.02 (m, 2H), 4.10 – 4.01 (m, 2H), 3.95 – 3.85 (m, 1H), 3.01 (dd, *J* = 16.2, 4.9 Hz, 1H), 2.85 (dd, *J* = 16.2, 10.1 Hz, 1H), 1.14 (t, *J* = 7.1 Hz, 3H). **^{13}C NMR** (126 MHz, $CDCl_3$) δ 170.0, 162.9 (d, *J* = 247.6 Hz), 130.8 (d, *J* = 8.3 Hz), 129.7 (dd, J = 3.6, 2.1 Hz), 126.4 (d, *J* = 279.6 Hz), 115.9 (d, *J* = 21.6 Hz), 61.3, 45.6 (q, *J* = 28.0 Hz), 34.7 (d, *J* = 2.1 Hz), 14.1. **^{19}F NMR** (377 MHz, $CDCl_3$) δ -70.58 (d, *J* = 9.2 Hz), -107.65 – -116.46 (m). **GC-MS** m/z: 244 (15), 216 (20), 188 (29), 172 (53), 127 (100), 121 (58), $[\alpha]_D^{26}$ +16.4 (*c* 0.25, $CHCl_3$).

Determination of *ee* by Chiral GC: Column Chiraldex β-DM column, 50 to 170 °C at 1 °C /min), *t*R = 36.89 min (major enantiomer), 38.23 (minor enantiomer); ee = 89%.

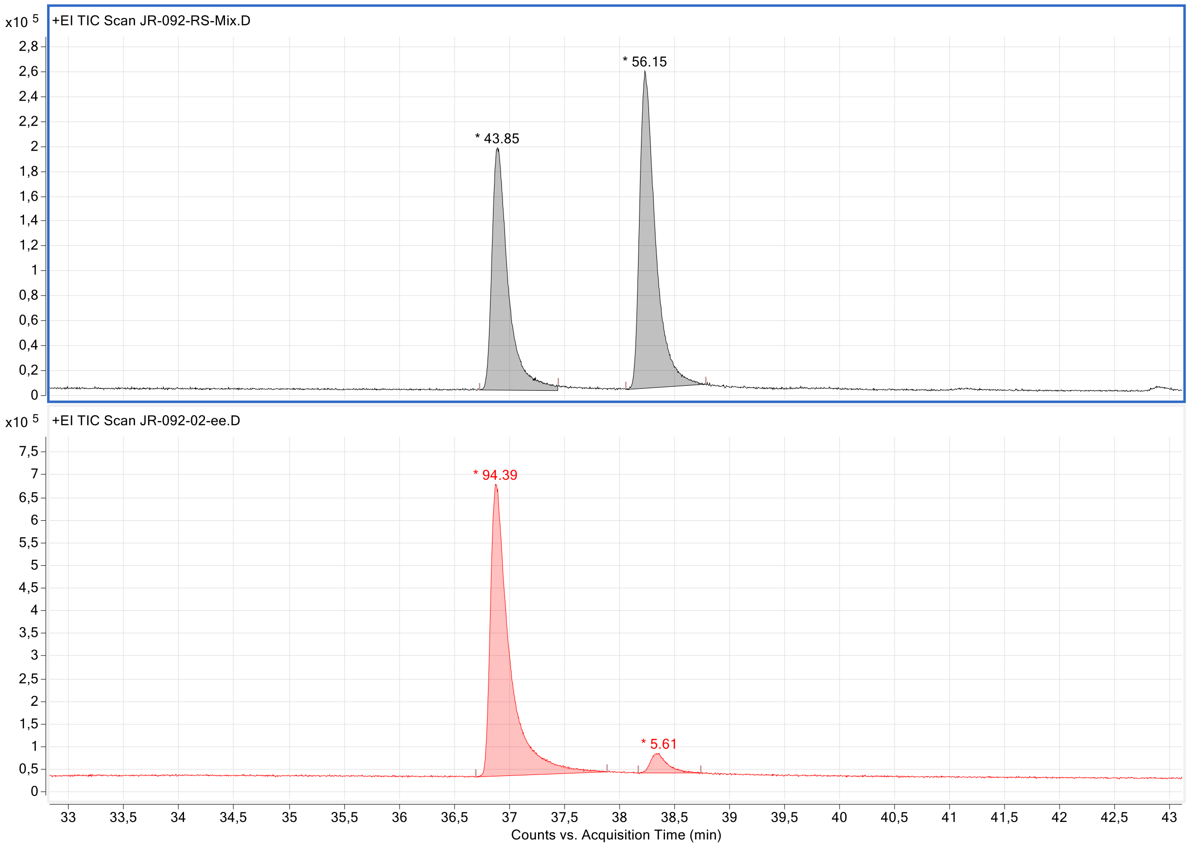

Ethyl (S)-4,4,4-trifluoro-3-(naphthalen-2-yl)butanoate (9c):

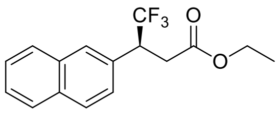

This compound was obtained according to the above general procedure A. Product **9c** was isolated in 68% yield (20.0 mg, 0.068 mmol) as by silica gel chromatography using gradient of pentane/ethyl acetate (100/0 to 98/2) to provide the compound as a colourless oil. The spectral data are in line with the literature values.[5]

^{1}H NMR (400 MHz, $CDCl_3$) δ 7.86 – 7.81 (m, 4H), 7.50 (dd, *J* = 6.2, 3.2 Hz, 2H), 7.44 (d, *J* = 8.5 Hz, 1H), 4.13 – 3.96 (m, 3H), 3.11 (dd, *J* = 16.2, 5.1 Hz, 1H), 3.01 (dd, *J* = 16.2, 9.8 Hz, 1H), 1.11 (t, *J* = 7.1 Hz, 3H). **^{13}C NMR** (101 MHz, $CDCl_3$) δ 170.1, 133.3, 131.1 (d, *J* = 51.1 Hz), 128.7, 128.2, 127.8, 126.6, 126.6 (q, *J* = 279.8 Hz), 126.6, 126.4, 61.2, 46.4 (q, *J* = 27.8 Hz), 34.7 (d, *J* = 2.2 Hz), 14.1. **^{19}F NMR** (377 MHz, $CDCl_3$) δ -69.99 (d, *J* = 9.2 Hz). **GC-MS** m/z: 296 (M^+, 33), 251 (27), 248 (100), 204 (85), 183 (66), 75 (22). $[\alpha]_D^{26}$ +24.8 (*c* 0.25, $CHCl_3$).

Determination of *ee* by Chiral SFC: Daicel CHIRALPAK OJ-3, 25 °C, 0.46 cm ϕ, 25 cm column, 2% *i*PrOH in CO_2, flow rate: 1.2 mL/min; *t*R: 1.97 min (major enantiomer), 2.30 min (minor enantiomer); *ee* = 84%.

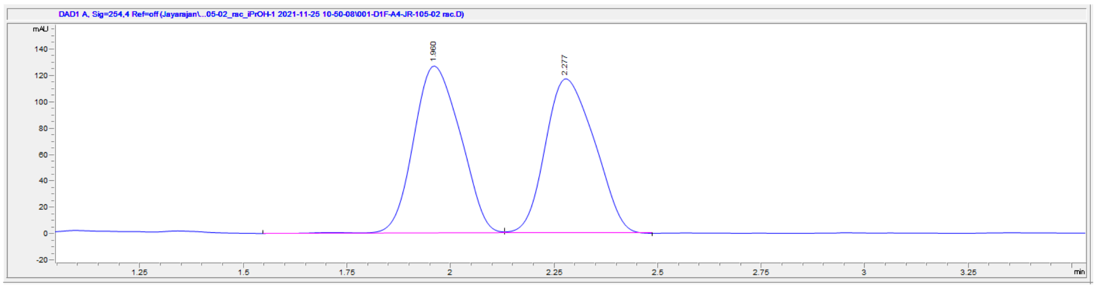

Signal 1: DAD1 A, Sig=254,4 Ref=off

Peak #	RetTime [min]	Type	Width [min]	Area [mAU*s]	Height [mAU]	Area %
1	1.960	VV R	0.1318	999.59631	126.96747	50.8039
2	2.277	VB	0.1383	967.96161	117.20966	49.1961
Totals :				1967.55792	244.17713	

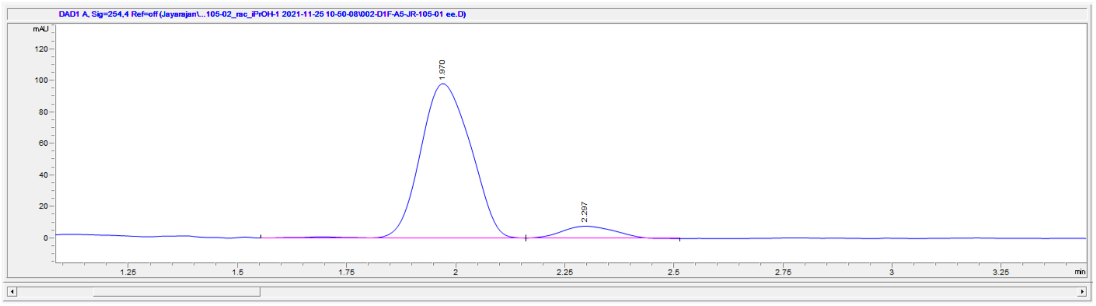

Signal 1: DAD1 A, Sig=254,4 Ref=off

Peak #	RetTime [min]	Type	Width [min]	Area [mAU*s]	Height [mAU]	Area %
1	1.970	VV R	0.1334	783.88300	97.91758	92.1679
2	2.297	VB	0.1423	66.61166	7.74763	7.8321
Totals :				850.49466	105.66521	

3. NMR Spectra

^{1}H NMR ($CDCl_3$, 400 MHz) of compound **5a**

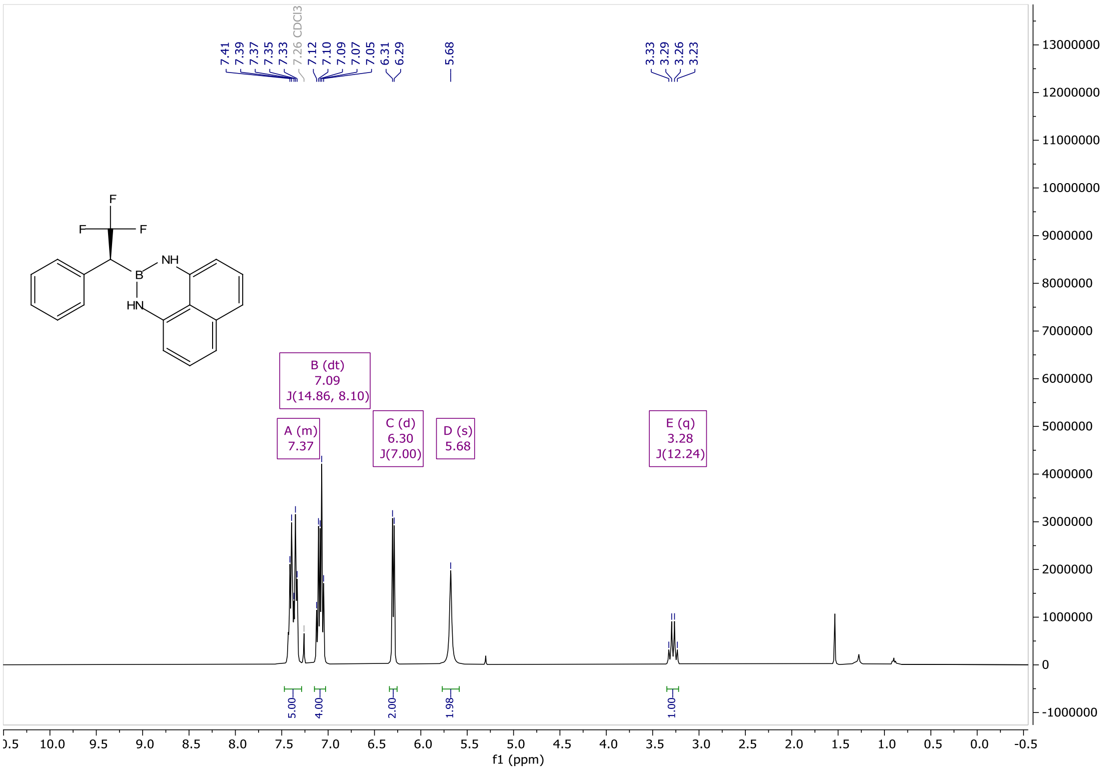

^{13}C NMR ($CDCl_3$, 101 MHz) of compound **5a**

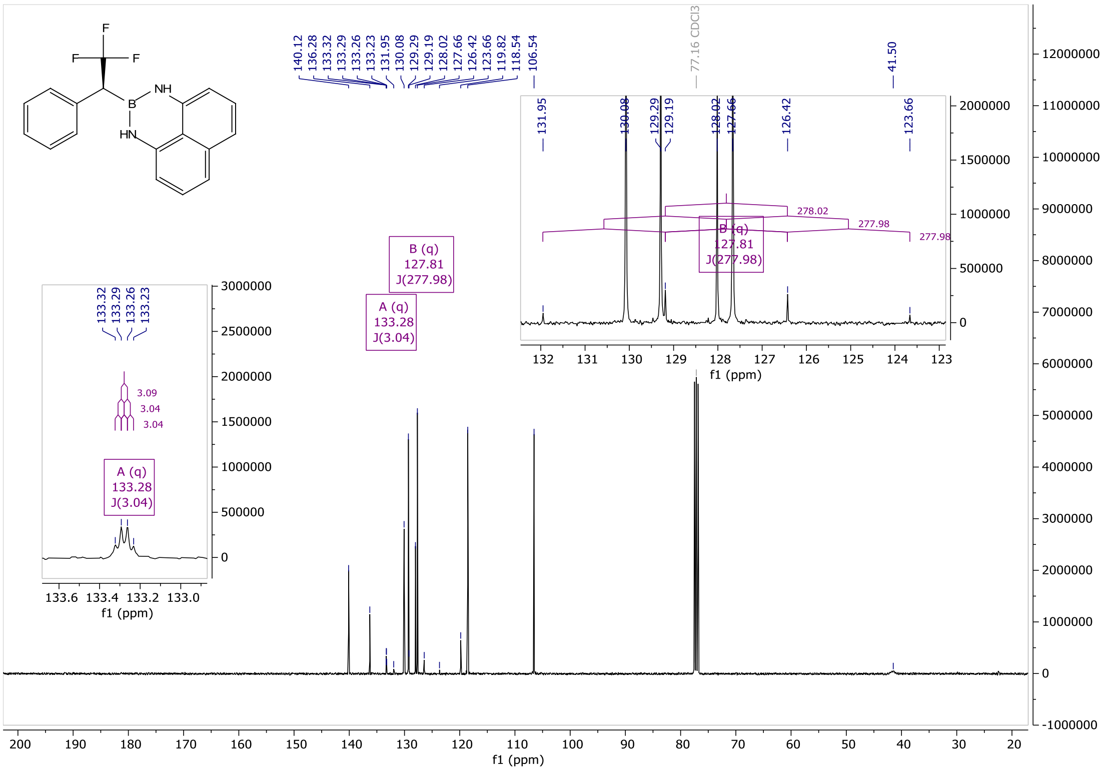

^{19}F NMR ($CDCl_3$, 377 MHz) of compound **5a**

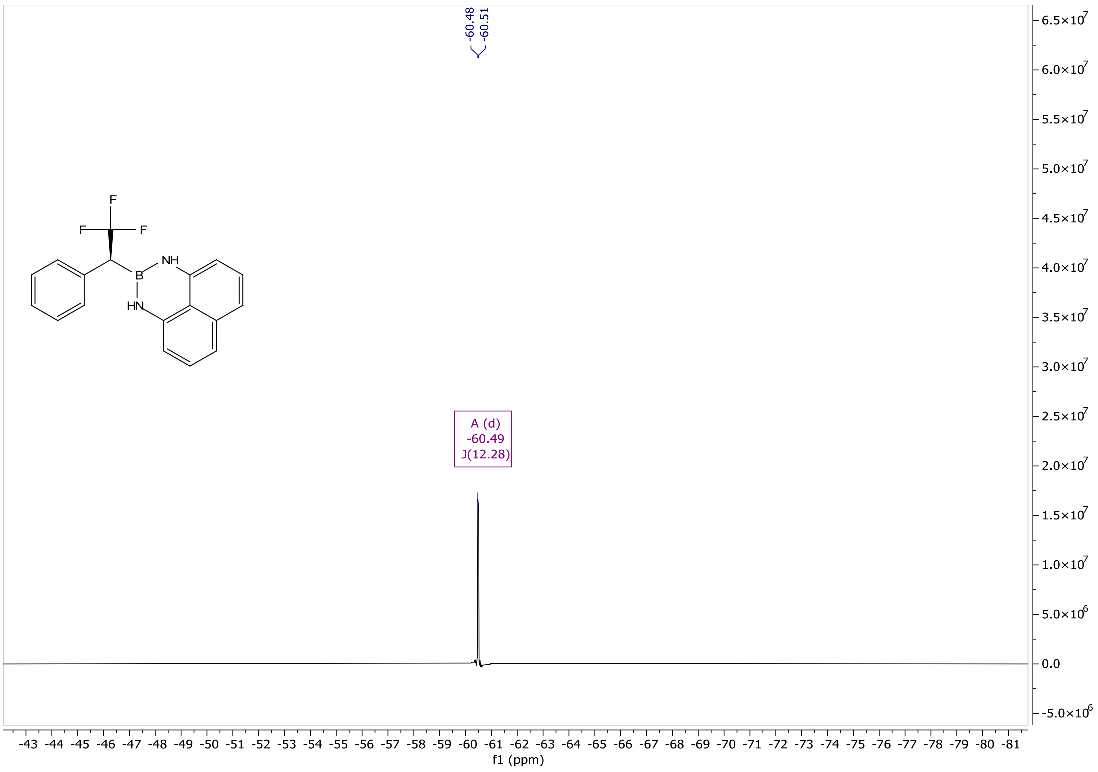

^{11}B NMR ($CDCl_3$, 128 MHz) of compound **5a**

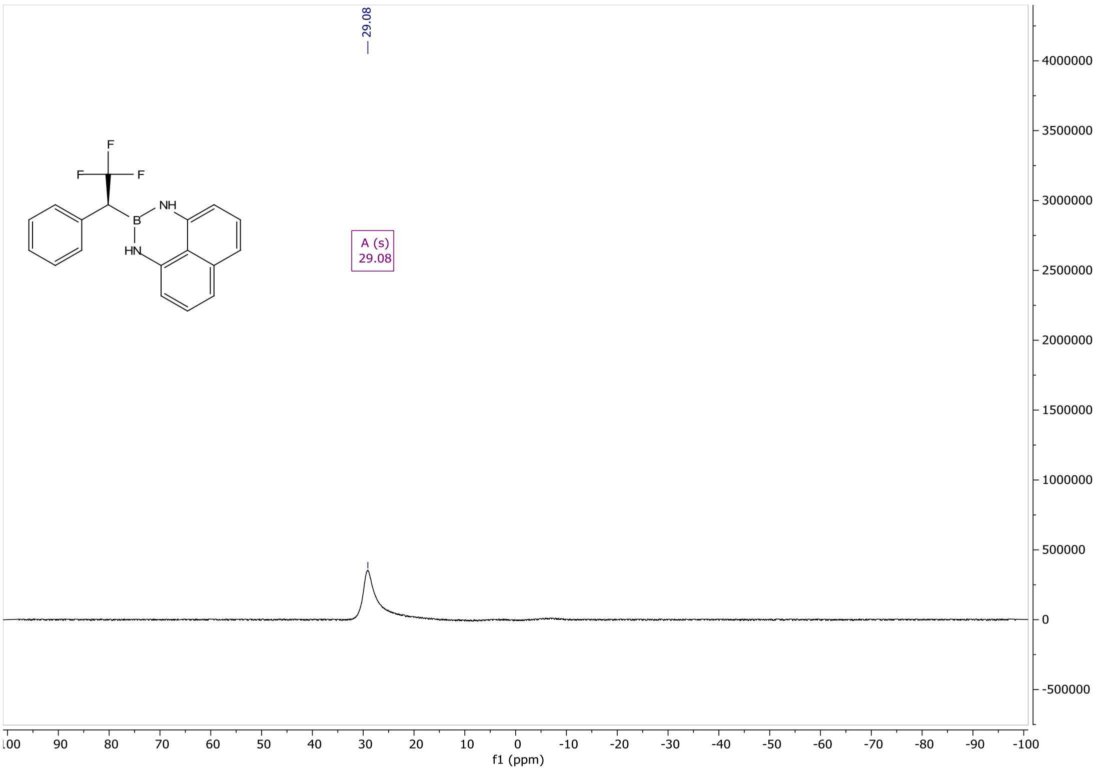

^{1}H NMR ($CDCl_3$, 400 MHz) of compound **5b**

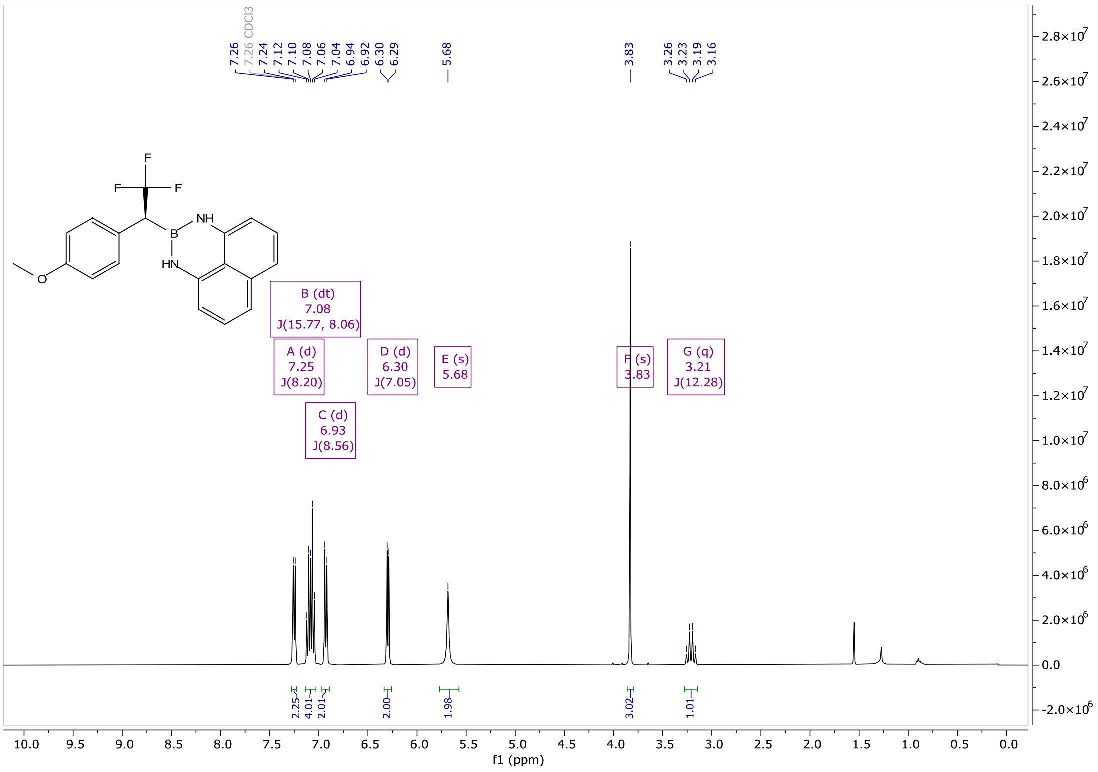

^{13}C NMR ($CDCl_3$, 101 MHz) of compound **5b**

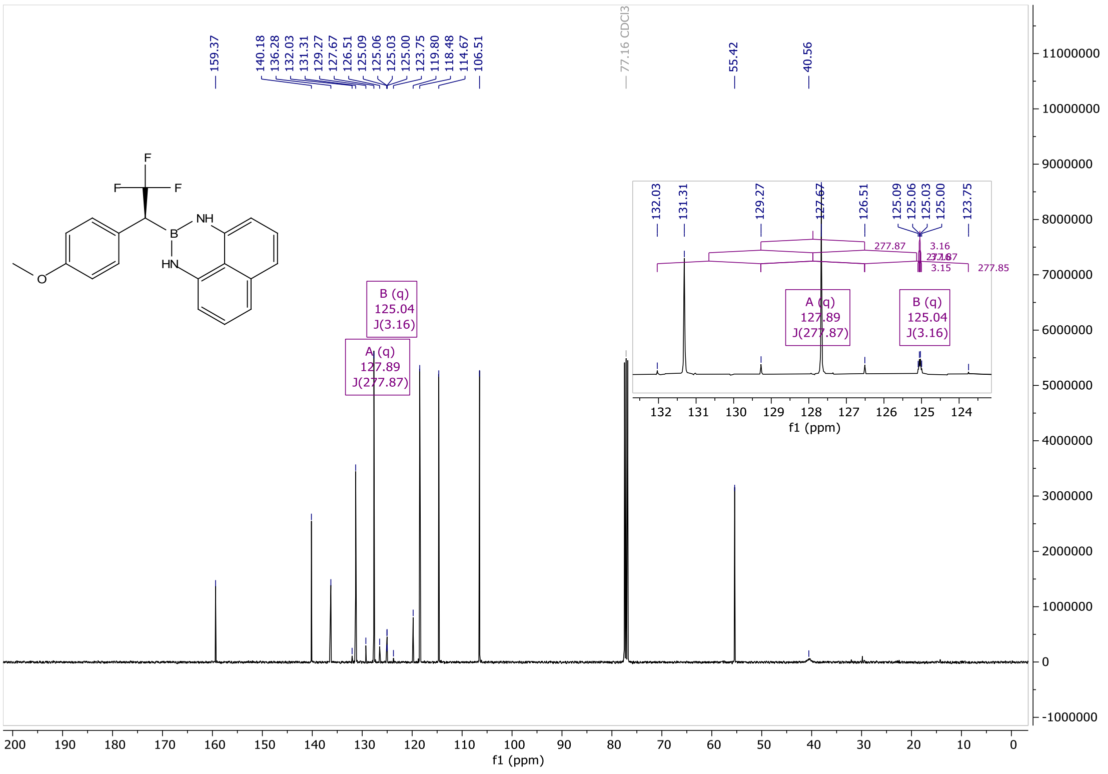

^{19}F NMR ($CDCl_3$, 377 MHz) of compound **5b**

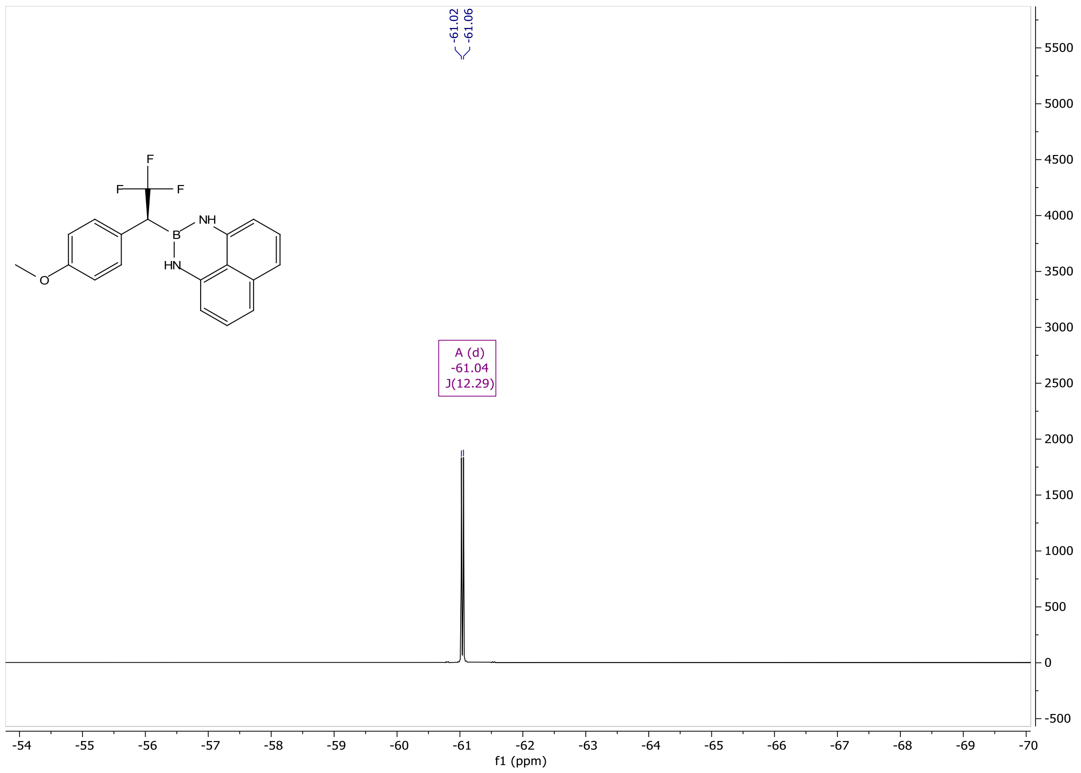

^{11}B NMR ($CDCl_3$, 128 MHz) of compound **5b**

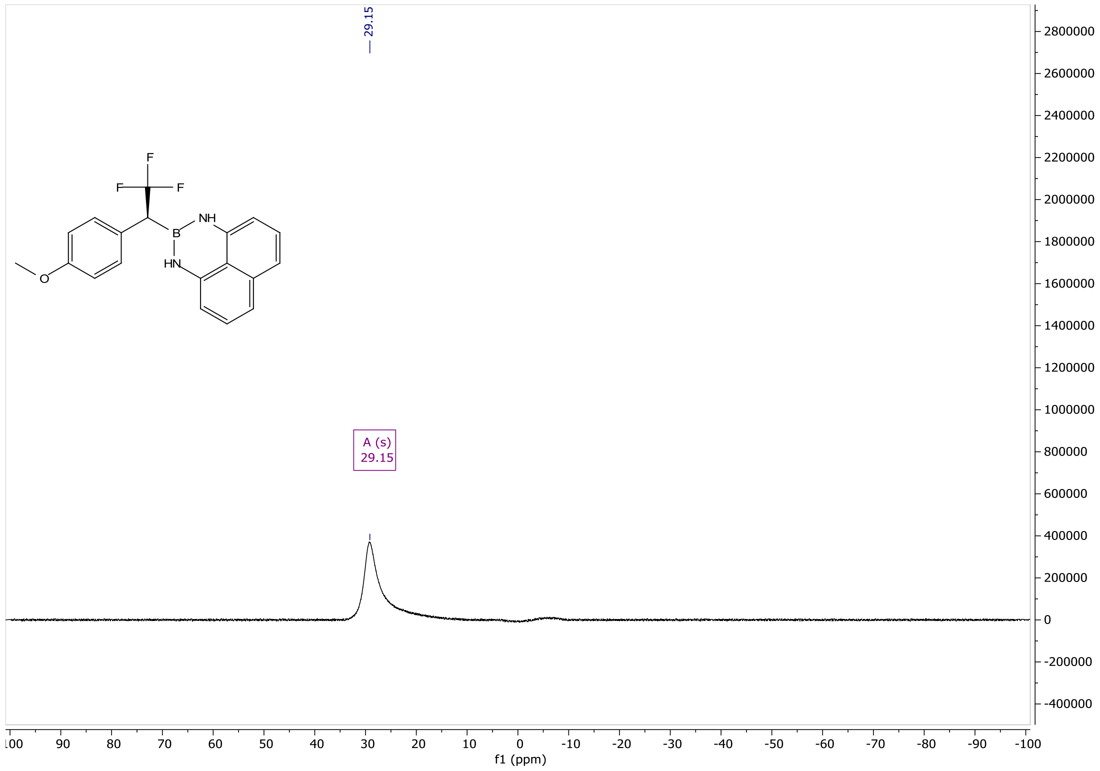

^{1}H NMR ($CDCl_3$, 400 MHz) of compound **5c**

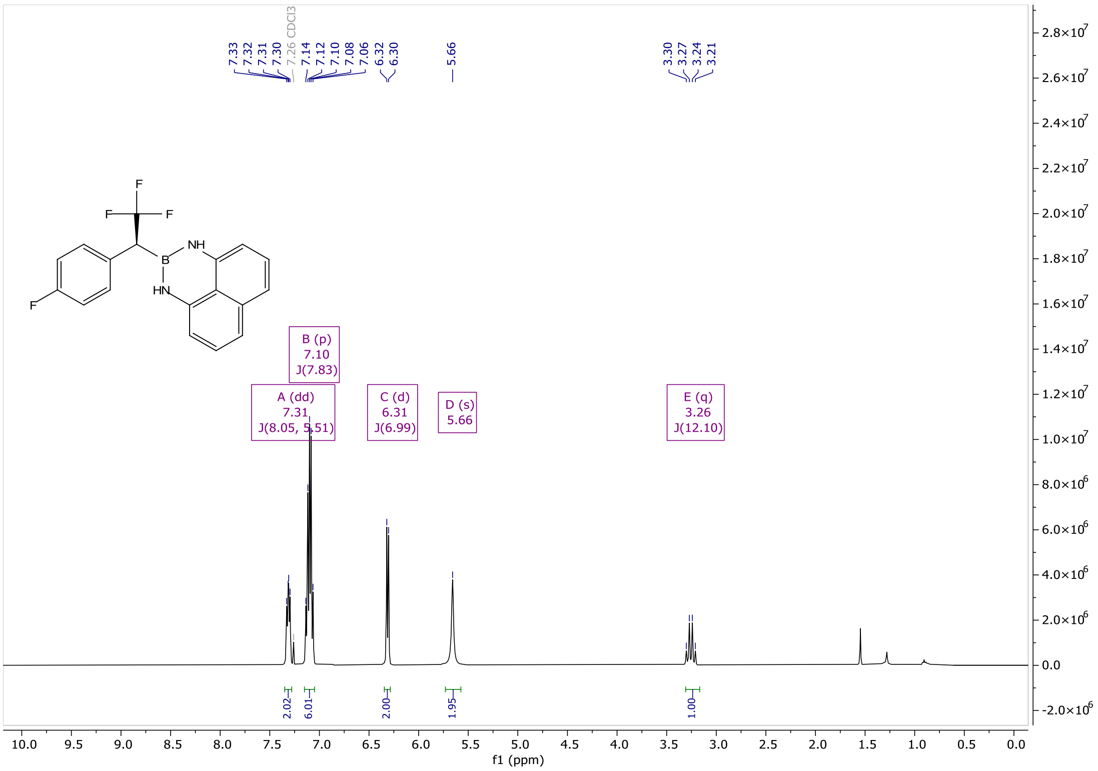

^{13}C NMR ($CDCl_3$, 101 MHz) of compound **5c**

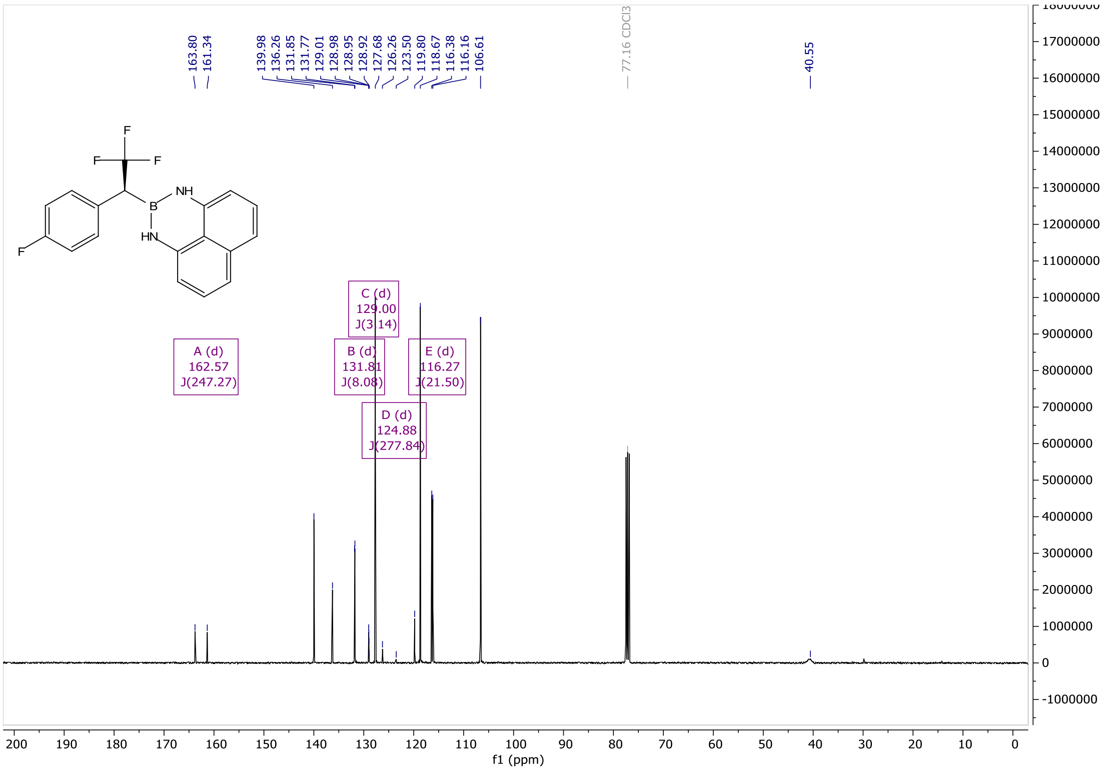

^{19}F NMR ($CDCl_3$, 377 MHz) of compound **5c**

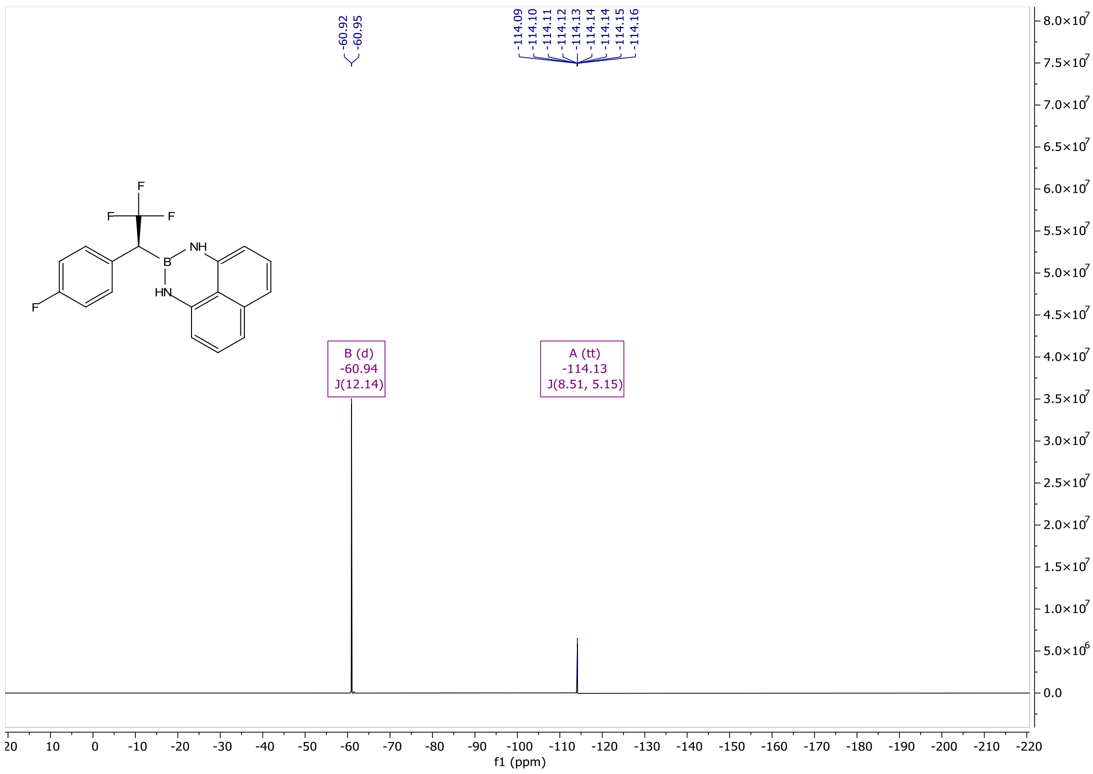

^{11}B NMR ($CDCl_3$, 128 MHz) of compound **5c**

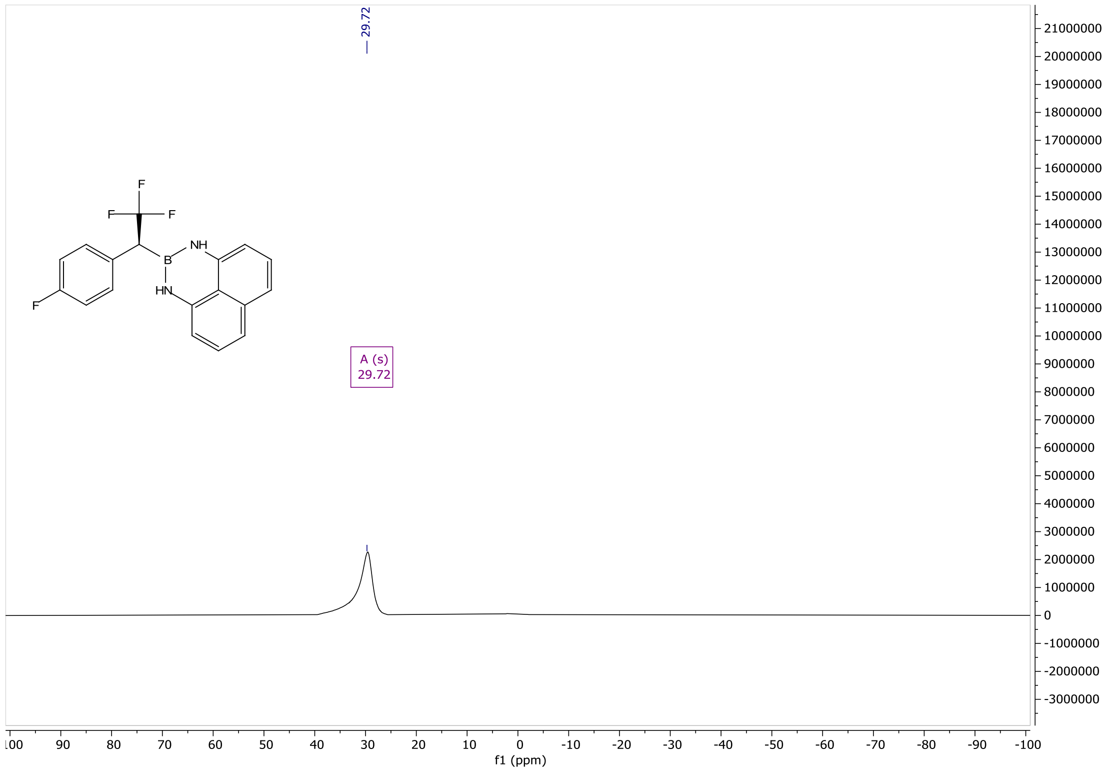

^{1}H NMR ($CDCl_3$, 400 MHz) of compound **5d**

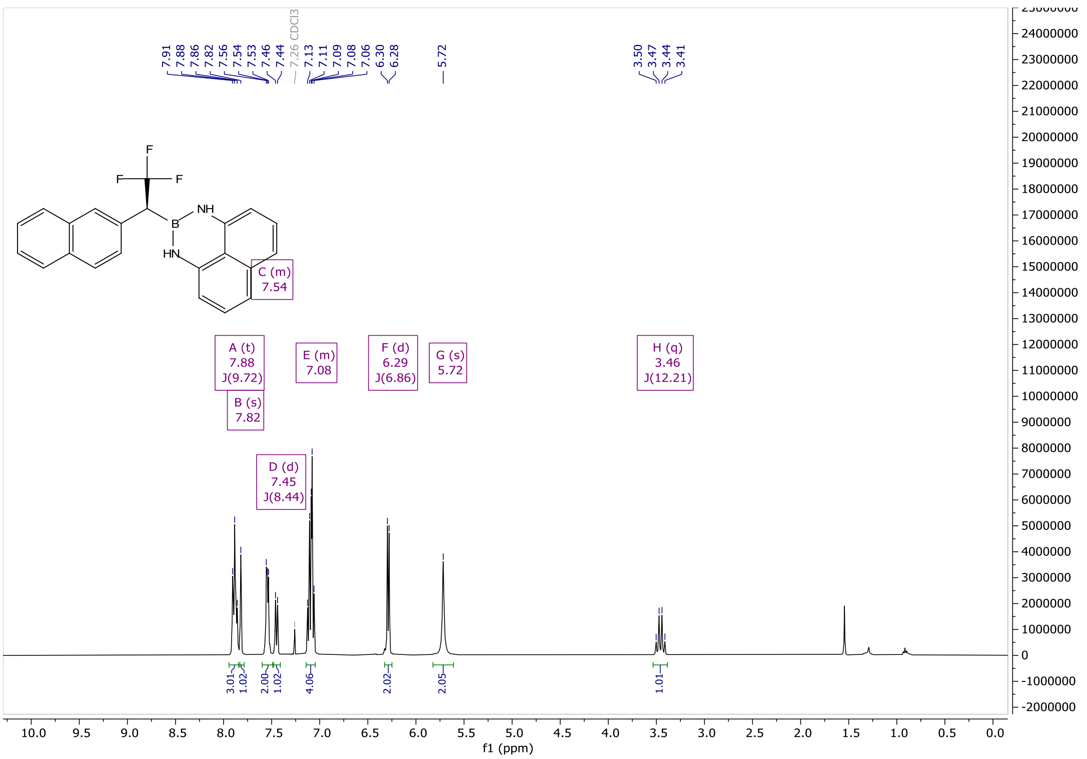

^{13}C NMR ($CDCl_3$, 101 MHz) of compound **5d**

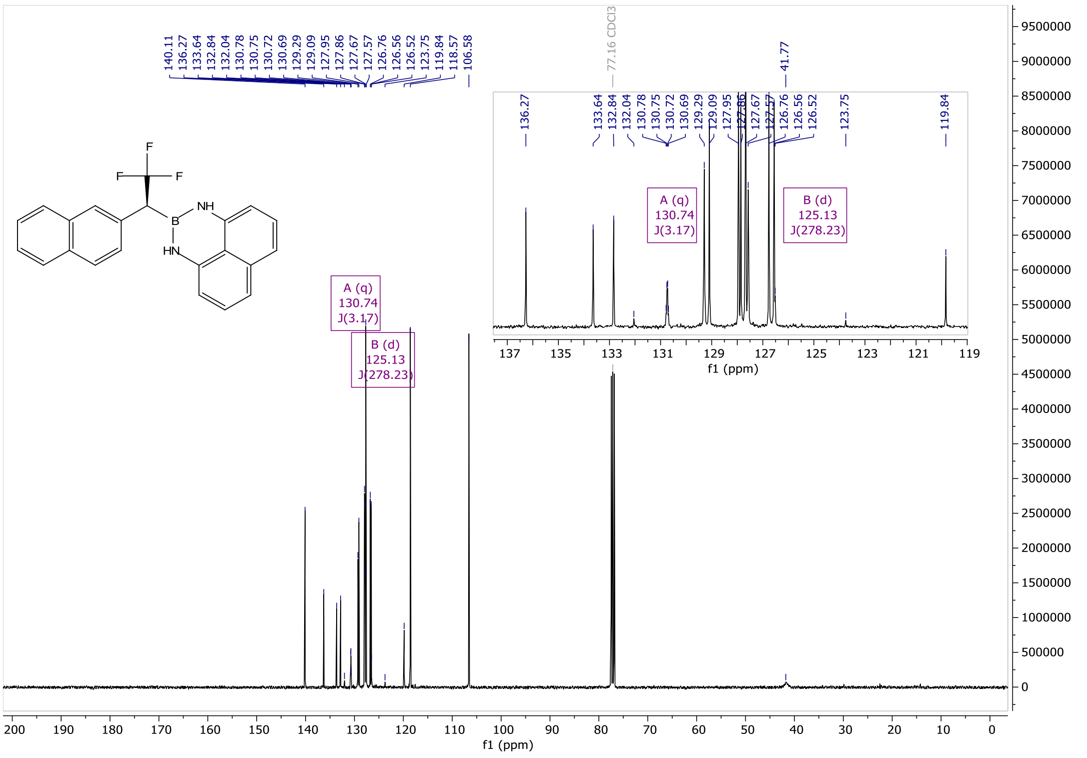

^{19}F NMR ($CDCl_3$, 377 MHz) of compound **5d**

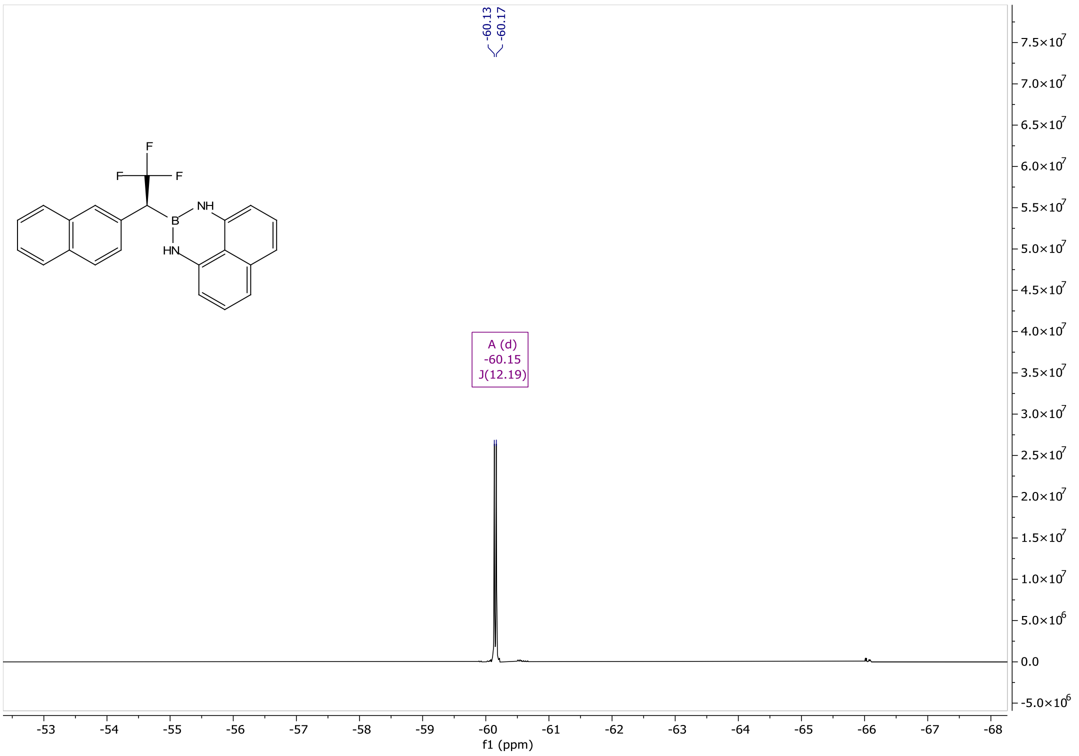

^{11}B NMR ($CDCl_3$, 128 MHz) of compound **5d**

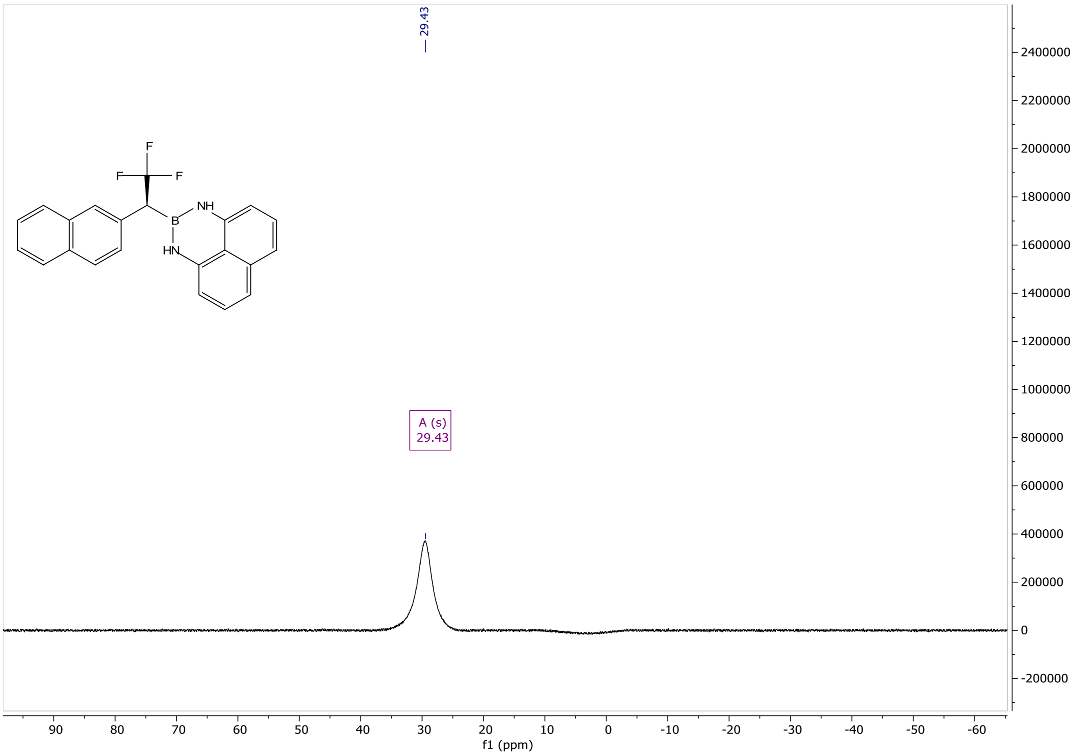

^{1}H NMR ($CDCl_3$, 400 MHz) of compound **5e**

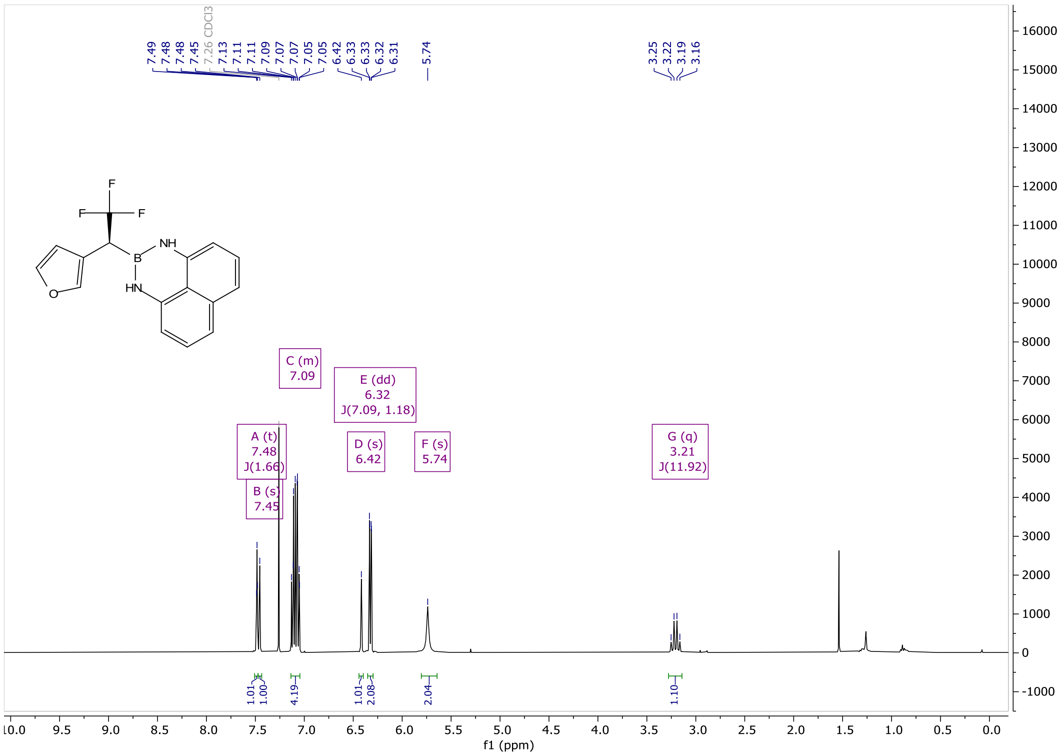

^{13}C NMR ($CDCl_3$, 101 MHz) of compound **5e**

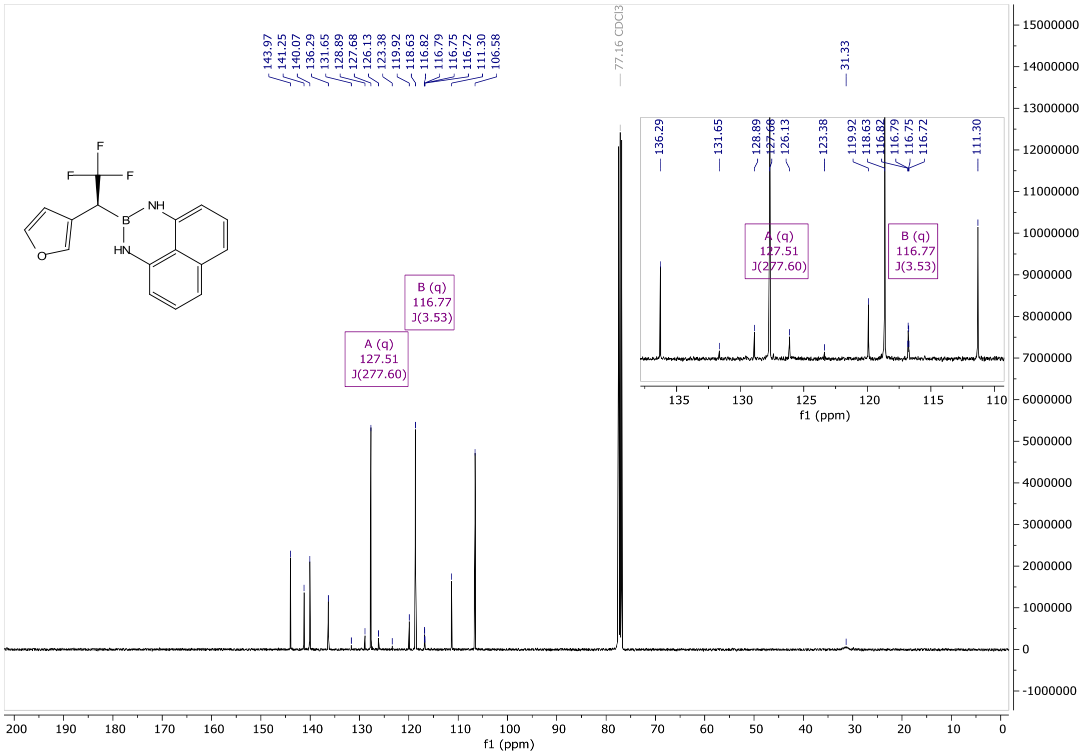

^{19}F NMR ($CDCl_3$, 377 MHz) of compound **5e**

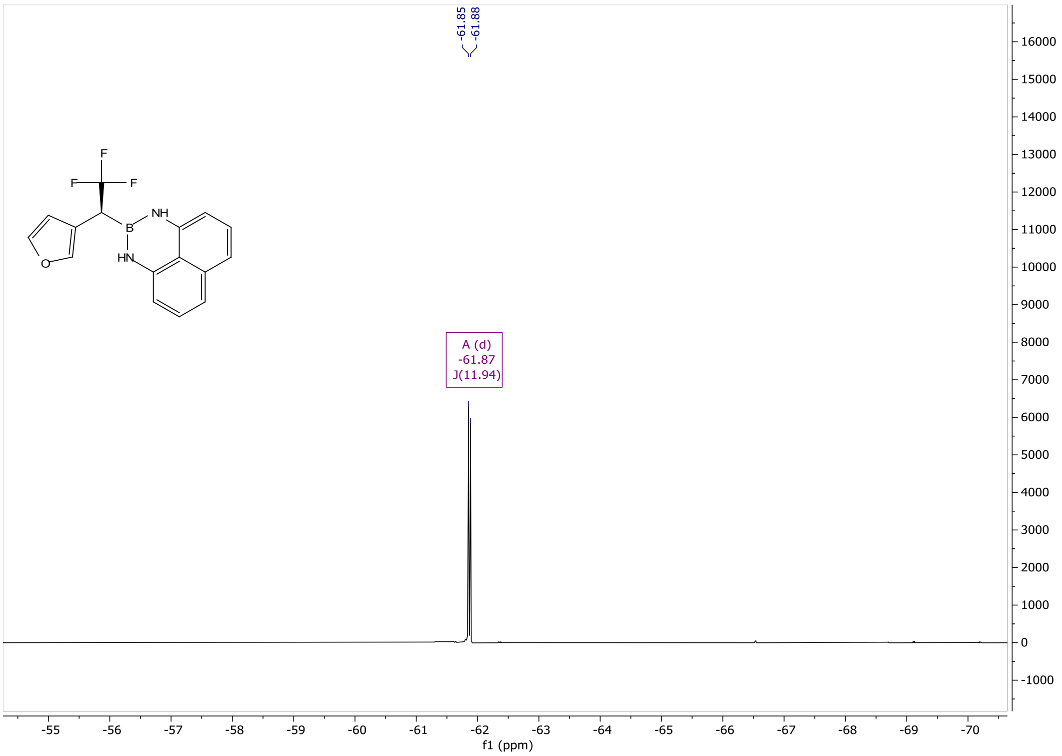

^{11}B NMR ($CDCl_3$, 128 MHz) of compound **5e**

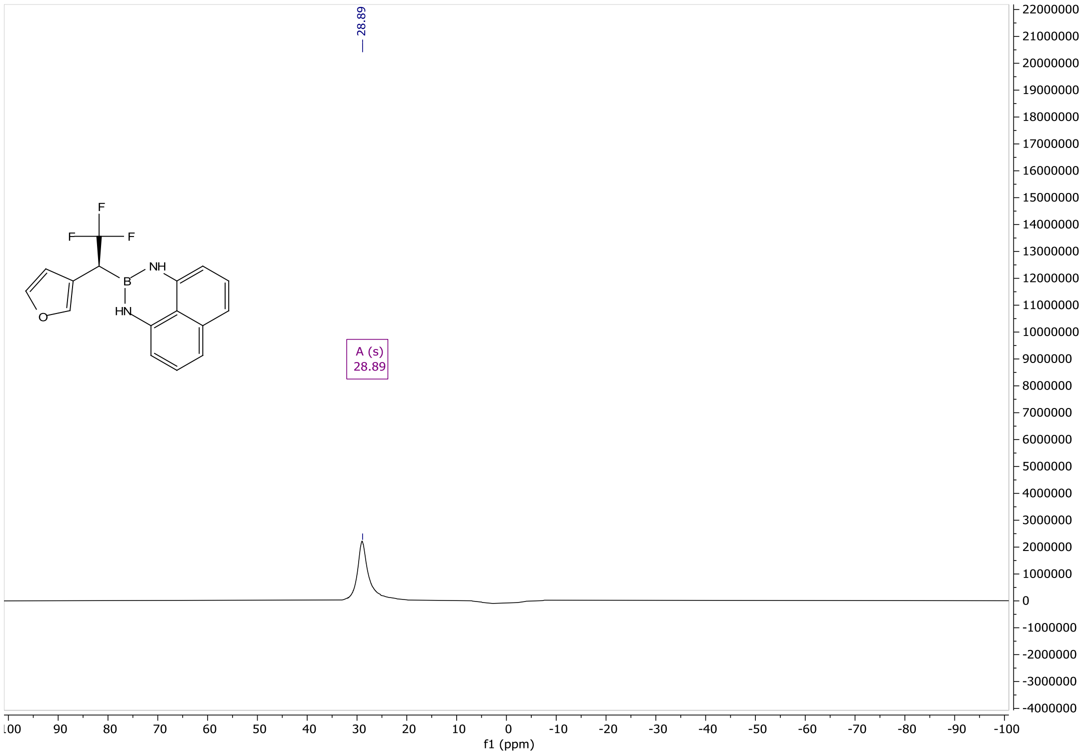

^{1}H NMR ($CDCl_3$, 400 MHz) of compound **5f**

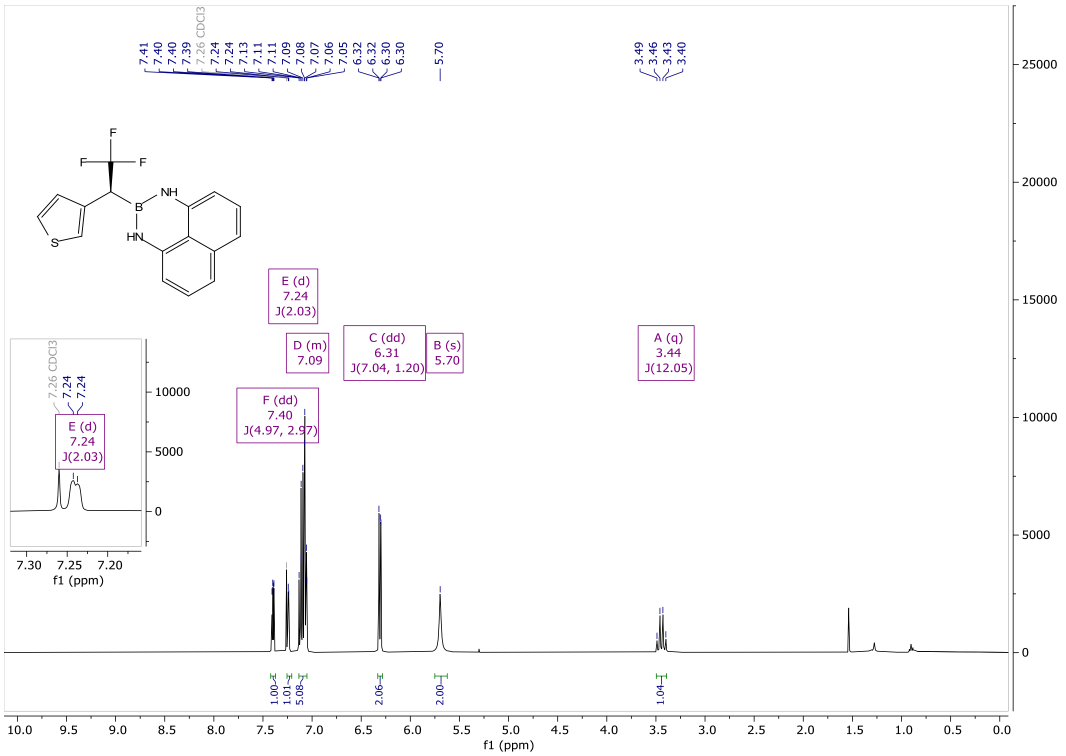

^{13}C NMR ($CDCl_3$, 101 MHz) of compound **5f**

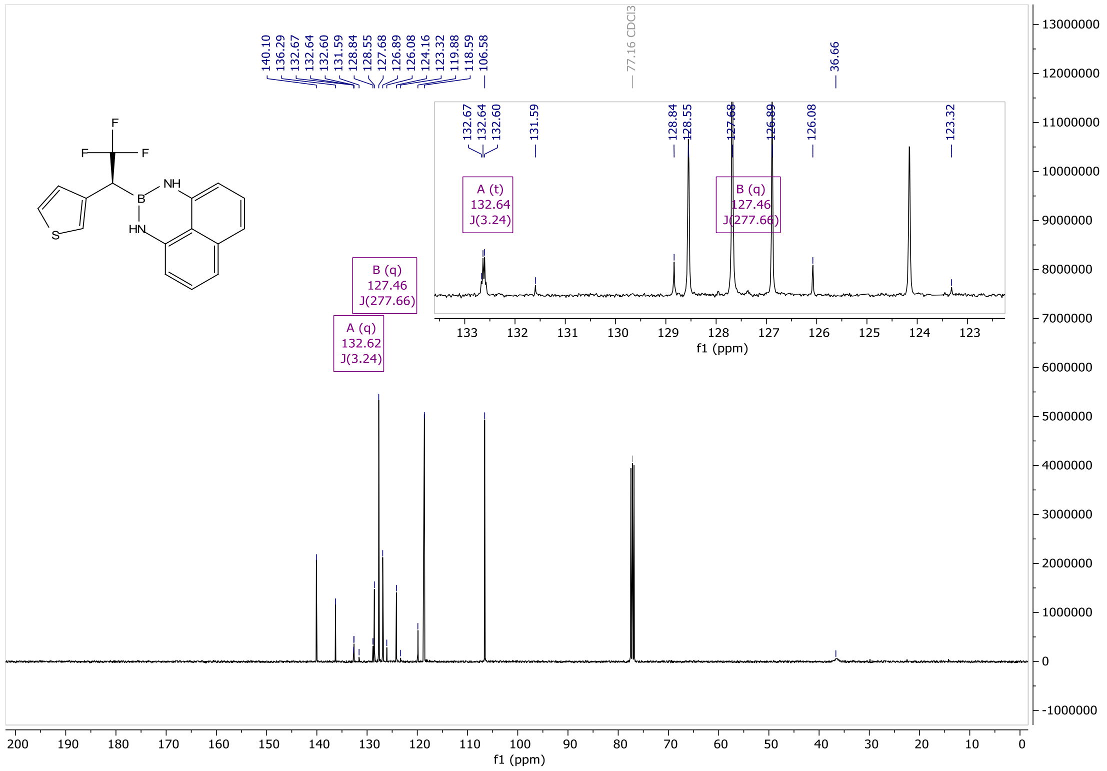

^{19}F NMR ($CDCl_3$, 377 MHz) of compound **5f**

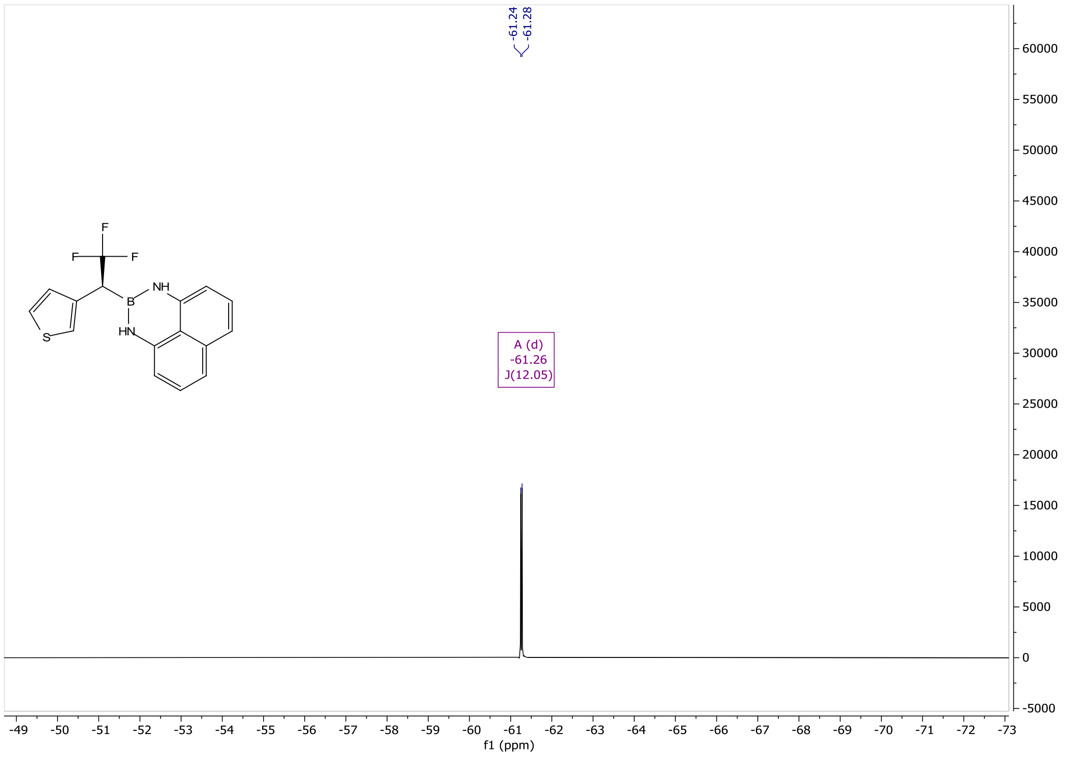

^{11}B NMR ($CDCl_3$, 128 MHz) of compound **5f**

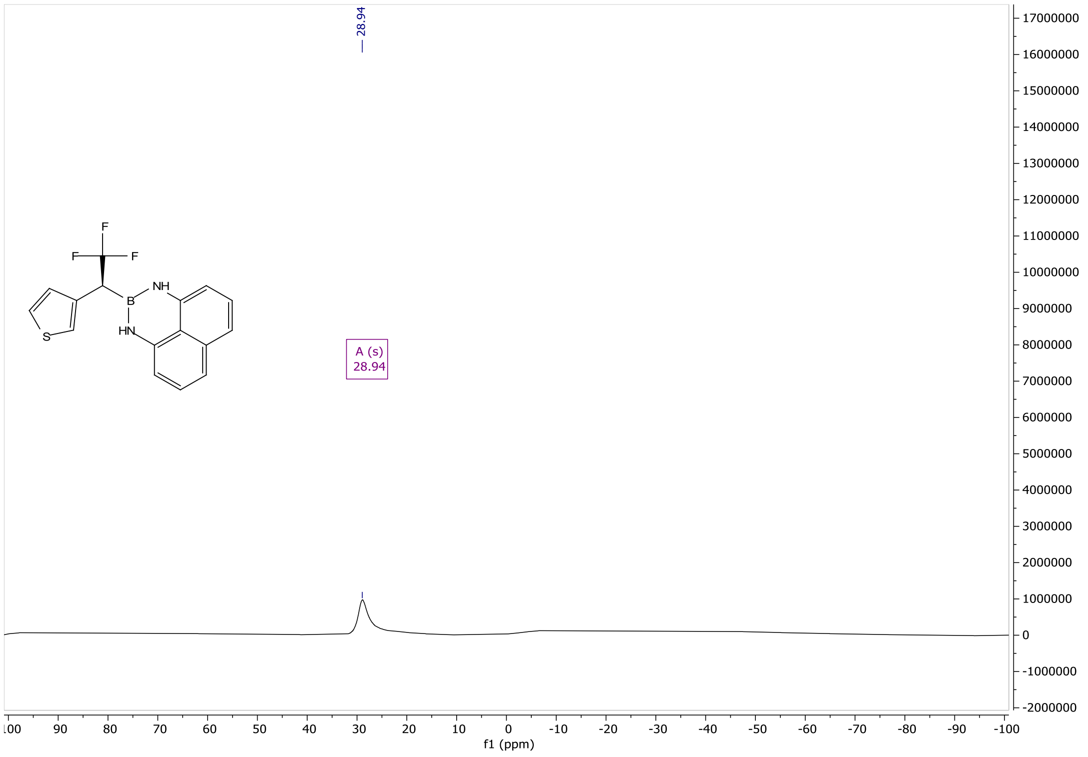

^{1}H NMR ($CDCl_3$, 400 MHz) of compound **5g**

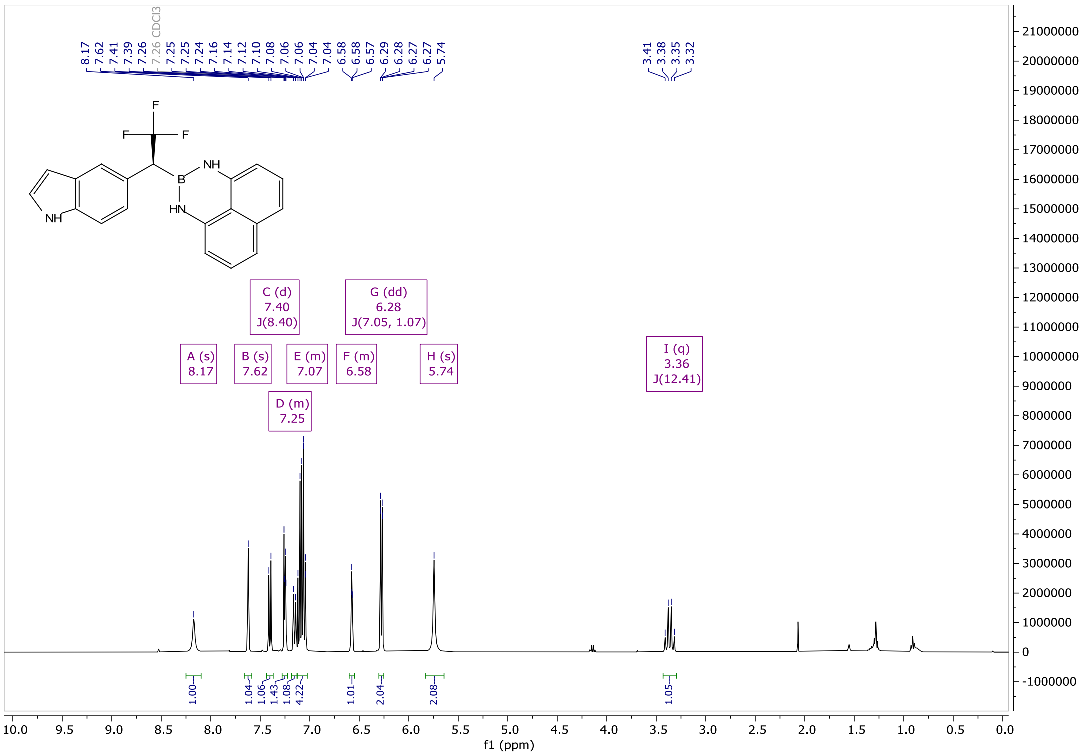

^{13}C NMR ($CDCl_3$, 101 MHz) of compound **5g**

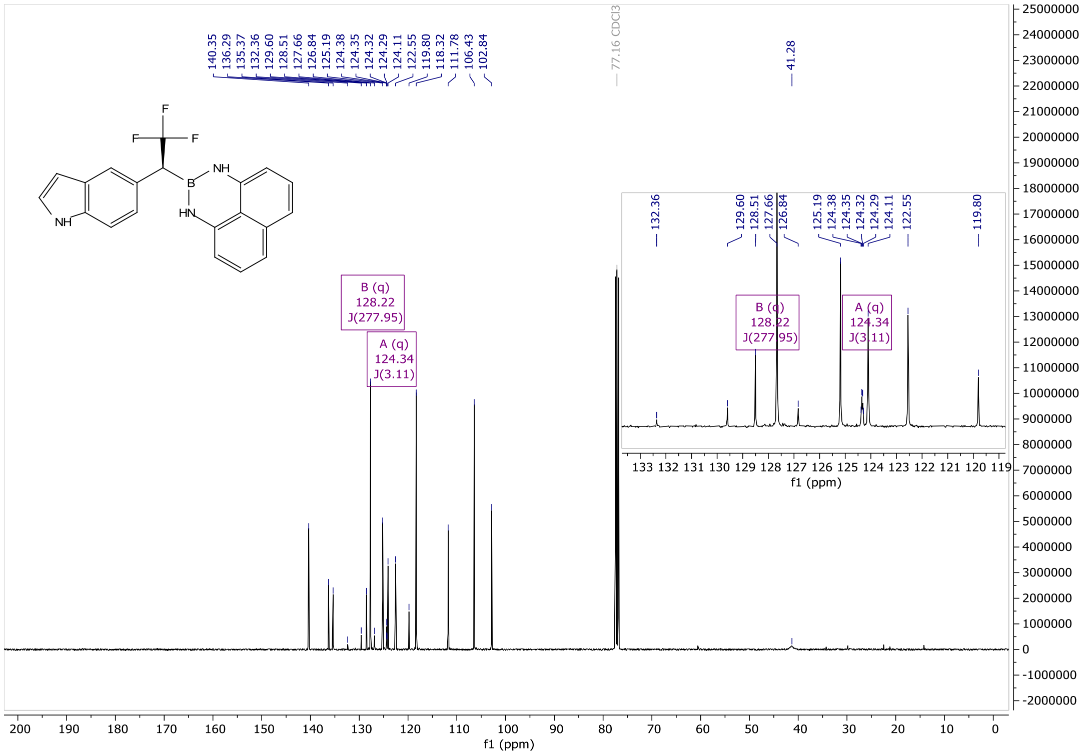

^{19}F NMR ($CDCl_3$, 377 MHz) of compound **5g**

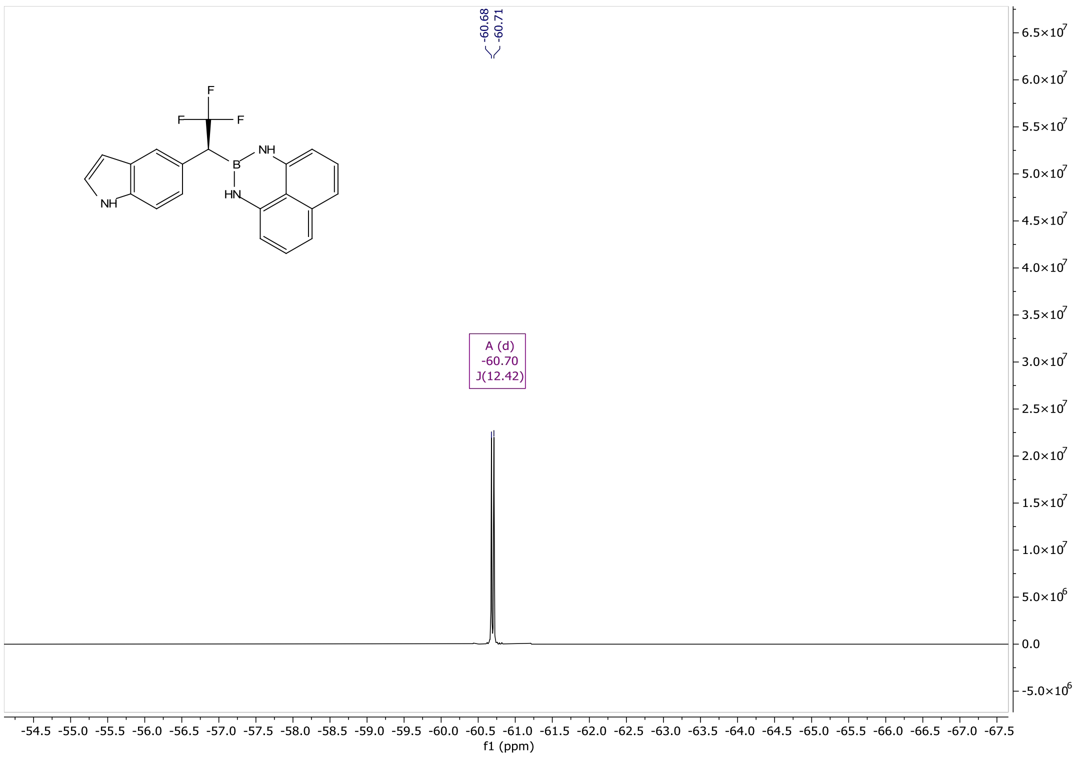

^{11}B NMR ($CDCl_3$, 128 MHz) of compound **5g**

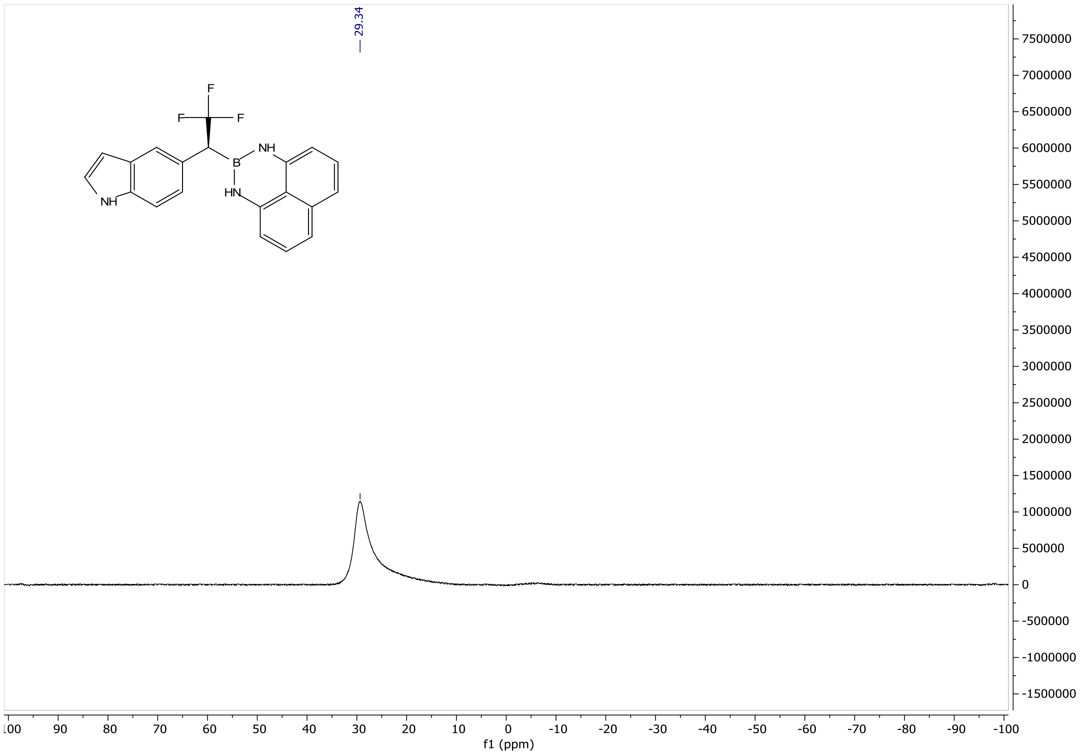

^{1}H NMR ($CDCl_3$, 400 MHz) of compound **5h**

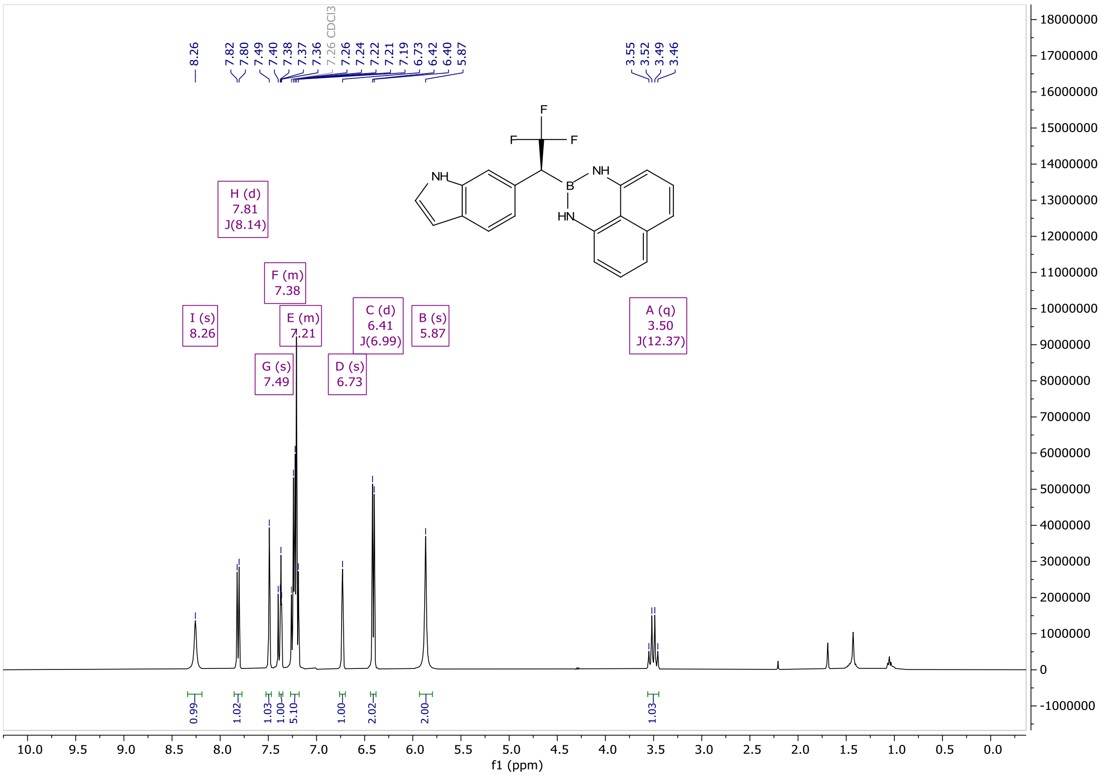

^{13}C NMR ($CDCl_3$, 101 MHz) of compound **5h**

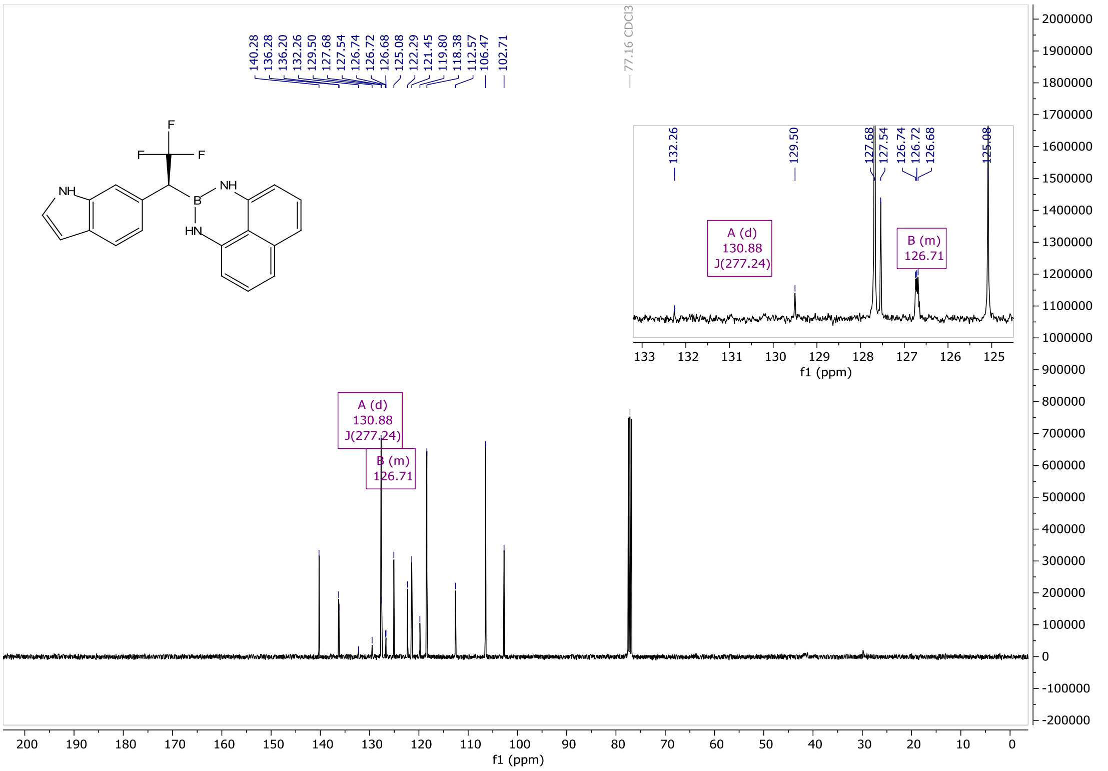

^{19}F NMR ($CDCl_3$, 377 MHz) of compound **5h**

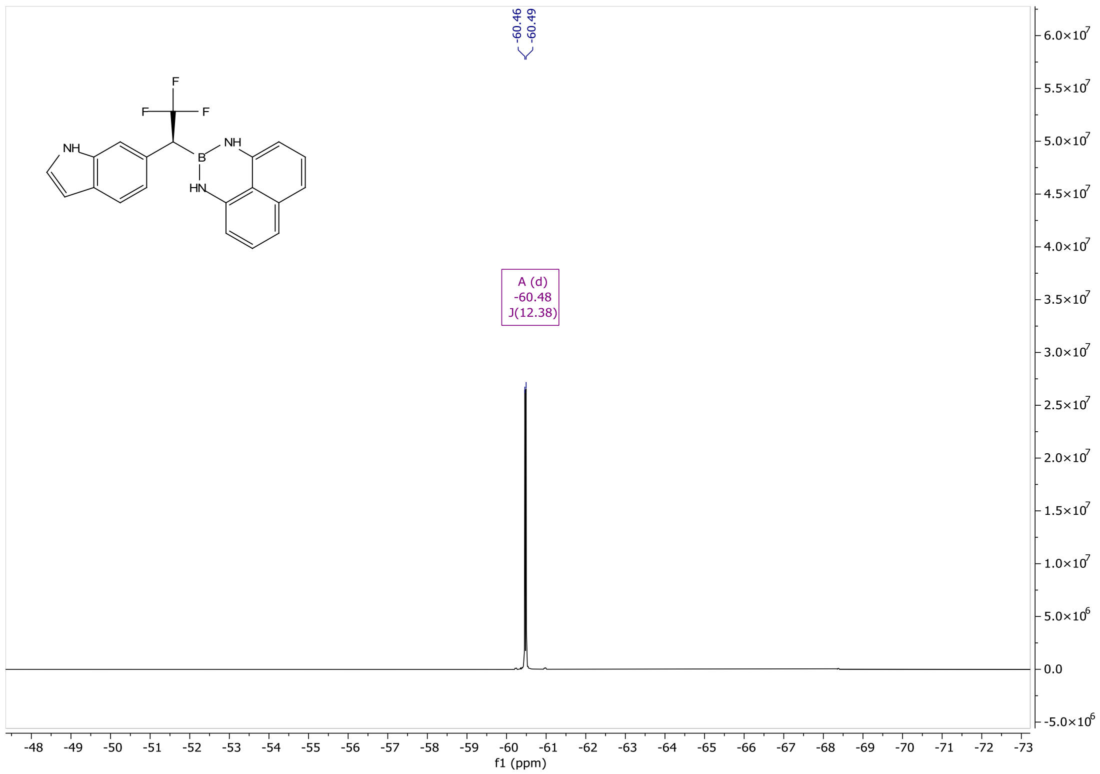

^{11}B NMR ($CDCl_3$, 128 MHz) of compound **5h**

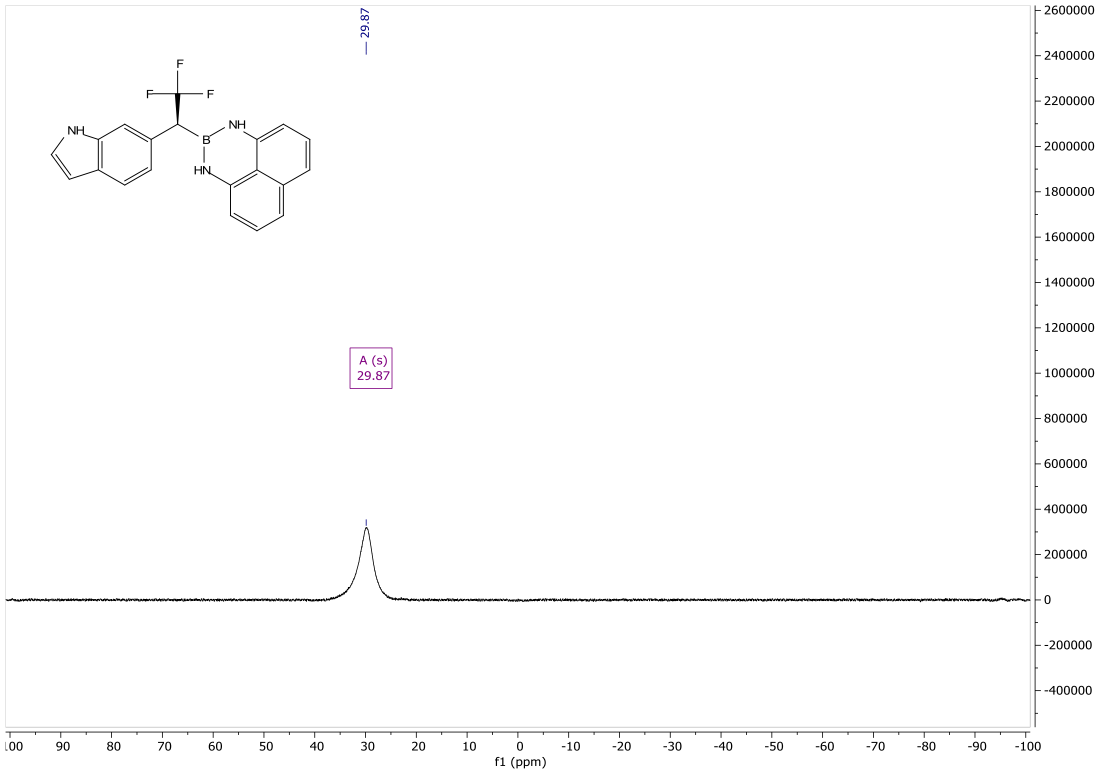

^{1}H NMR ($CDCl_3$, 400 MHz) of compound **5i**

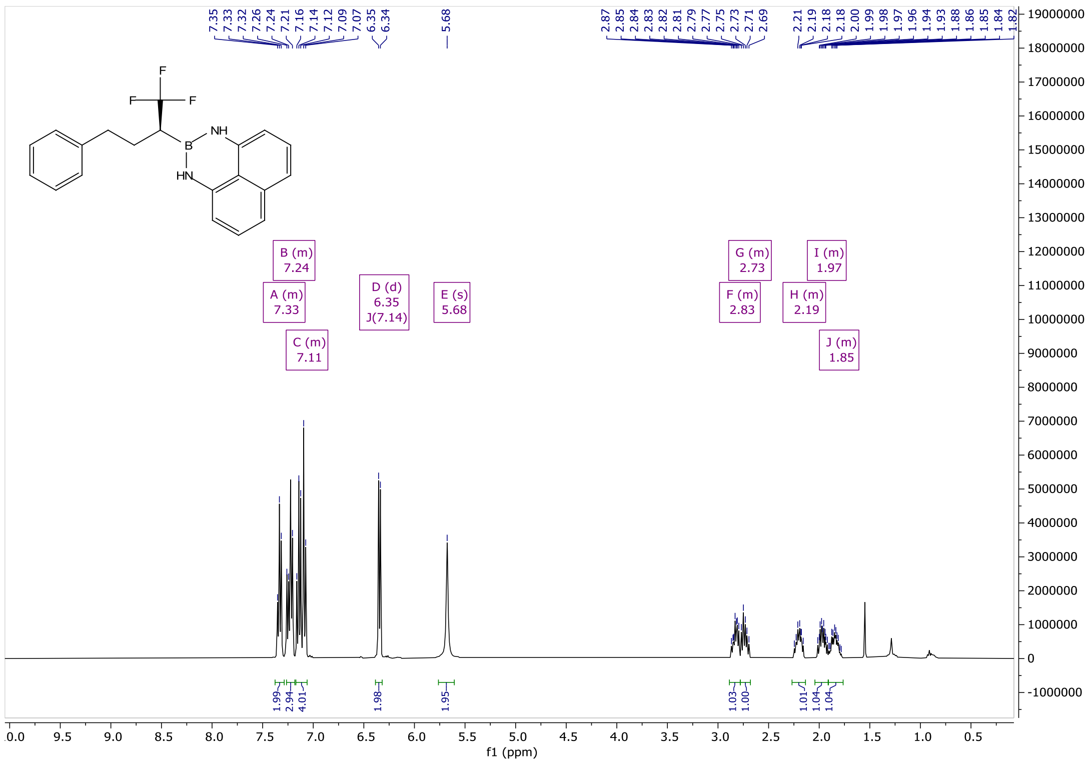

^{13}C NMR ($CDCl_3$, 101 MHz) of compound **5i**

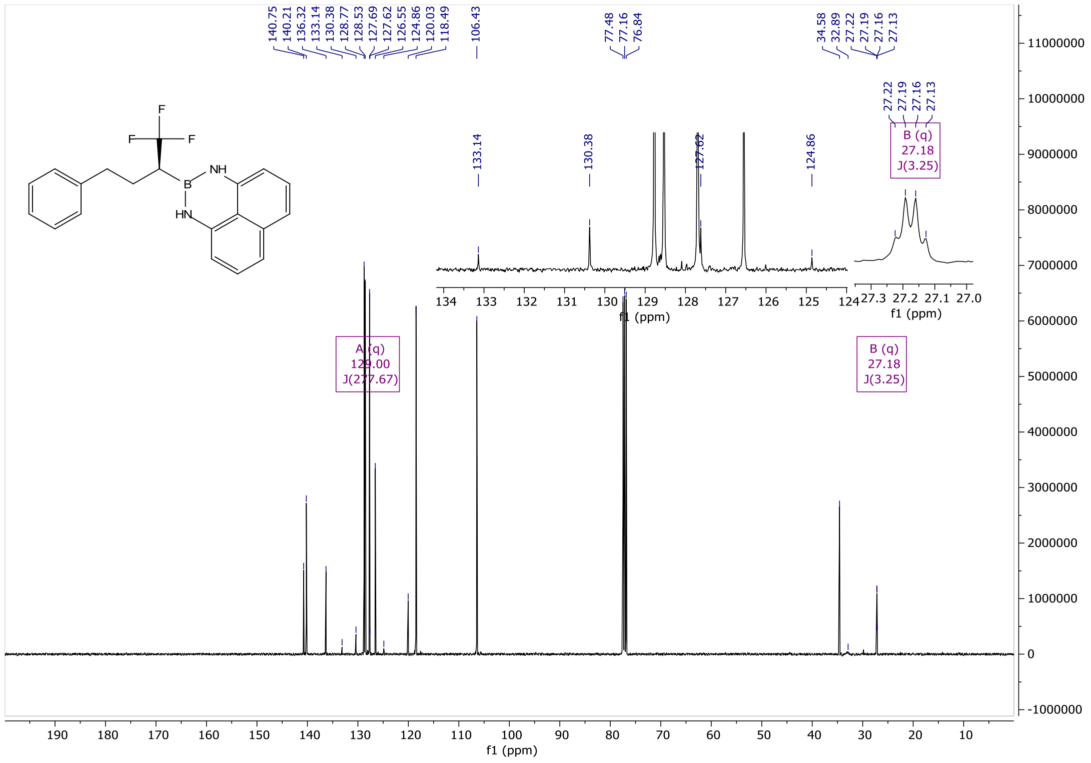

^{19}F NMR ($CDCl_3$, 377 MHz) of compound **5i**

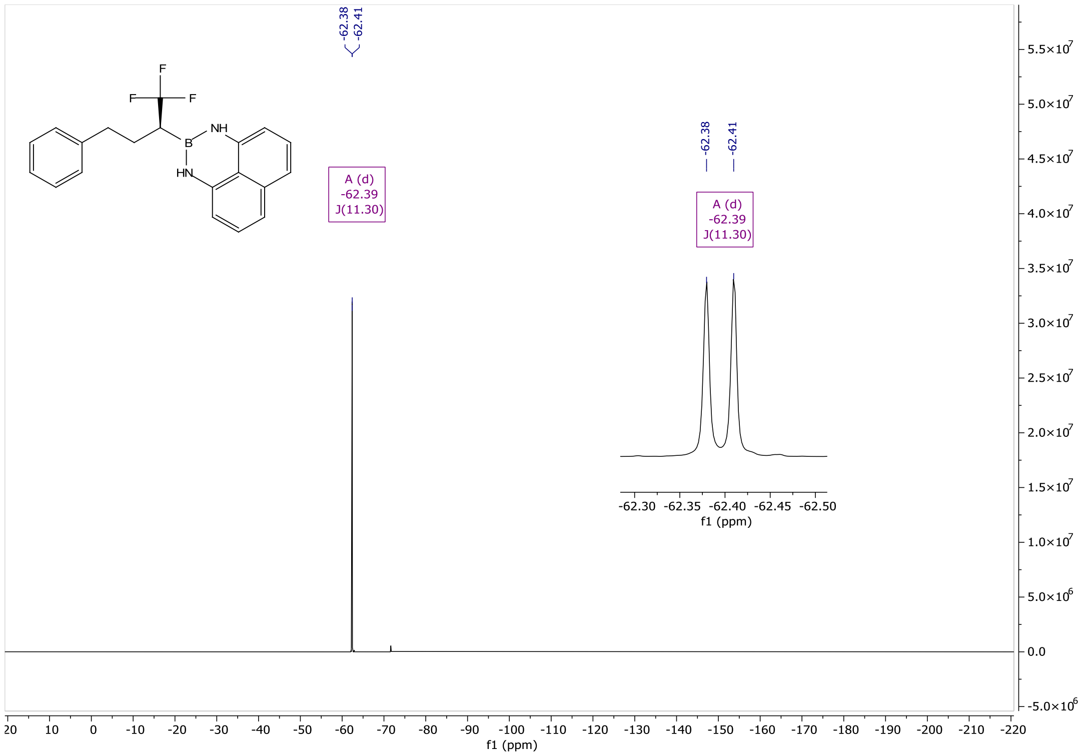

^{11}B NMR ($CDCl_3$, 128 MHz) of compound **5i**

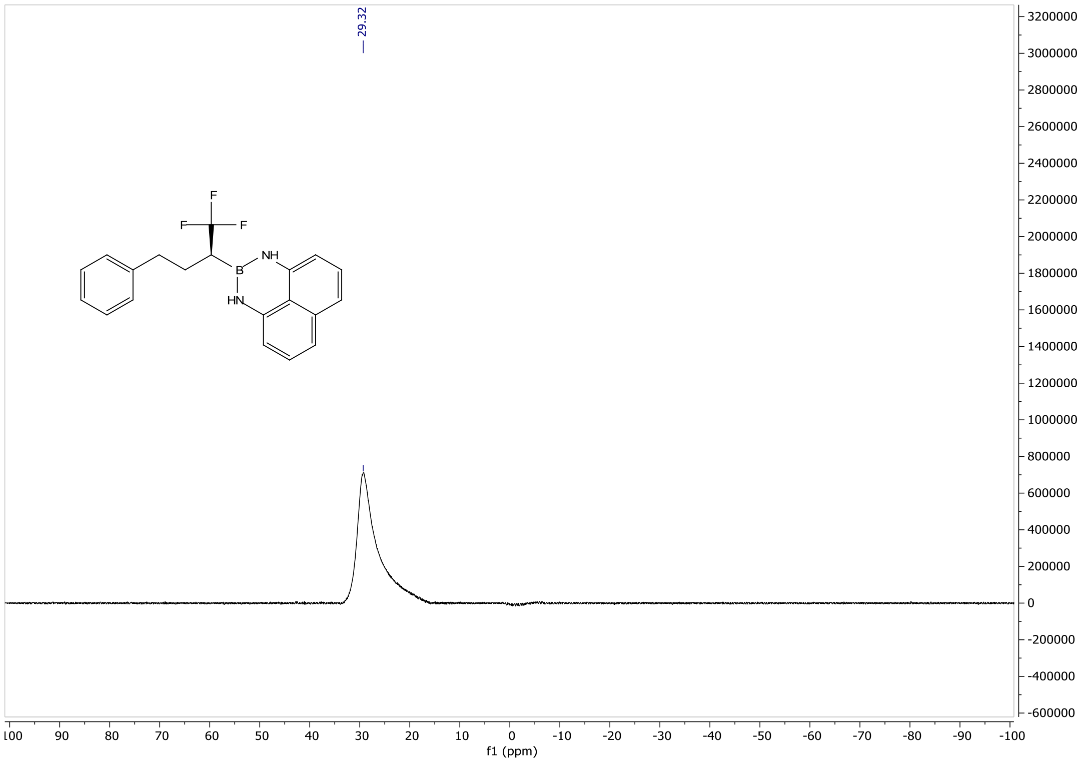

^{1}H NMR ($CDCl_3$, 400 MHz) of compound **5j**

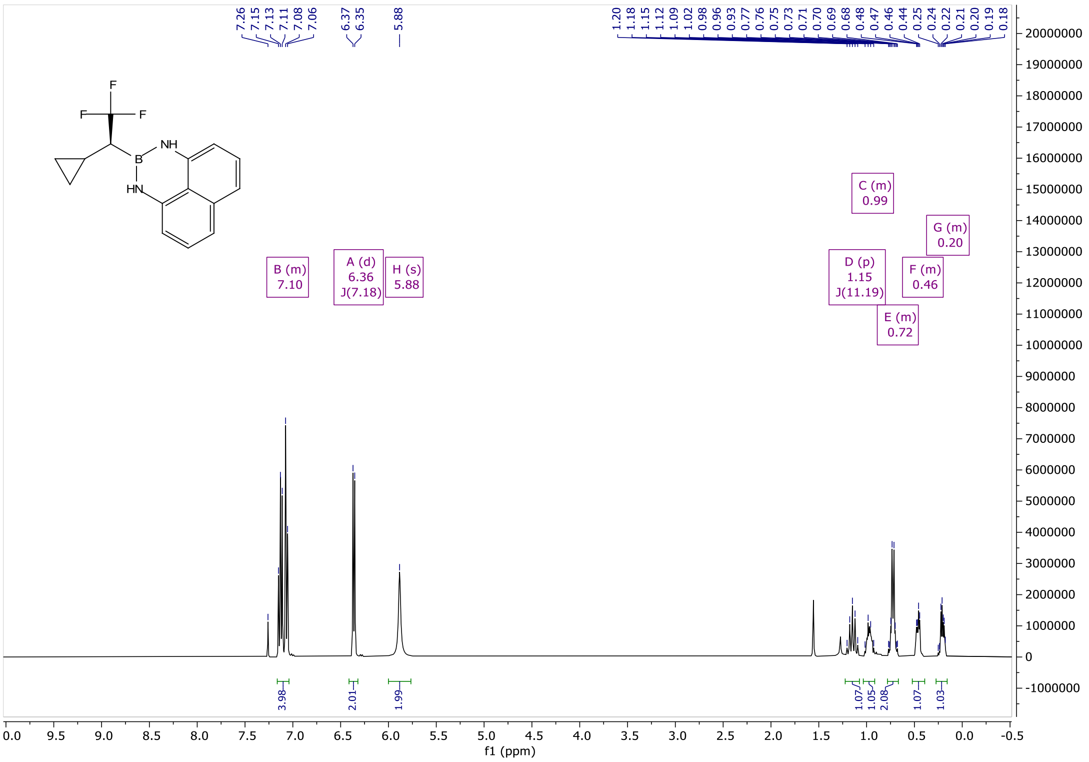

^{13}C NMR ($CDCl_3$, 101 MHz) of compound **5j**

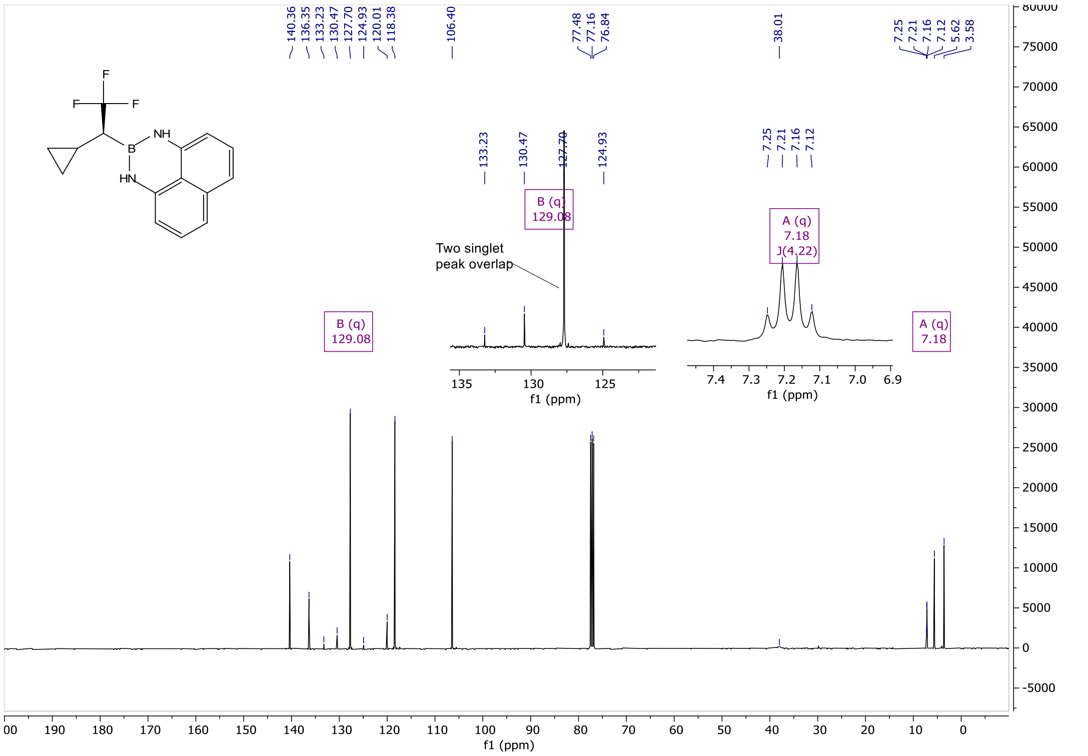

^{19}F NMR ($CDCl_3$, 377 MHz) of compound **5j**

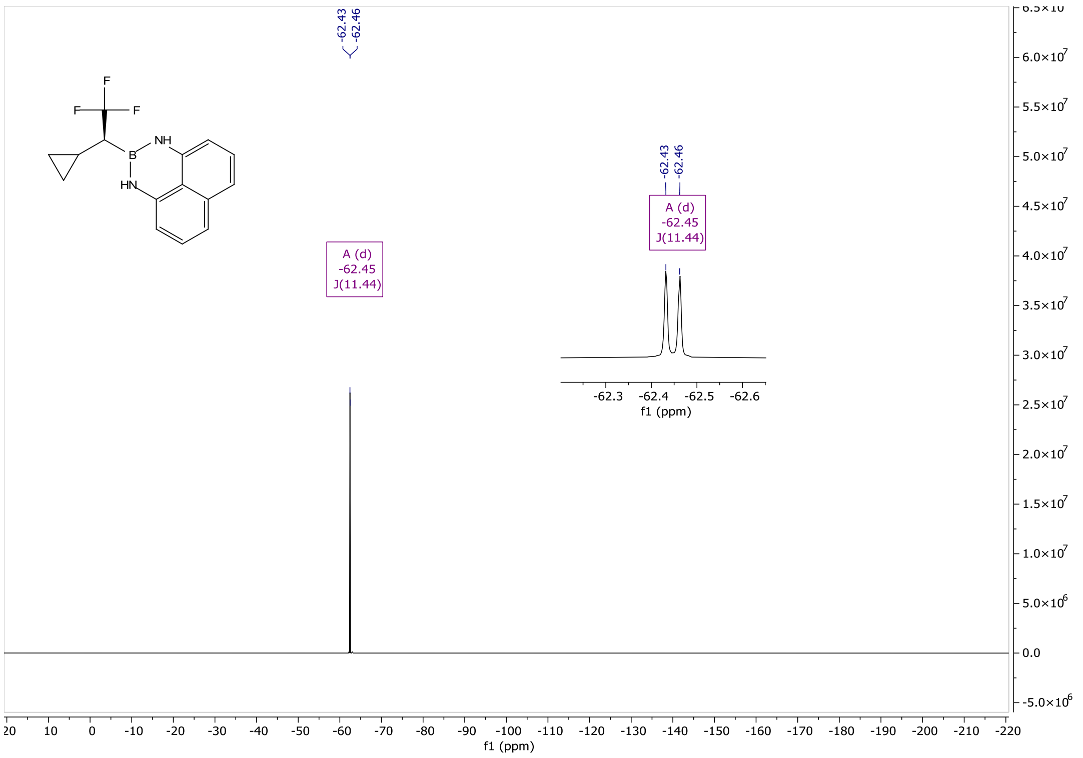

^{11}B NMR ($CDCl_3$, 128 MHz) of compound **5j**

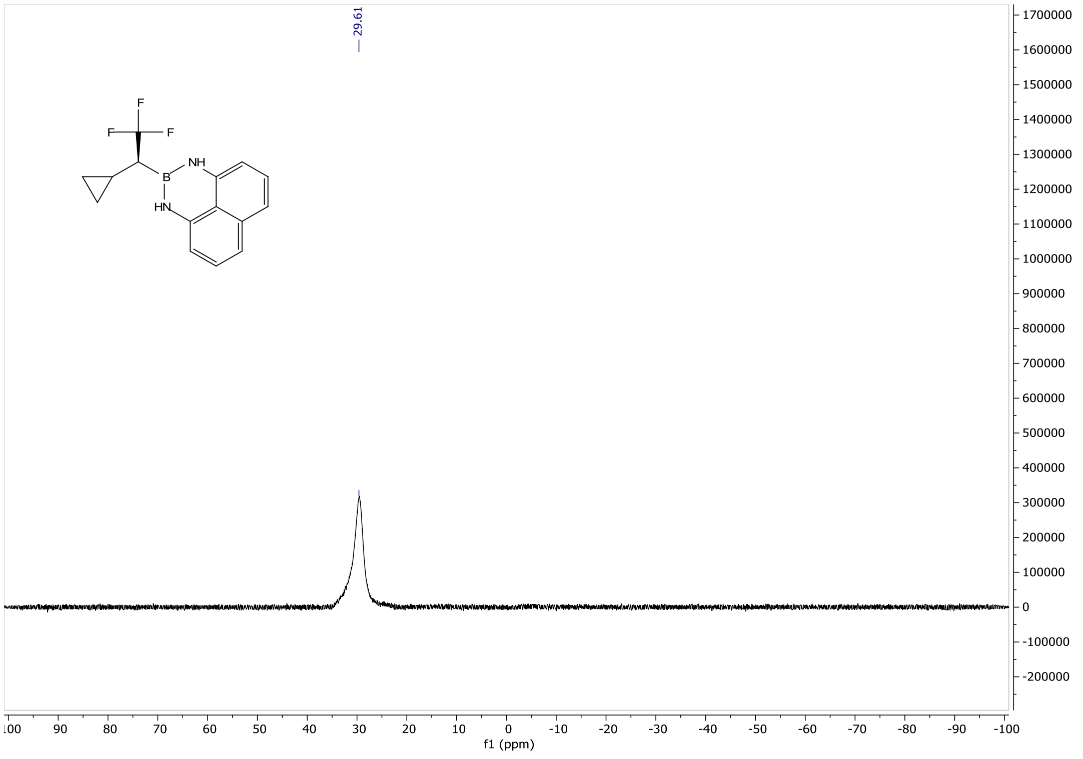

^{1}H NMR ($CDCl_3$, 400 MHz) of compound **5k**

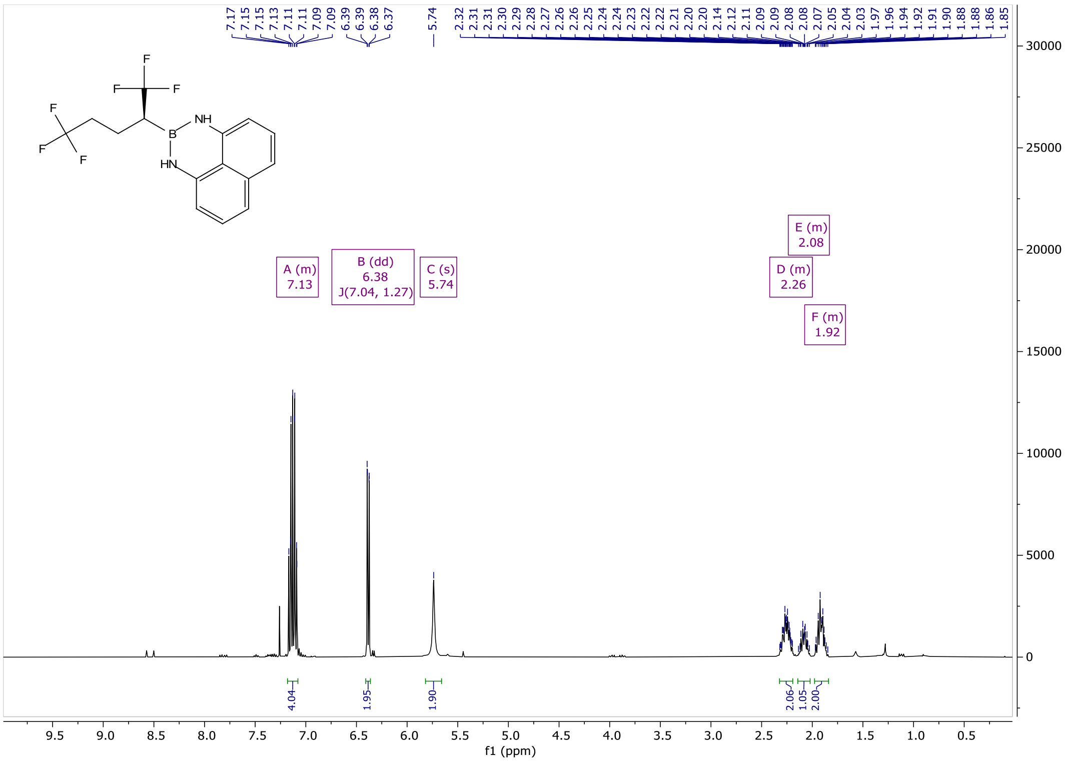

^{13}C NMR ($CDCl_3$, 101 MHz) of compound **5k**

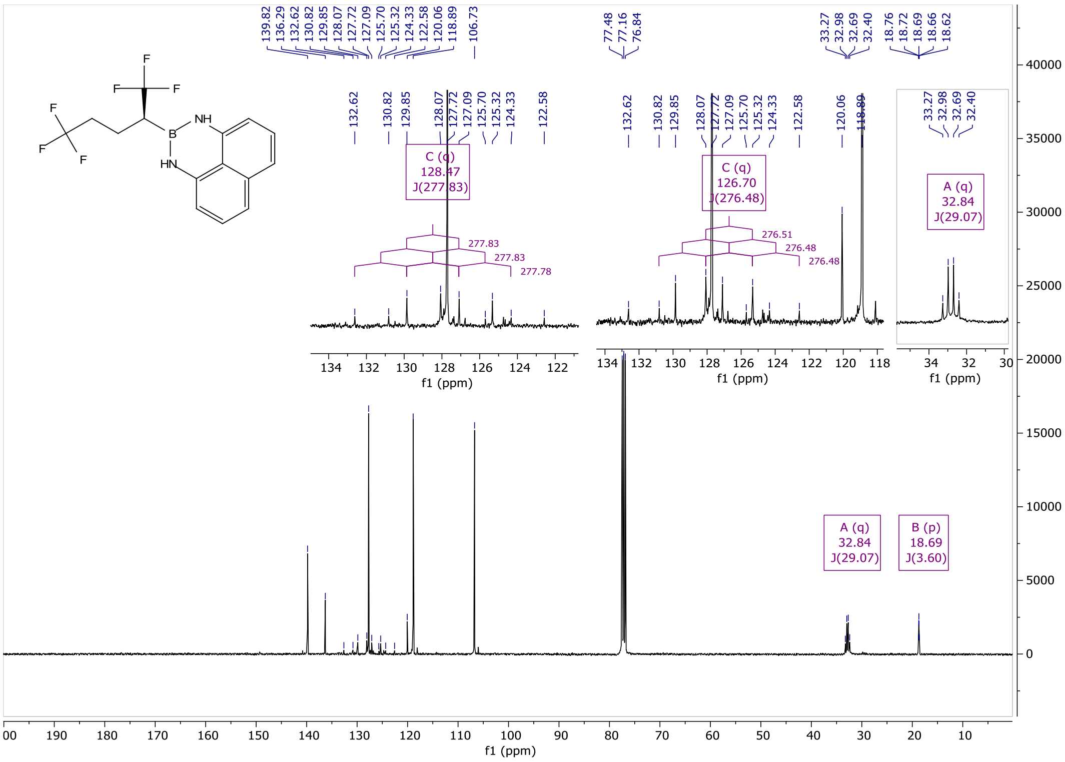

^{19}F NMR ($CDCl_3$, 377 MHz) of compound **5k**

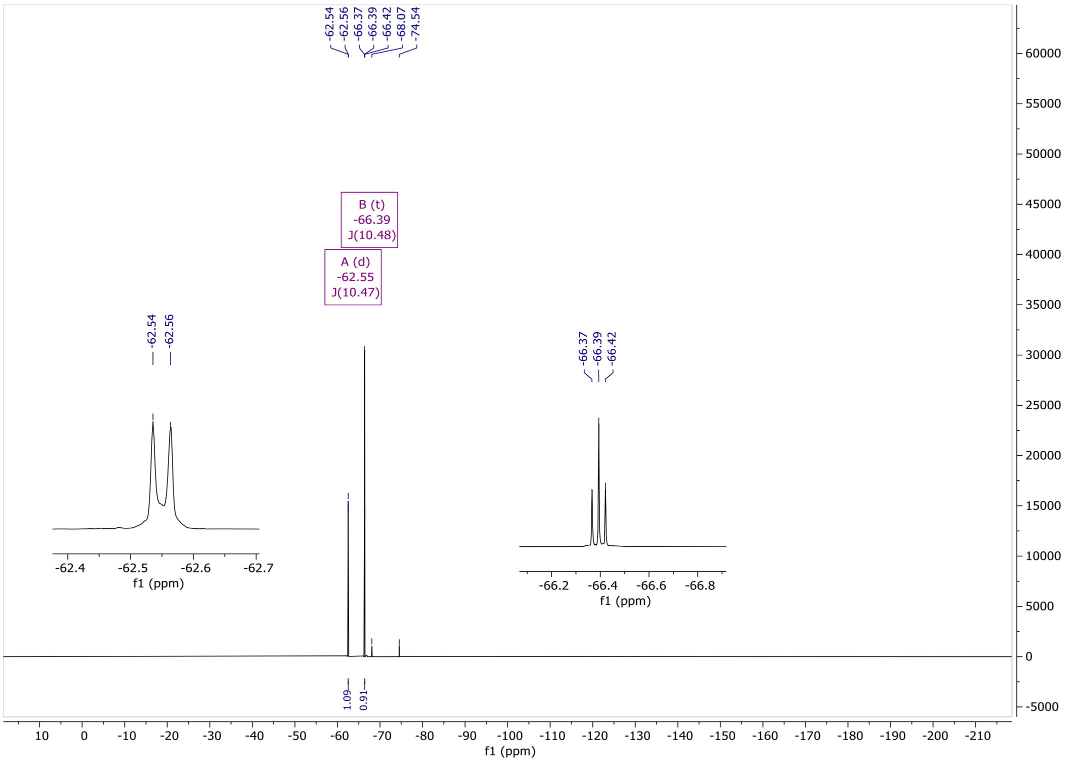

^{11}B NMR ($CDCl_3$, 128 MHz) of compound **5k**

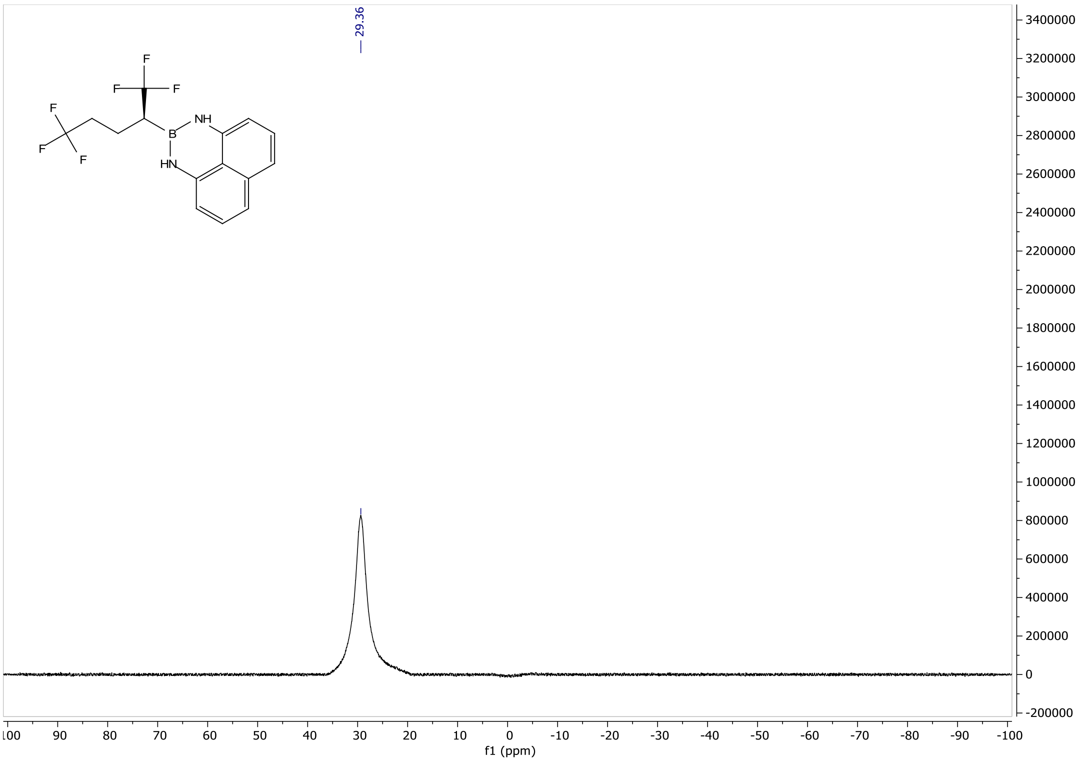

^{1}H NMR ($CDCl_3$, 400 MHz) of compound **5l**

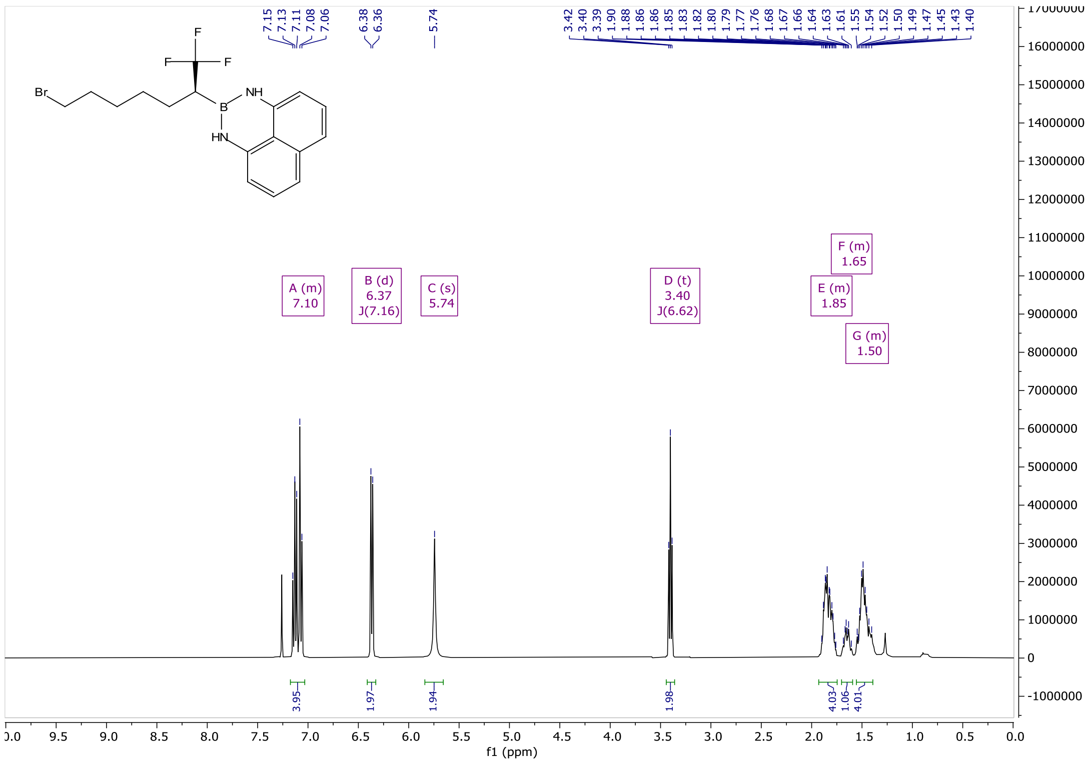

^{13}C NMR ($CDCl_3$, 101 MHz) of compound **5l**

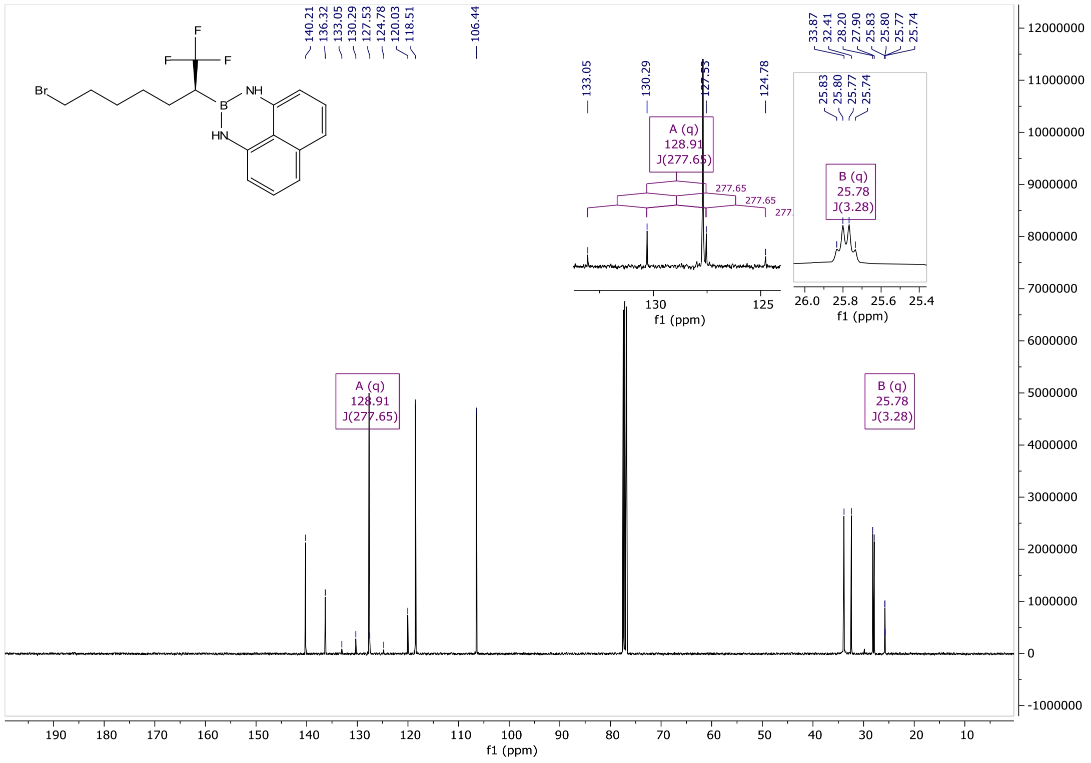

^{19}F NMR ($CDCl_3$, 377 MHz) of compound **5l**

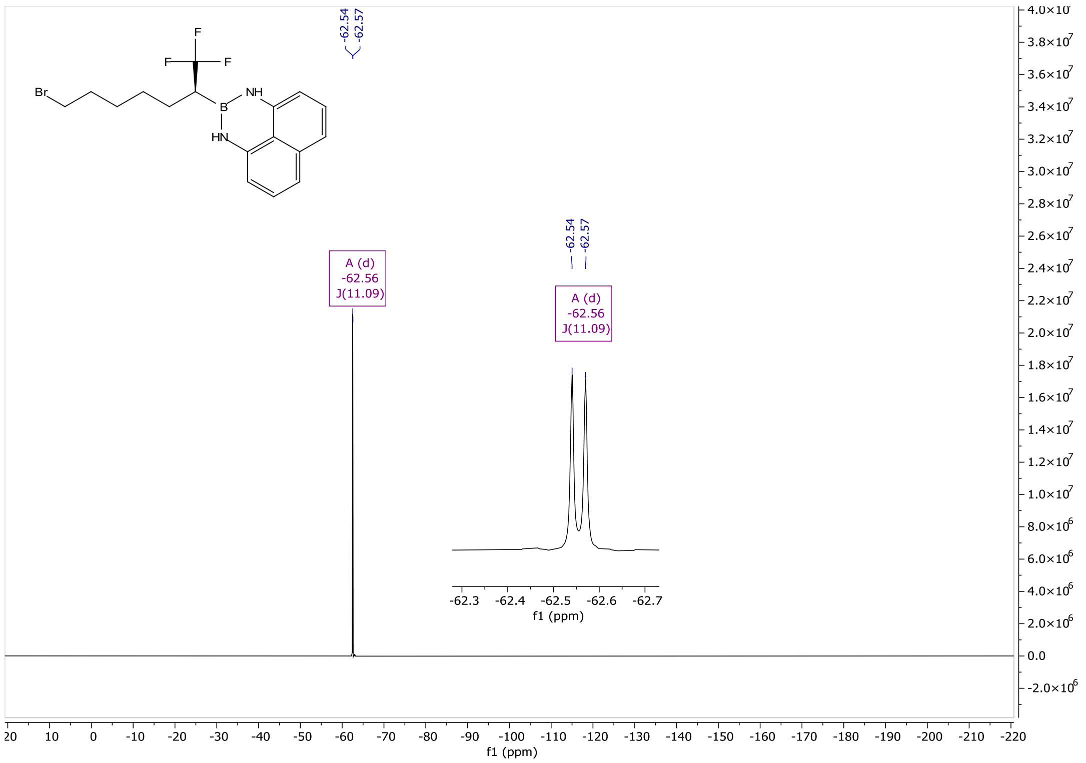

^{11}B NMR ($CDCl_3$, 128 MHz) of compound **5l**

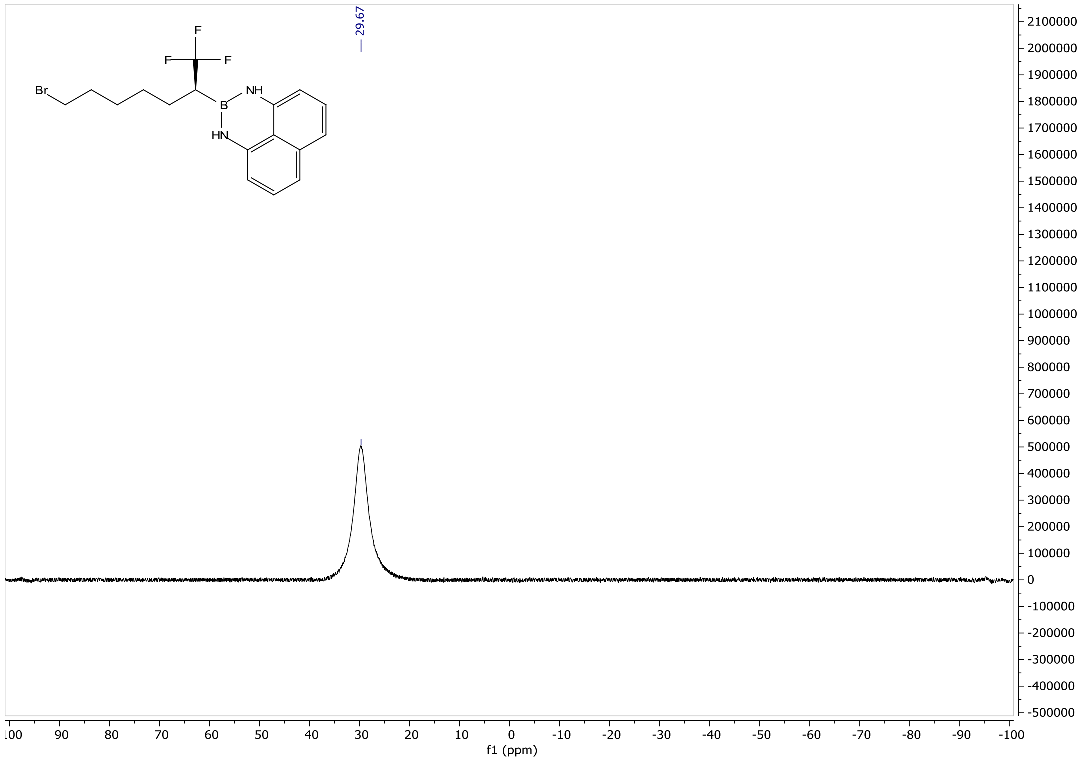

^{1}H NMR ($CDCl_3$, 400 MHz) of compound **5m**

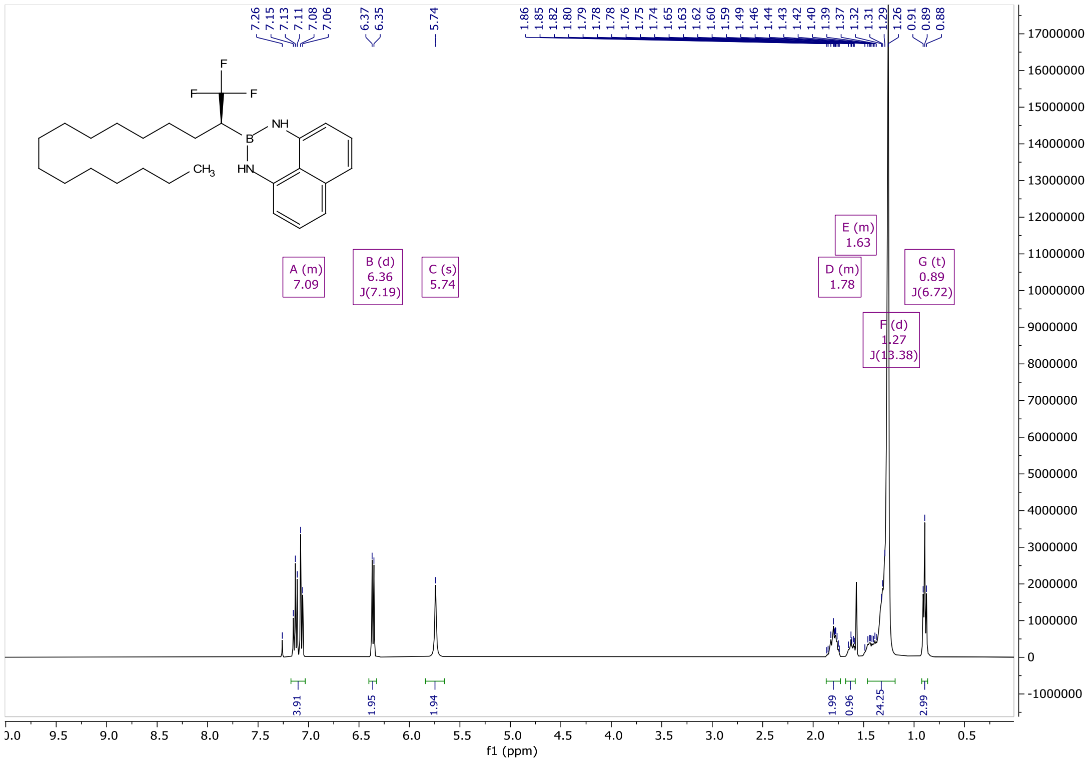

^{13}C NMR ($CDCl_3$, 101 MHz) of compound **5m**

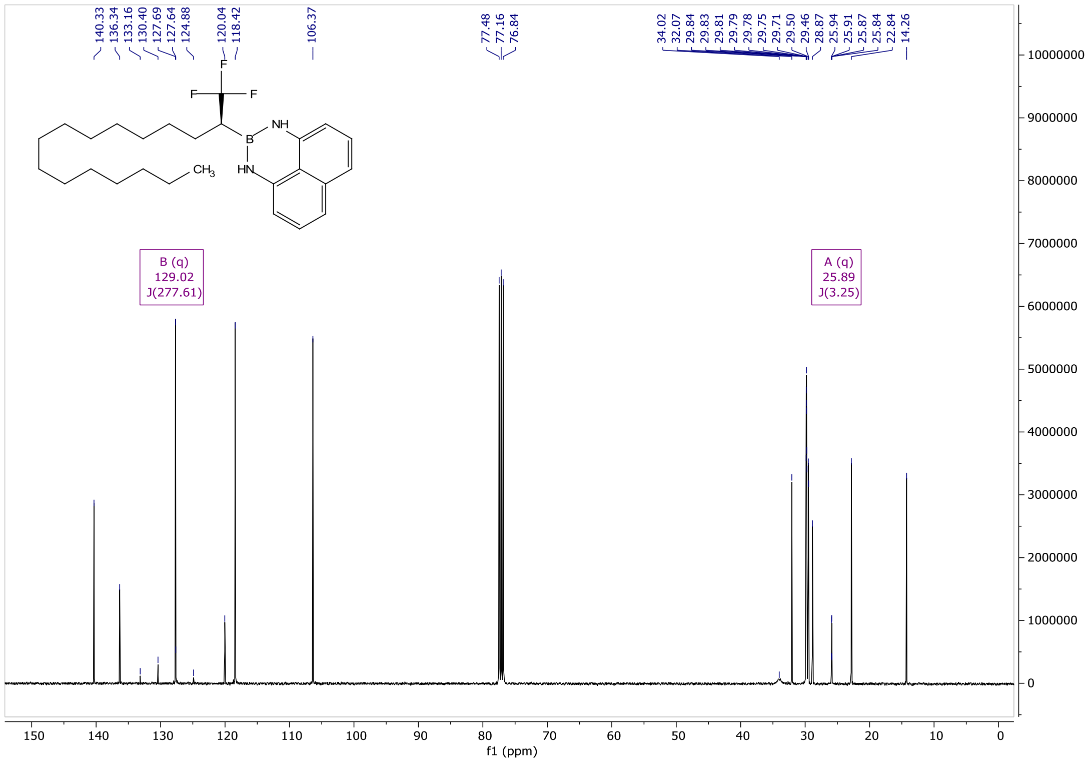

^{19}F NMR ($CDCl_3$, 377 MHz) of compound **5m**

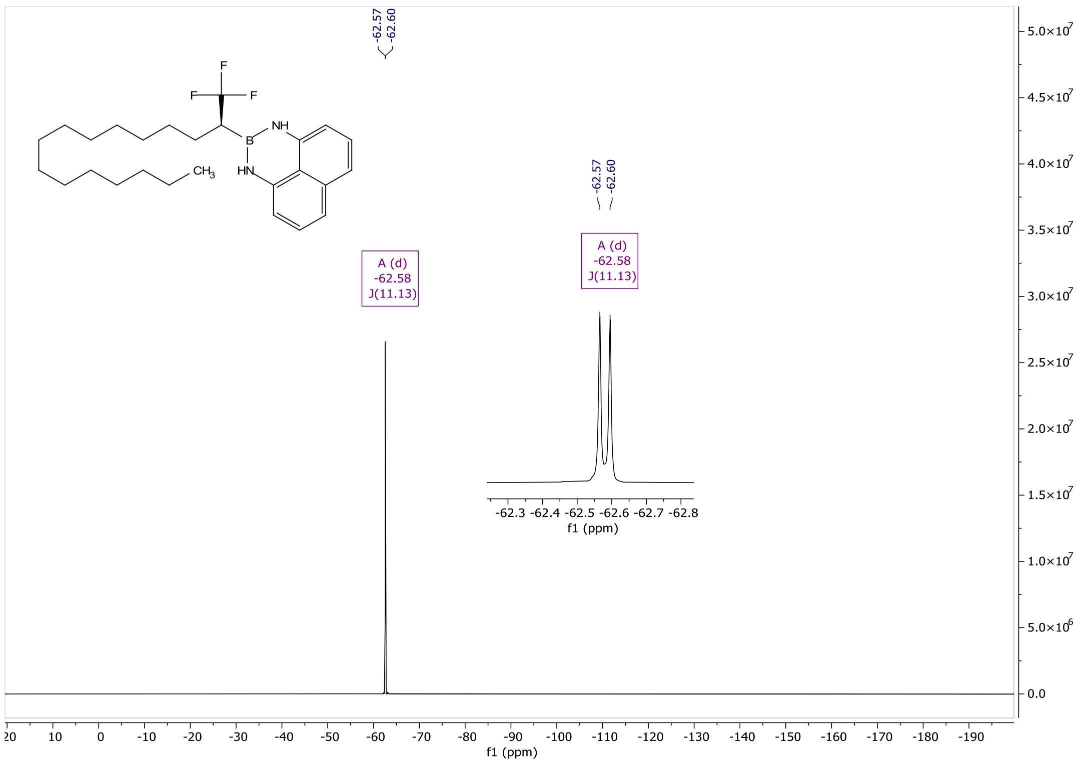

^{11}B NMR ($CDCl_3$, 128 MHz) of compound **5m**

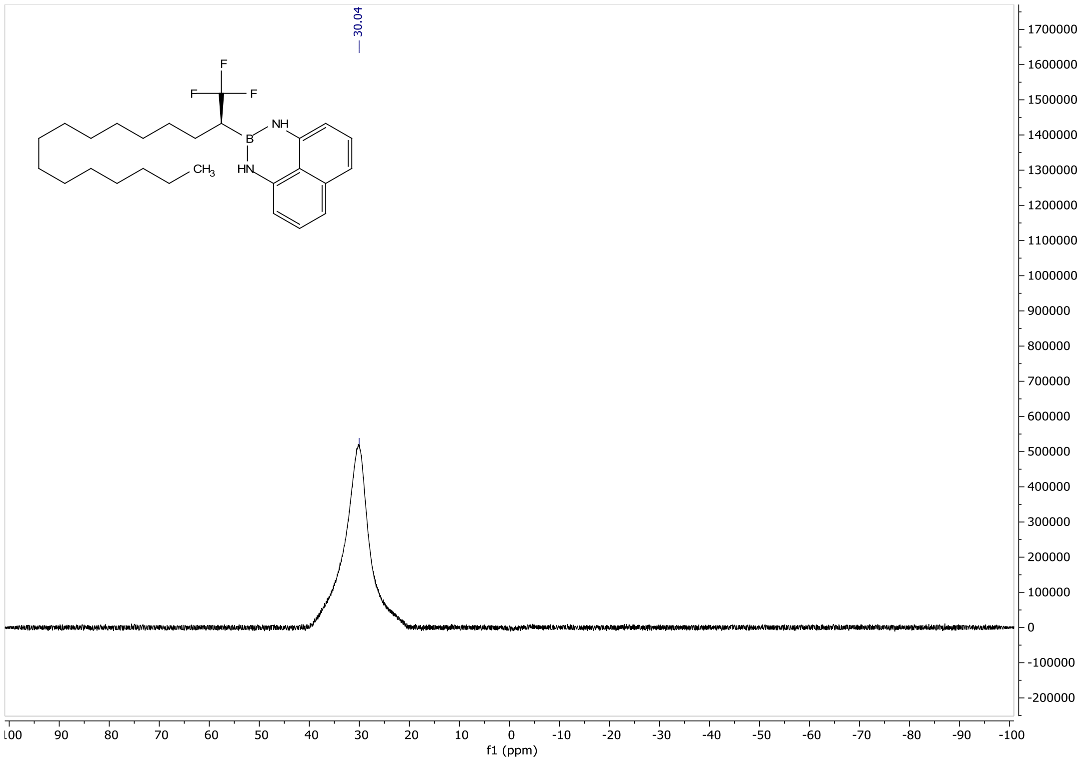

^{1}H NMR ($CDCl_3$, 400 MHz) of compound **6**

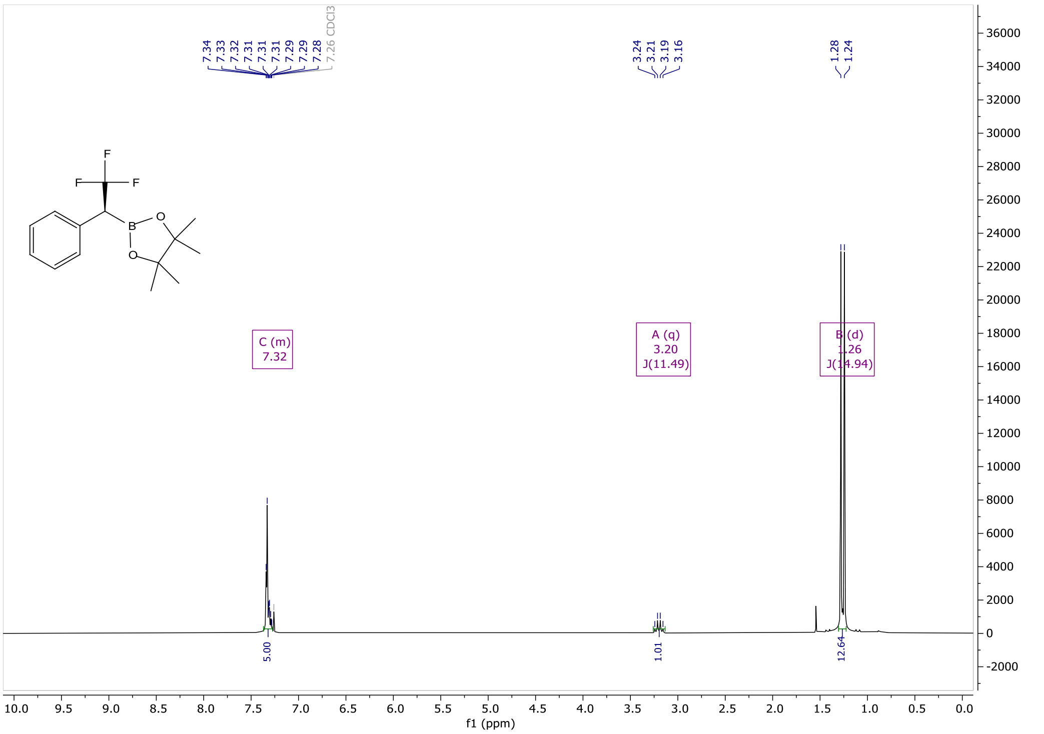

^{13}C NMR ($CDCl_3$, 101 MHz) of compound **6**

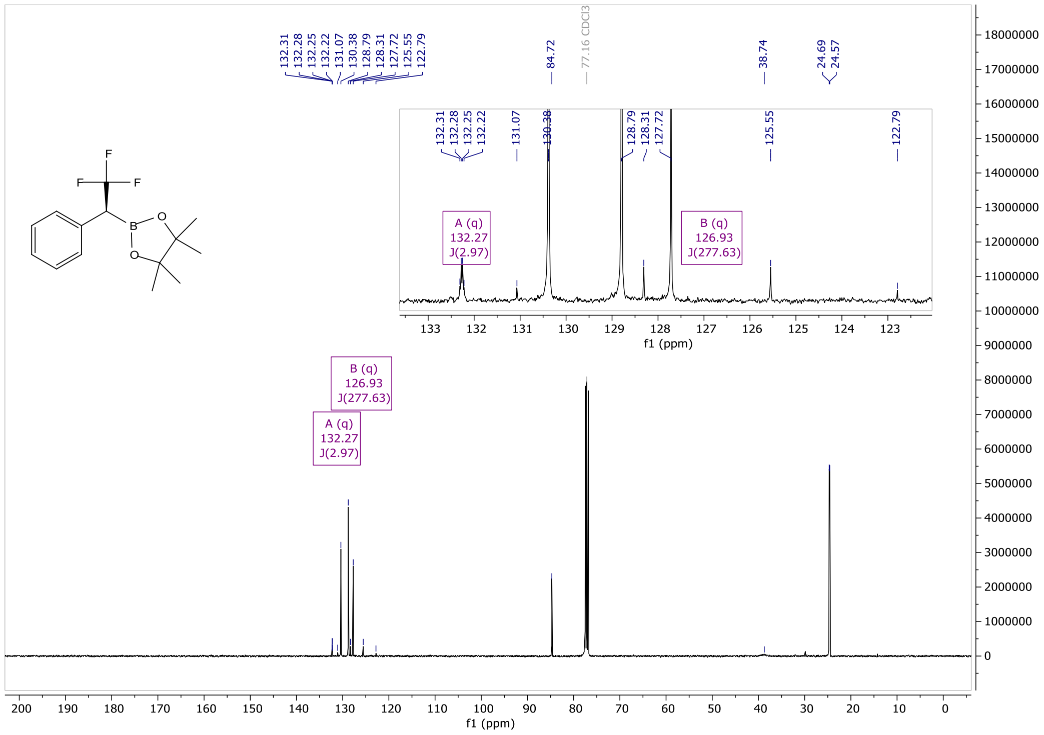

^{19}F NMR ($CDCl_3$, 377 MHz) of compound **6**

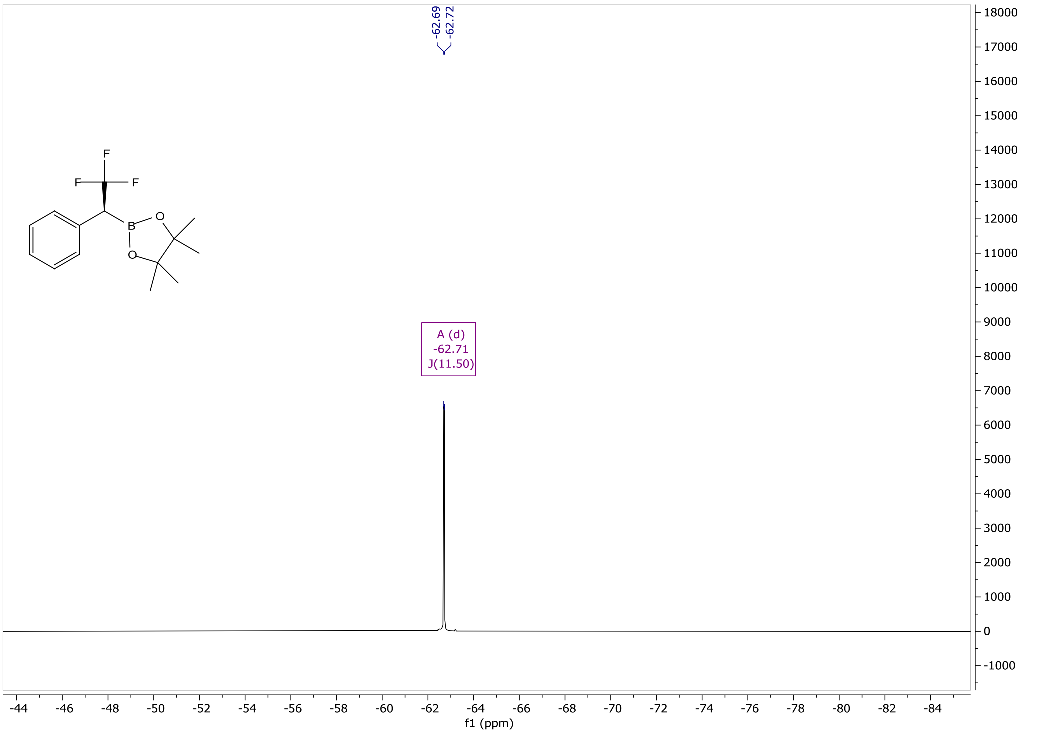

^{11}B NMR ($CDCl_3$, 128 MHz) of compound **6**

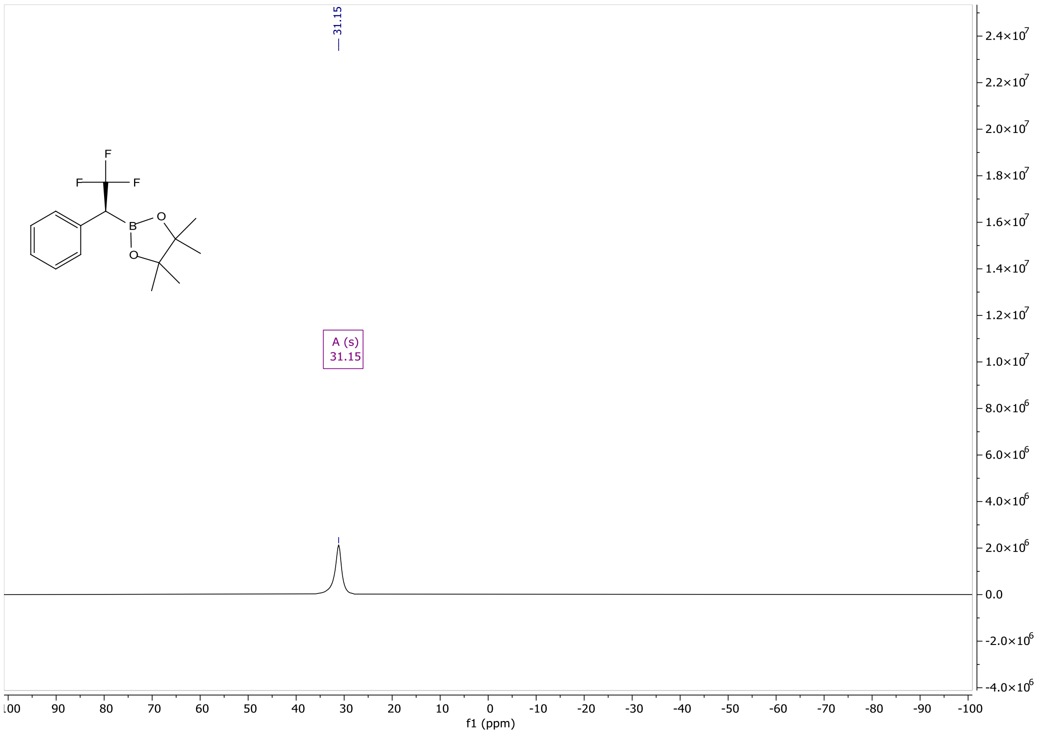

^{1}H NMR ($CDCl_3$, 400 MHz) of compound **7**

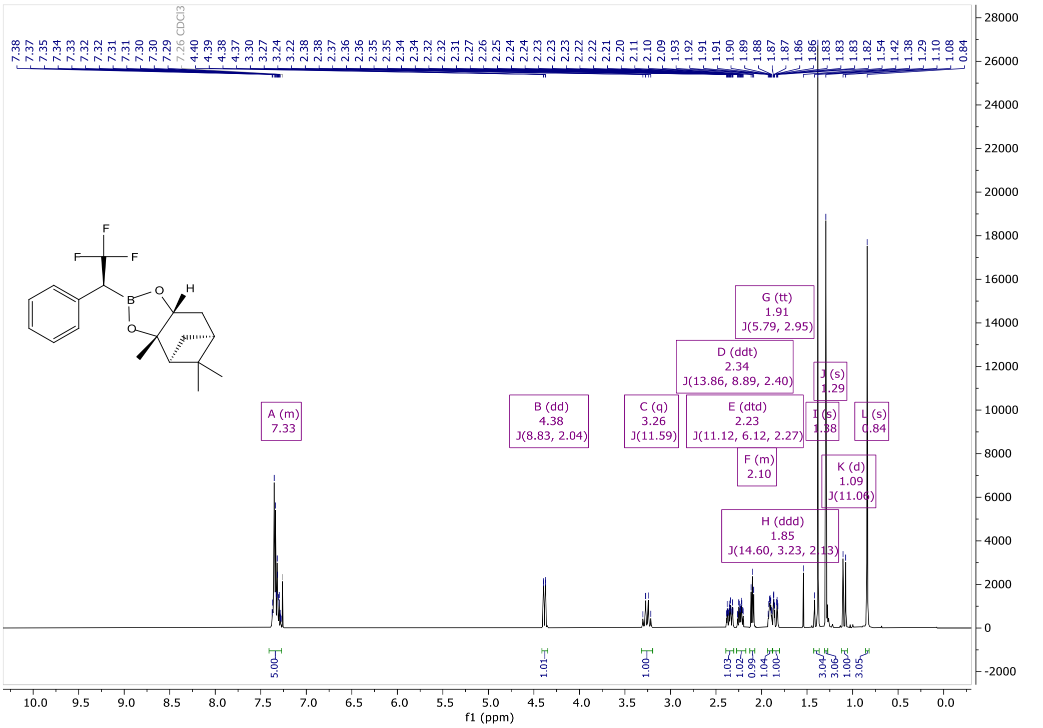

^{13}C NMR ($CDCl_3$, 101 MHz) of compound **7**

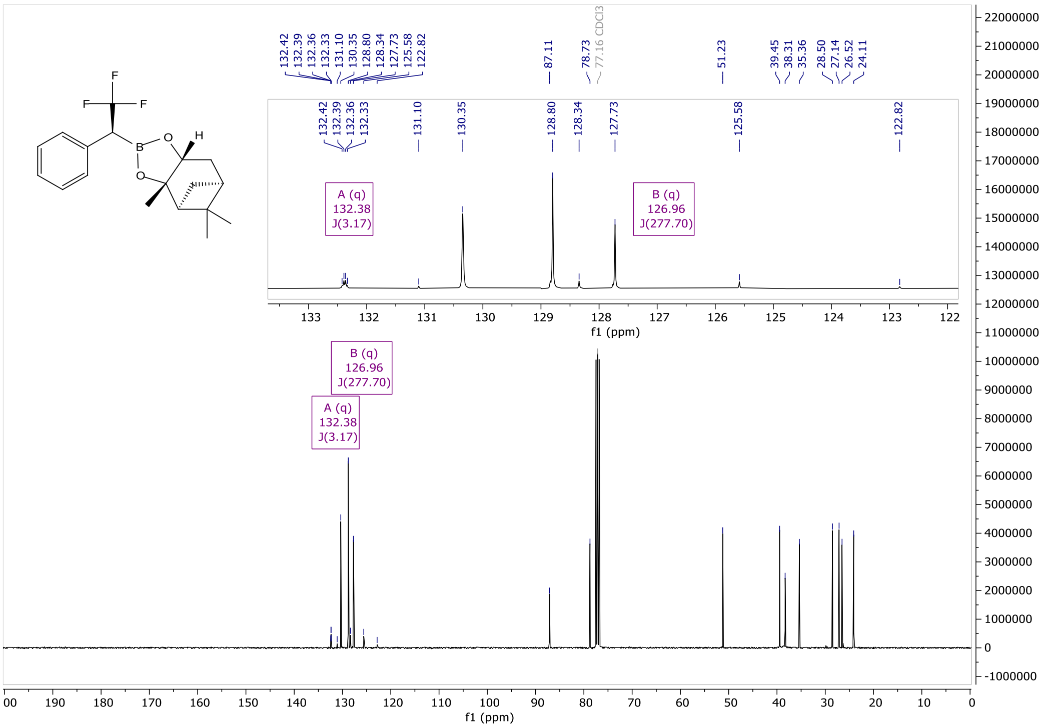

^{19}F NMR ($CDCl_3$, 377 MHz) of compound **7**

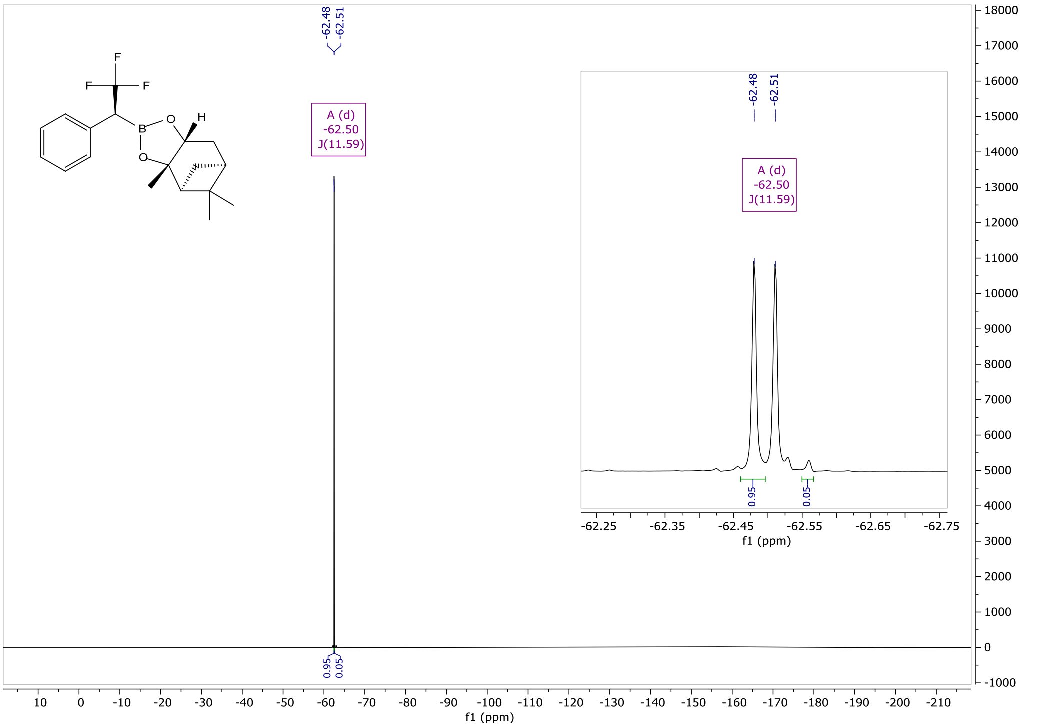

^{11}B NMR ($CDCl_3$, 128 MHz) of compound **7**

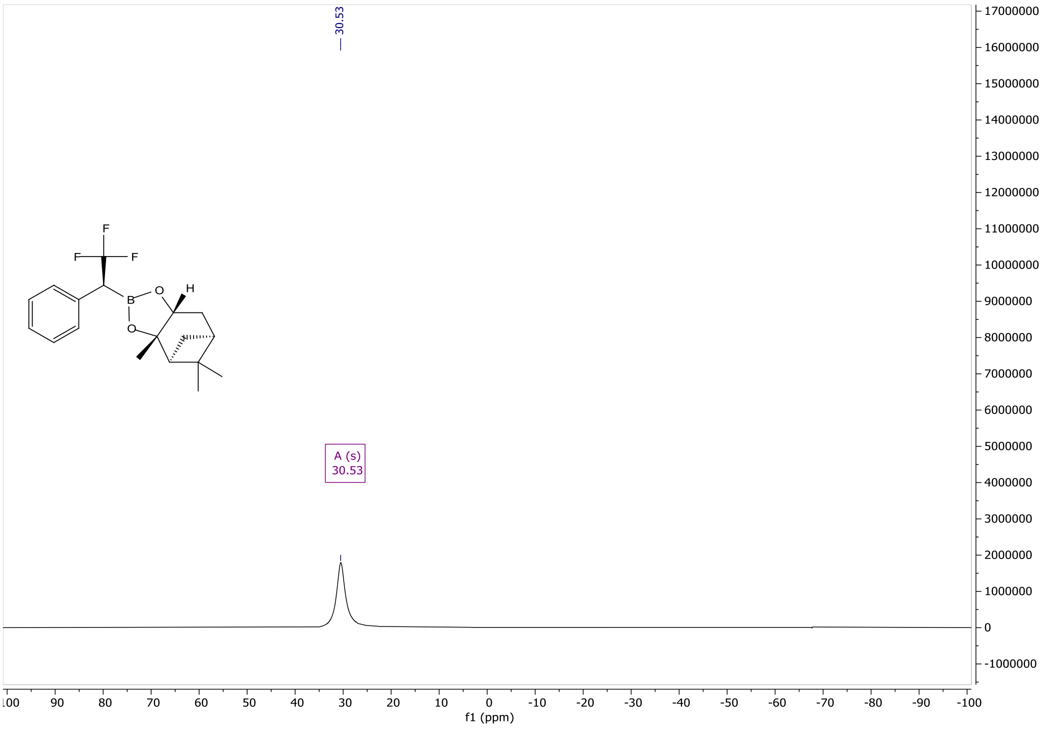

^1H NMR ($CDCl_3$, 400 MHz) of compound **8a**

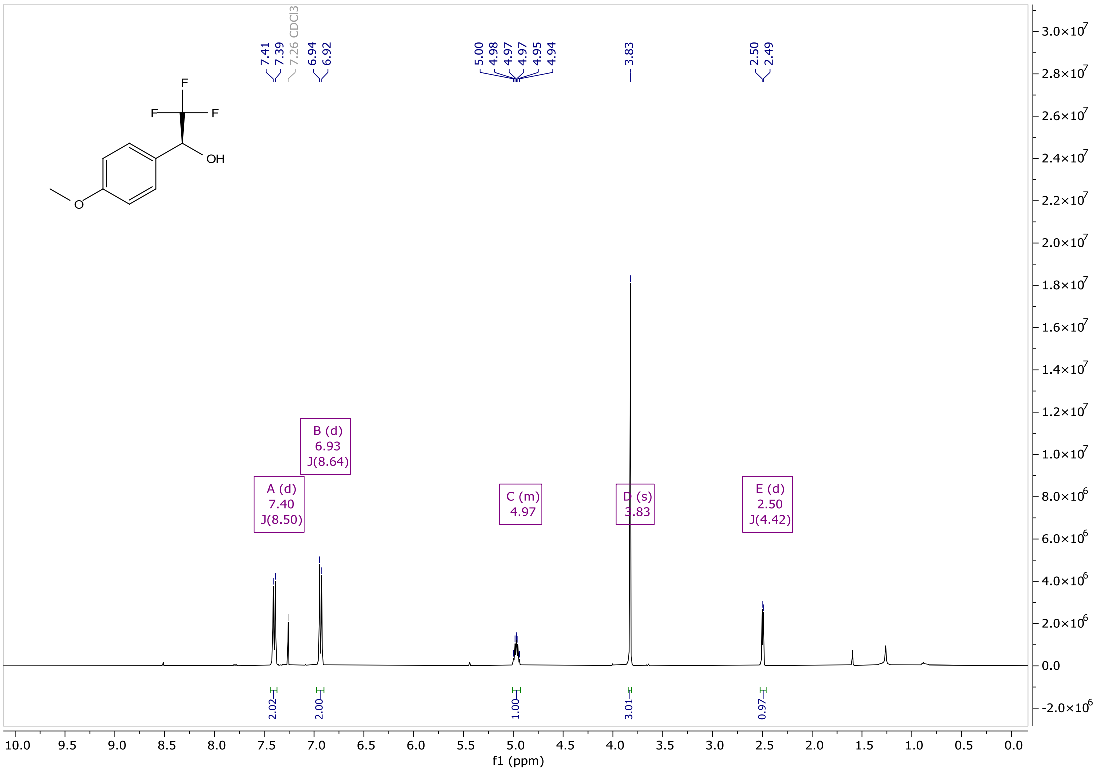

^{13}C NMR ($CDCl_3$, 101 MHz) of compound **8a**

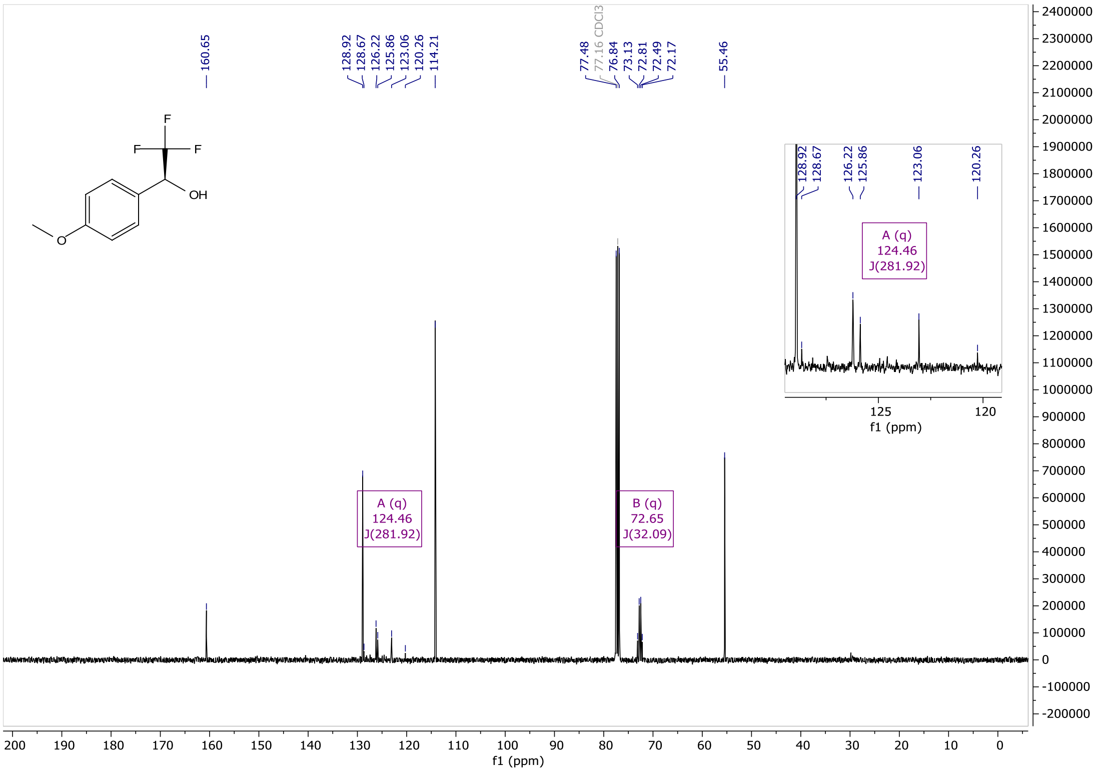

^{19}F NMR ($CDCl_3$, 377 MHz) of compound **8a**

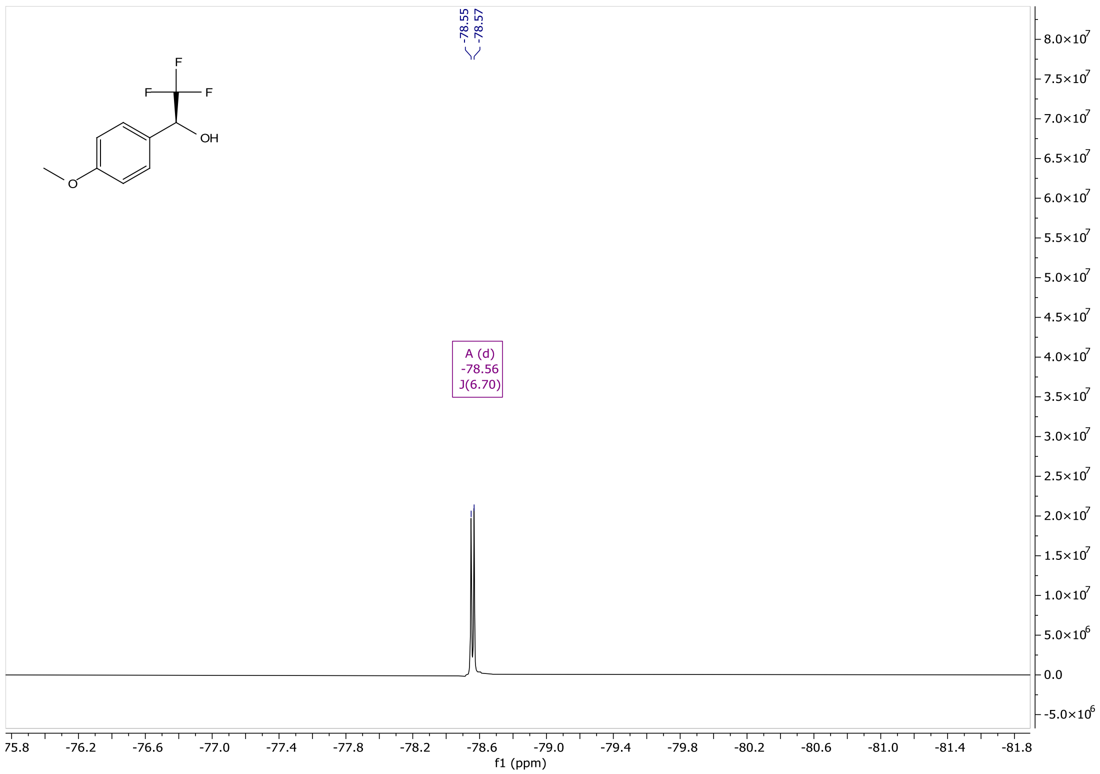

^{1}H NMR ($CDCl_3$, 400 MHz) of compound **8b**

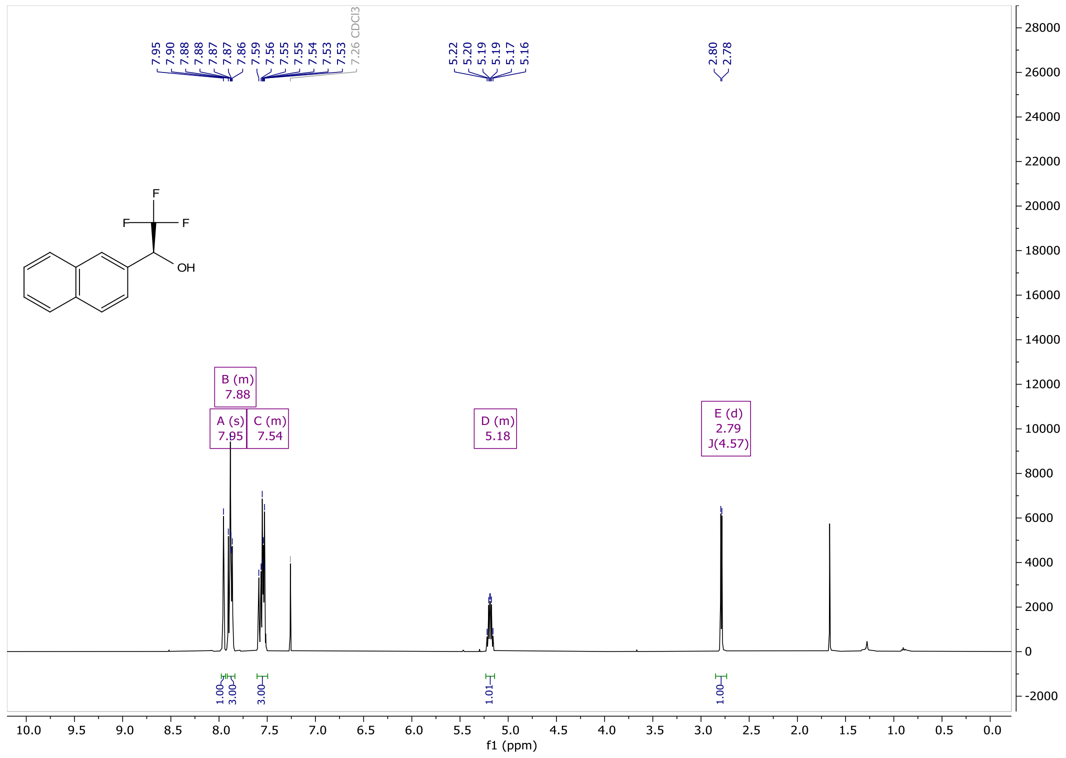

^{13}C NMR ($CDCl_3$, 101 MHz) of compound **8b**

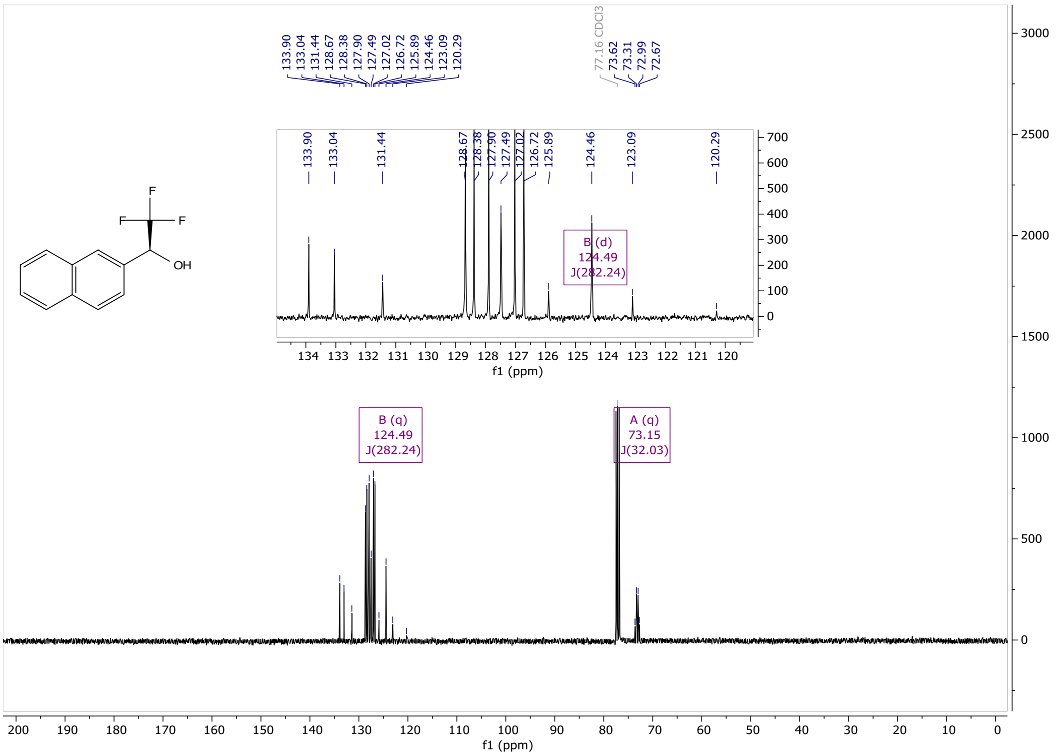

^{19}F NMR ($CDCl_3$, 377 MHz) of compound **8b**

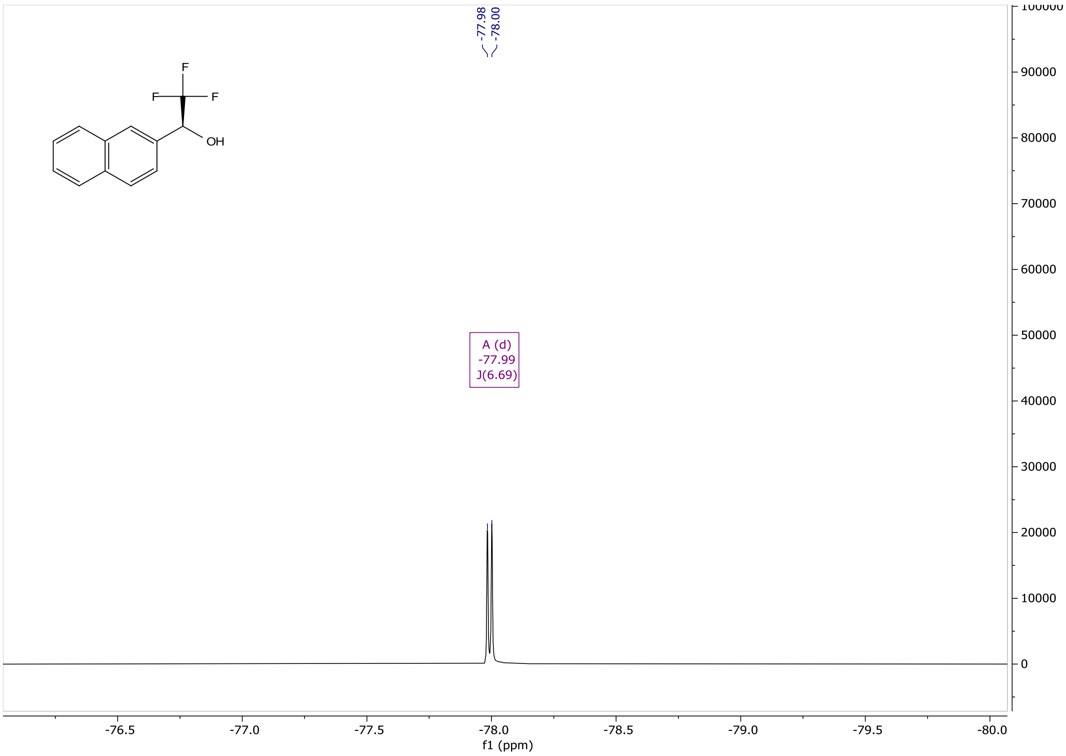

^{1}H NMR ($CDCl_3$, 400 MHz) of compound **8c**

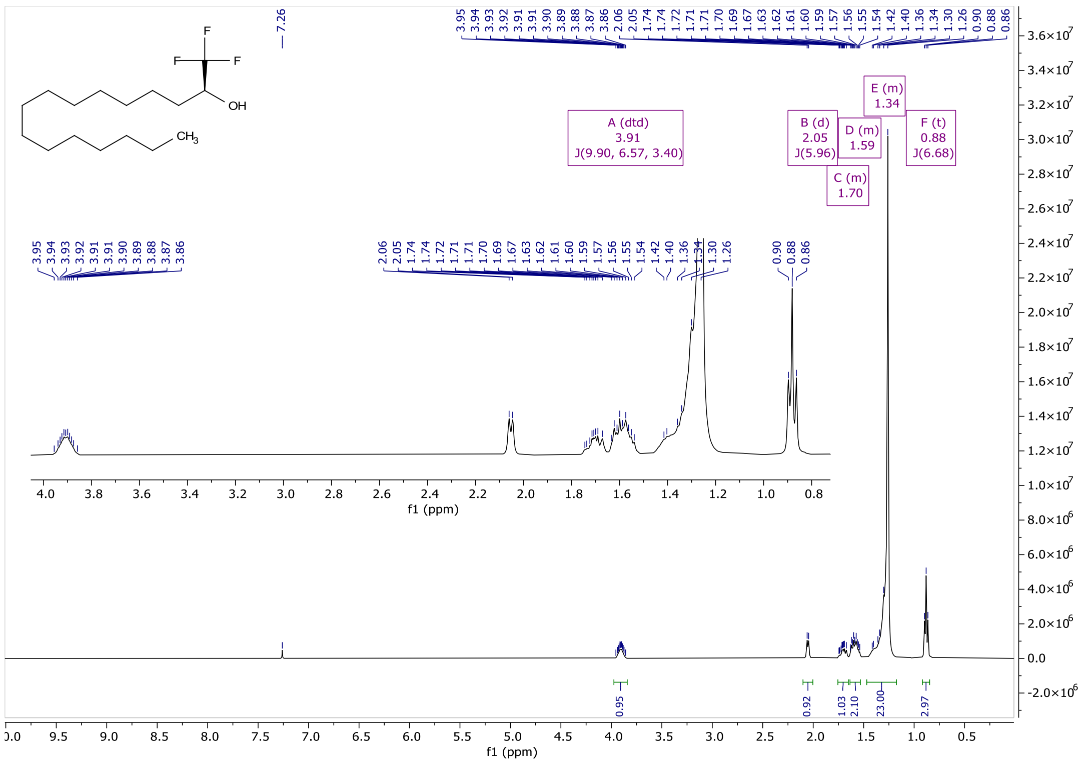

[3]C NMR ($CDCl_3$, 101 MHz) of compound **8c**

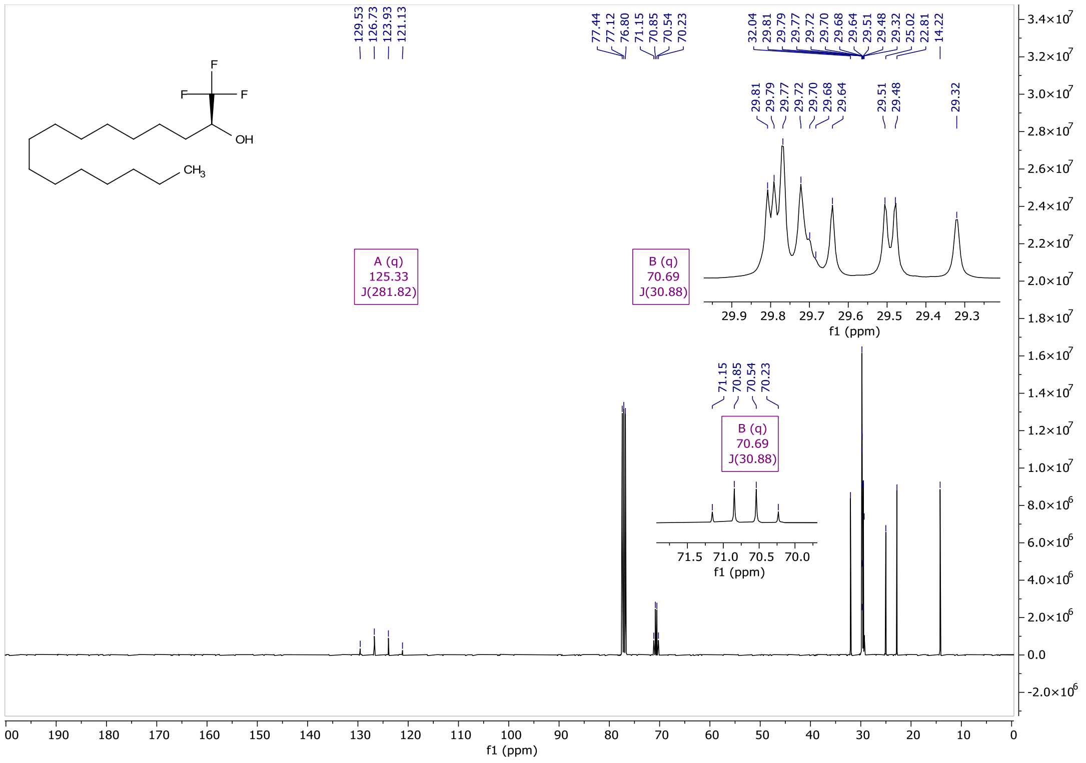

^{19}F NMR ($CDCl_3$, 377 MHz) of compound **8c**

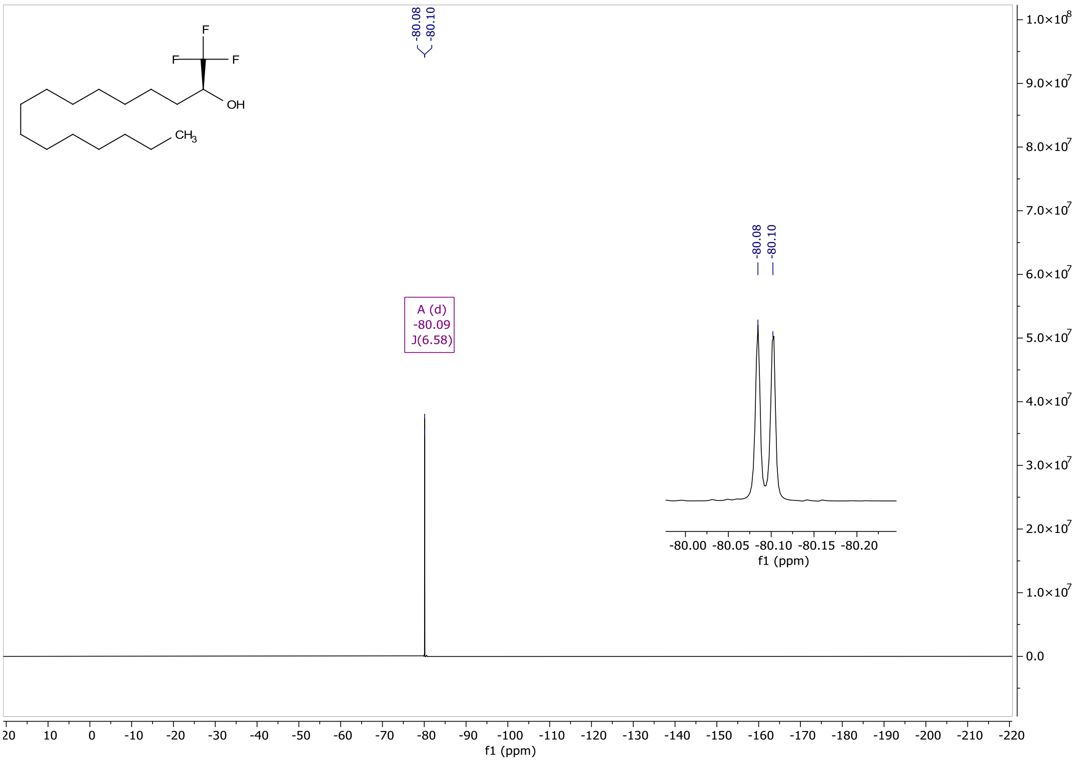

^{1}H NMR ($CDCl_3$, 400 MHz) of compound **8c-1**

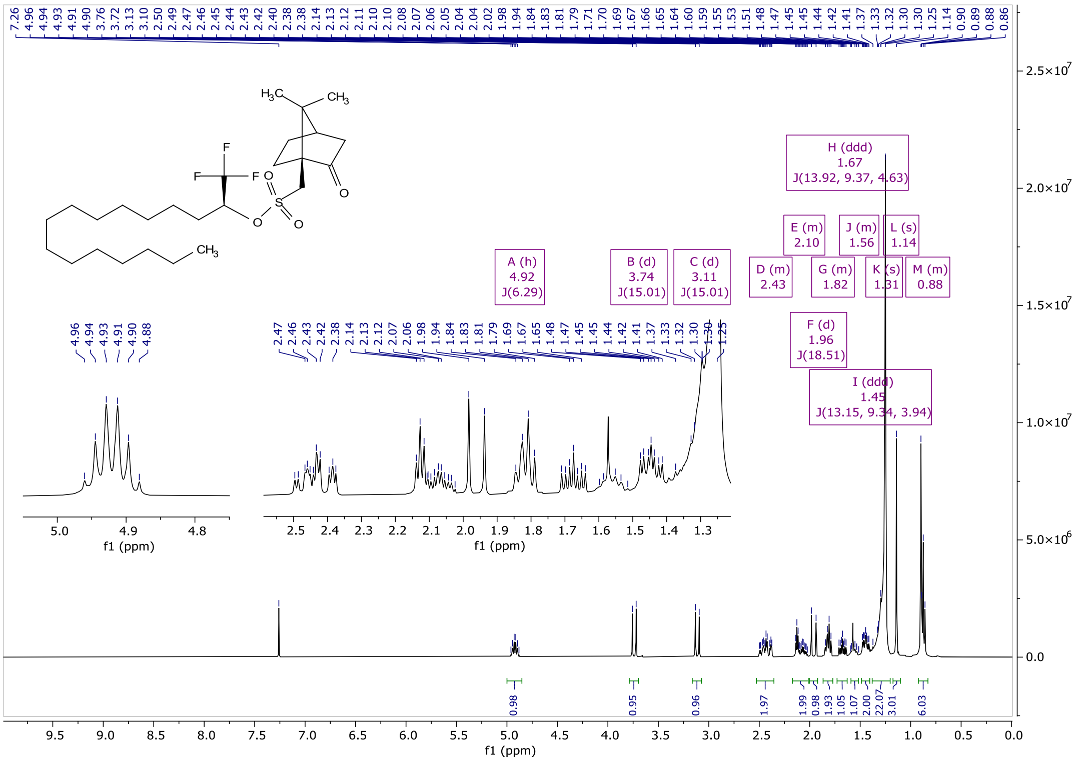

^{13}C NMR ($CDCl_3$, 101 MHz) of compound **8c-1**

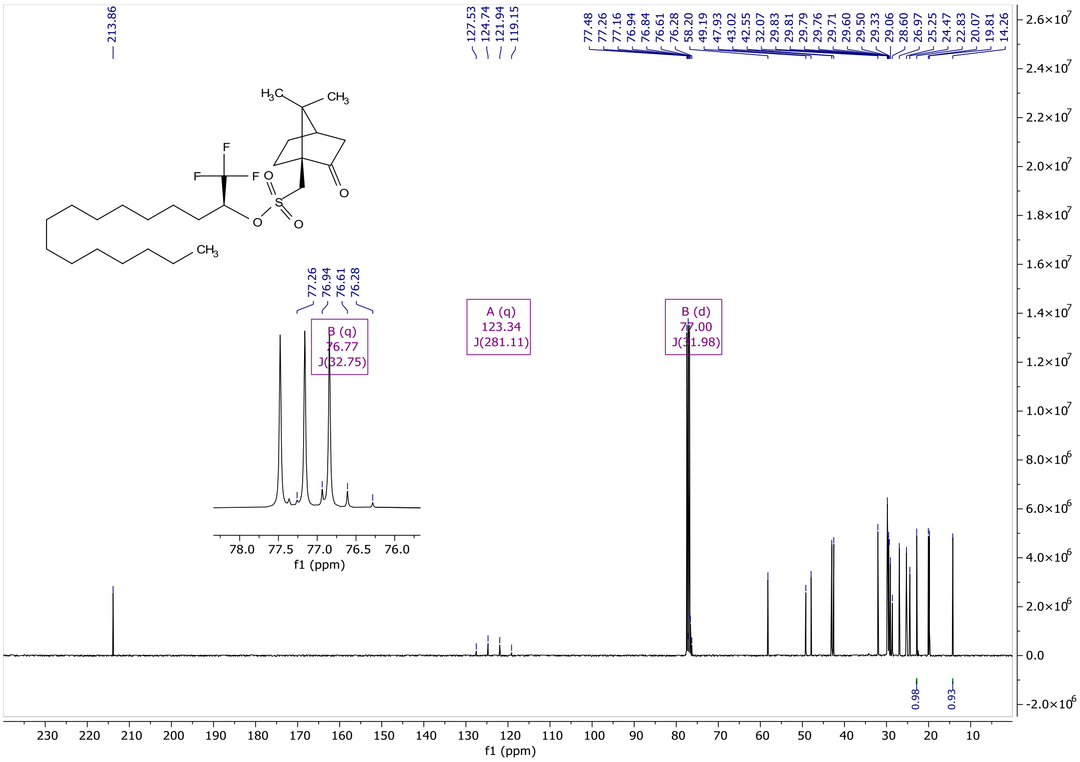

^{19}F NMR ($CDCl_3$, 377 MHz) of compound **8c-1**

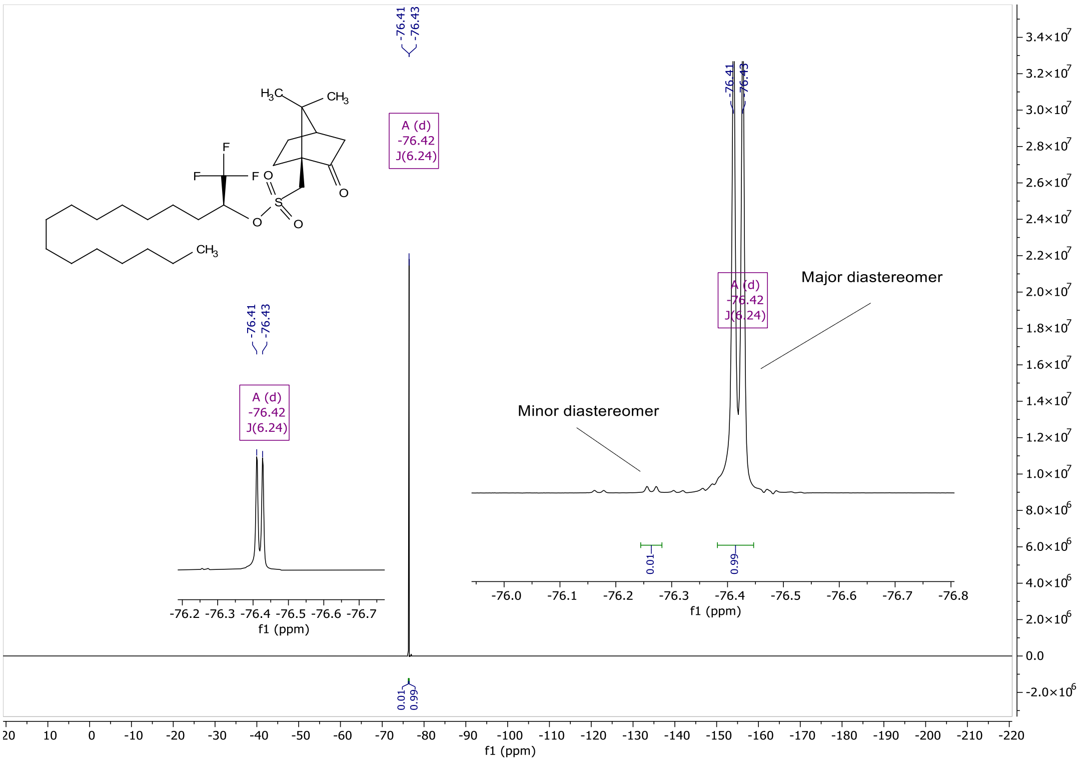

^{1}H NMR (CDCl3, 400 MHz) of compound **9a**

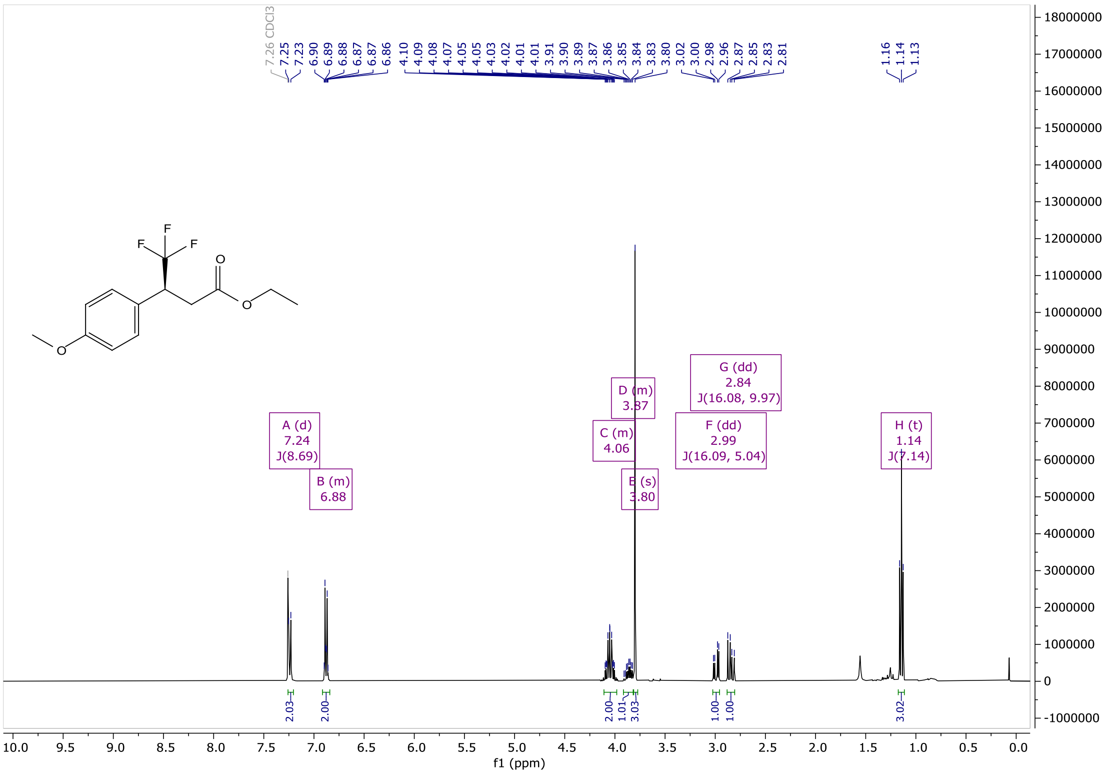

^{13}C NMR ($CDCl_3$, 101 MHz) of compound **9a**

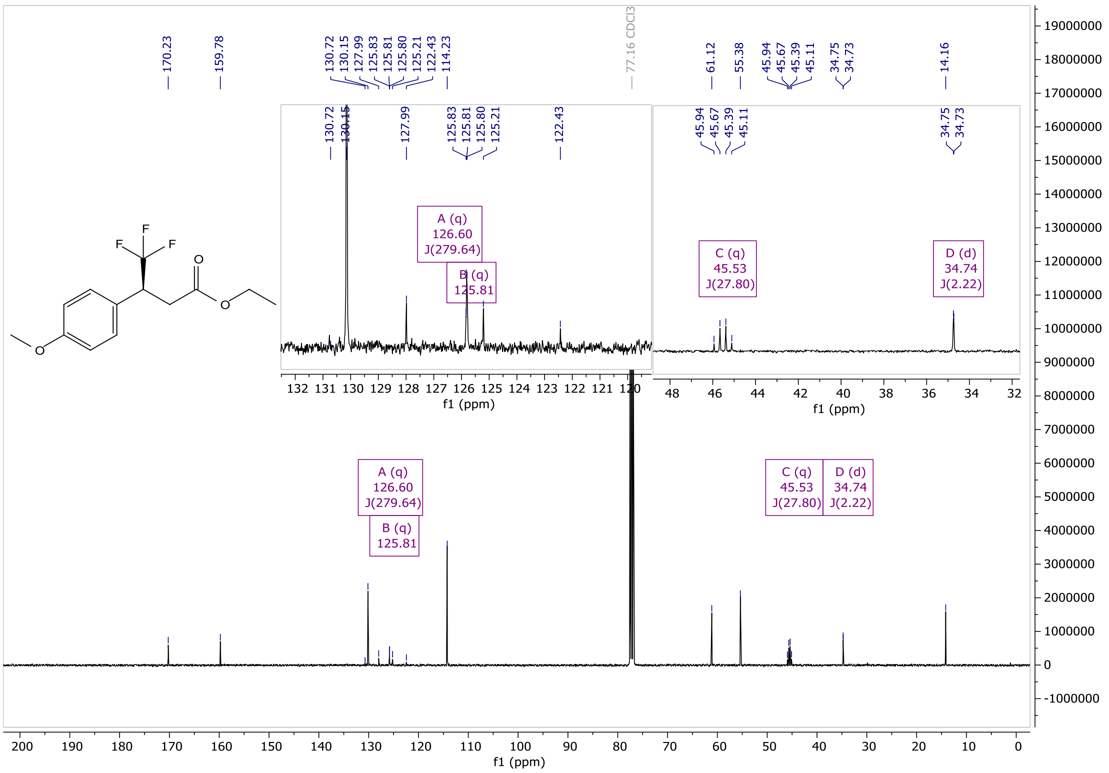

^{19}F NMR ($CDCl_3$, 377 MHz) of compound **9a**

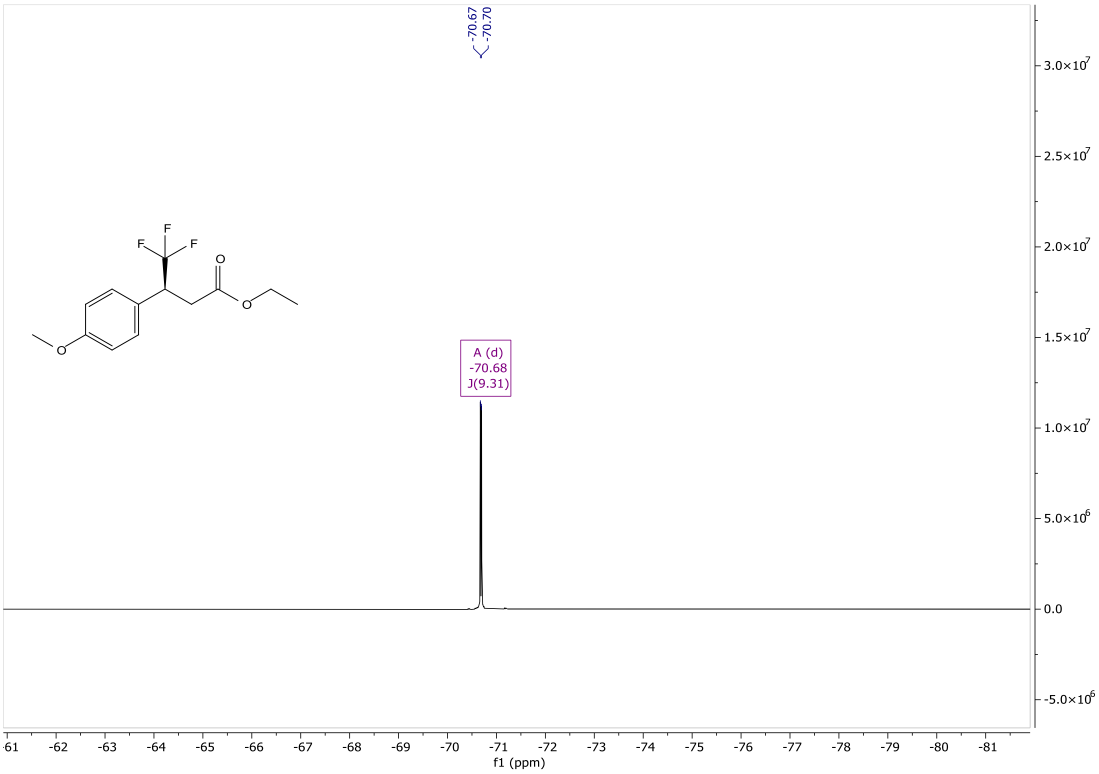

^{1}H NMR ($CDCl_3$, 400 MHz) of compound **9b**

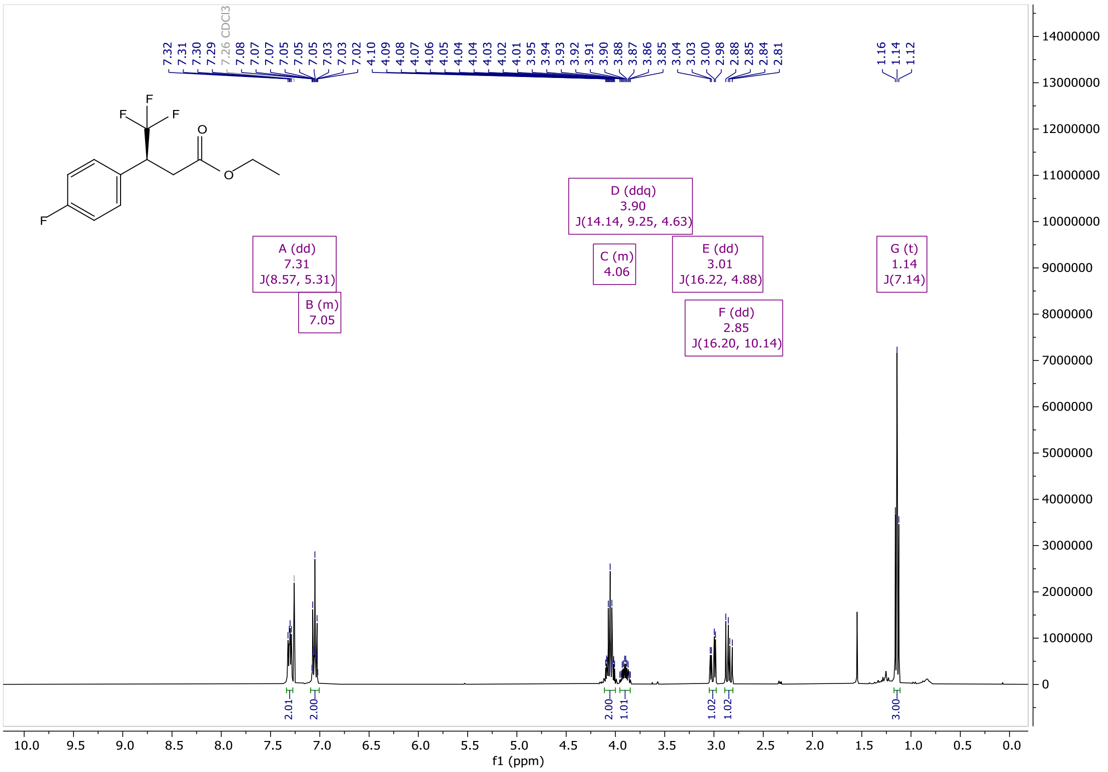

^{13}C NMR ($CDCl_3$, 101 MHz) of compound **9b**

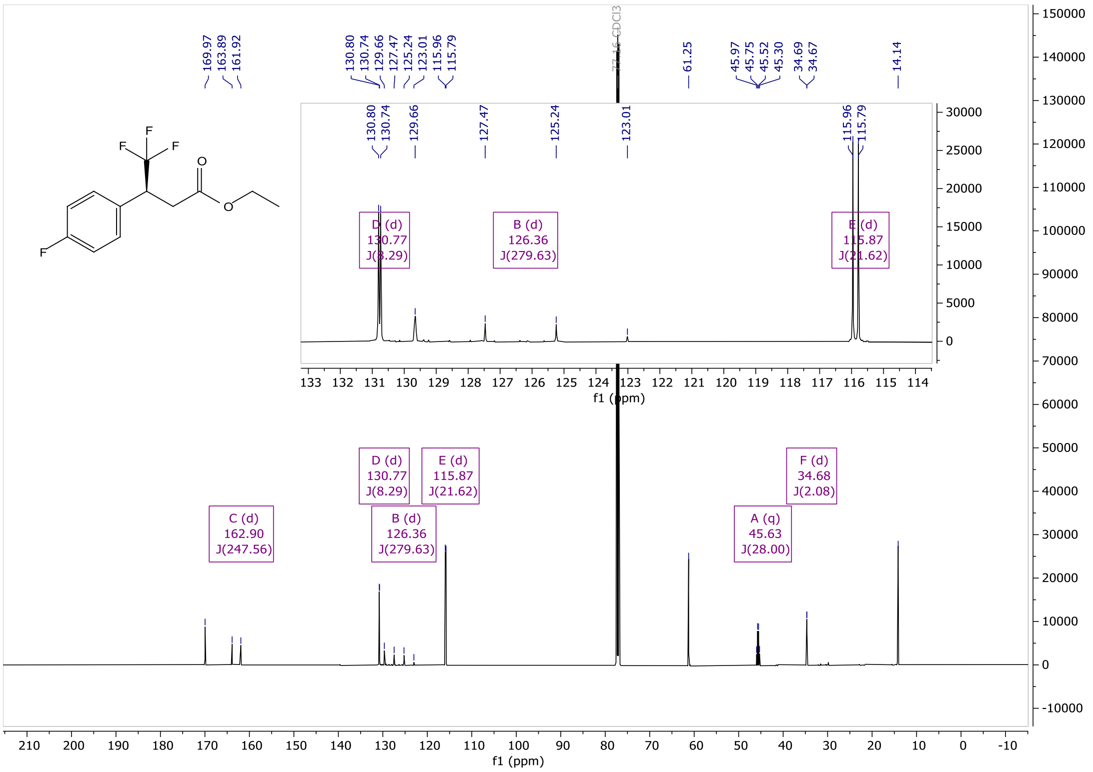

^{19}F NMR ($CDCl_3$, 377 MHz) of compound **9b**

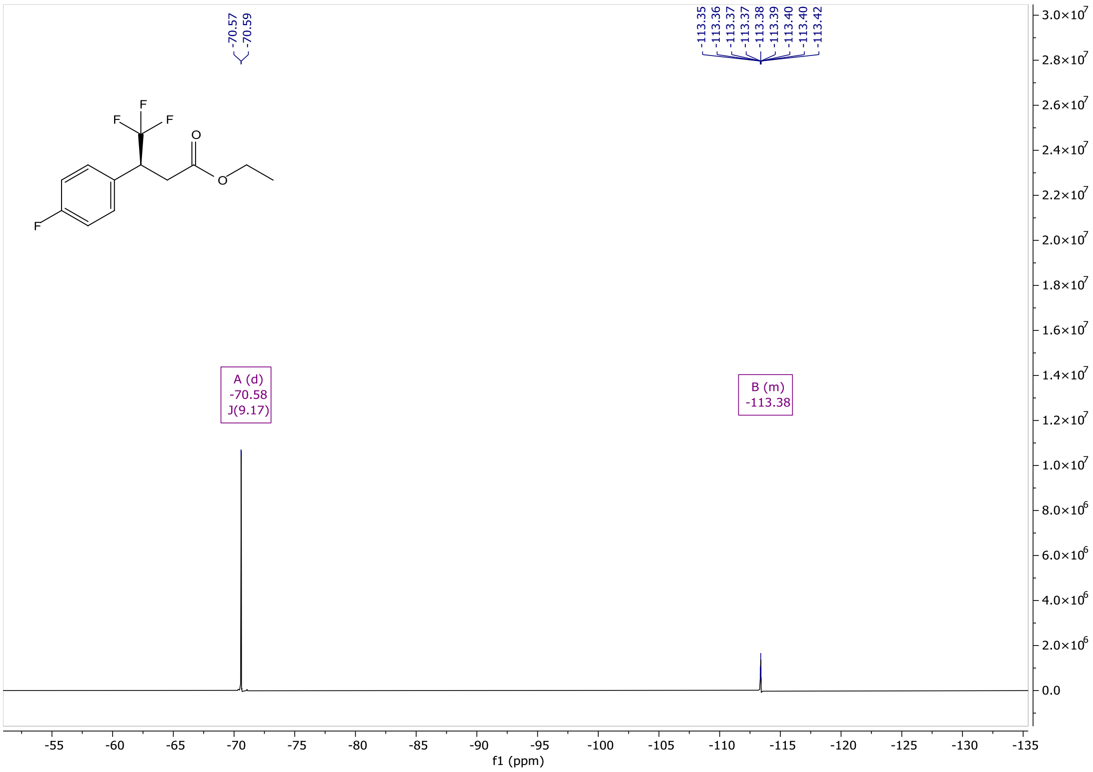

^{1}H NMR ($CDCl_3$, 400 MHz) of compound **9c**

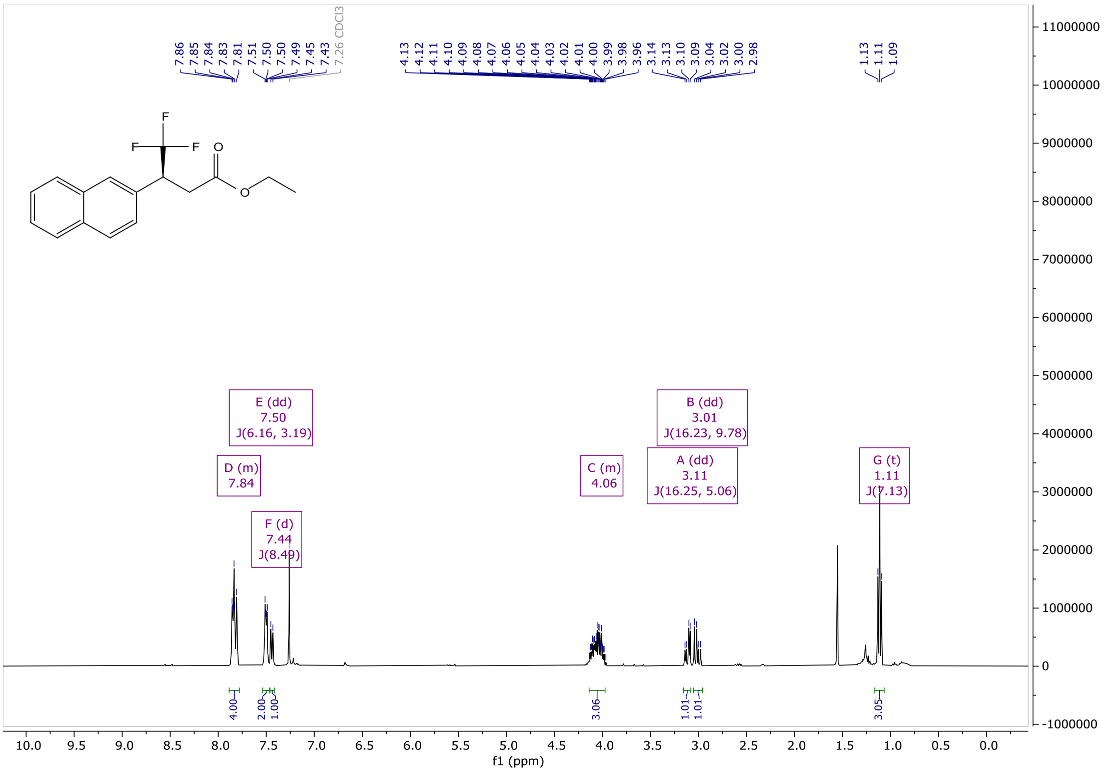

^{13}C NMR ($CDCl_3$, 101 MHz) of compound **9c**

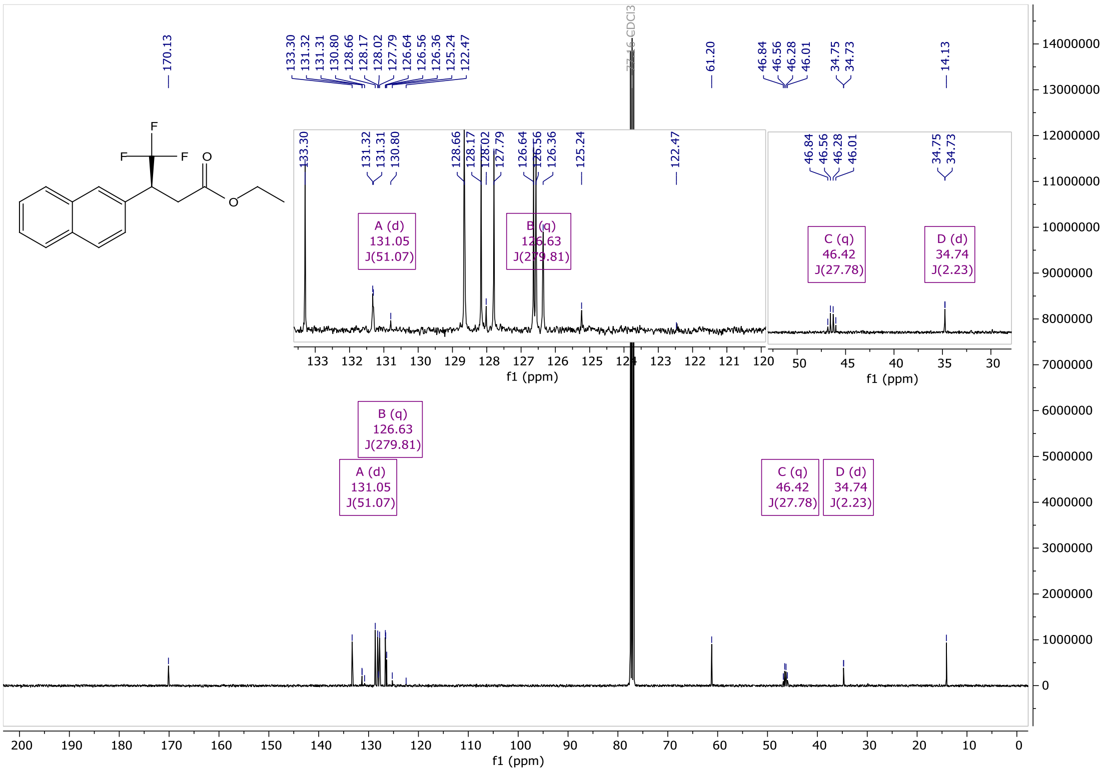

^{19}F NMR ($CDCl_3$, 377 MHz) of compound **9c**

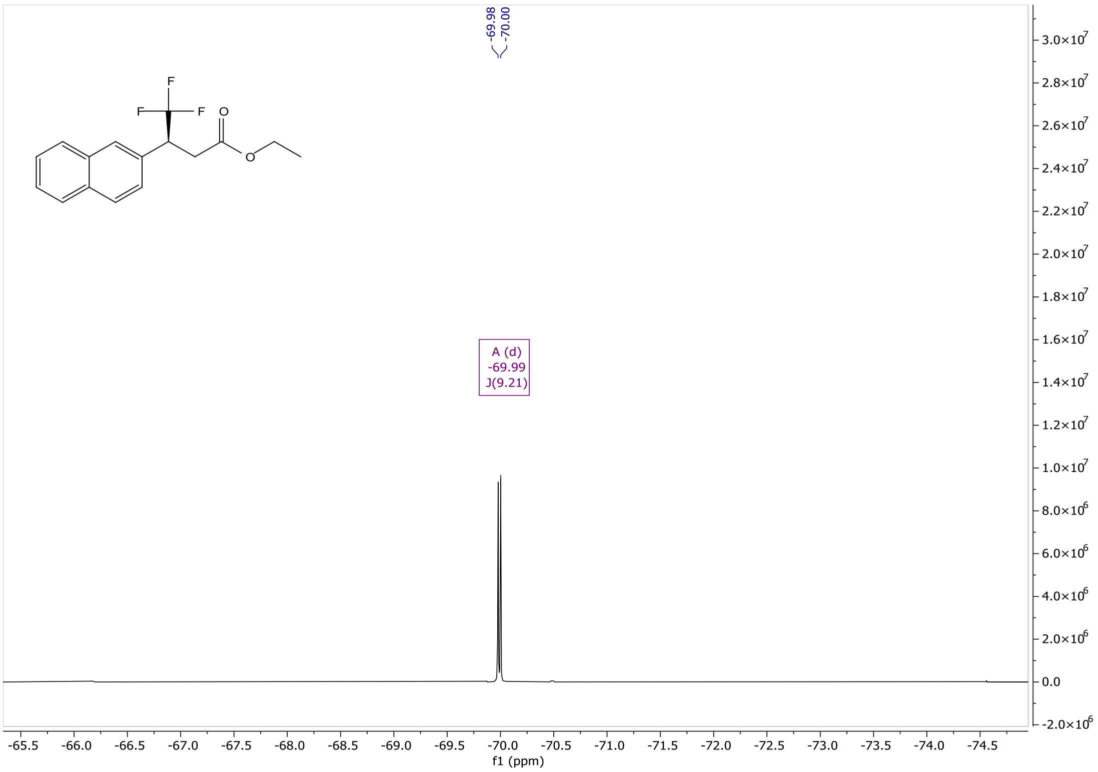

4. References

[1] R. R. Milburn, S. M. S. Hussain, O. Prien, Z. Ahmed, V. Snieckus, *Org. Lett.* **2007**, *9*, 4403-4406.
[2] H. M. Turner, J. Patel, N. Niljianskul, J. M. Chong, *Org. Lett.* **2011**, *13*, 5796-5799.
[3] O. A. Argintaru, D. Ryu, I. Aron, G. A. Molander, *Angew. Chem. Int. Ed.* **2013**, *52*, 13656-13660.
[4] T.-Z. Zhu, P.-L. Shao, X. Zhang, *Org. Chem. Front.* **2021**, *8*, 3705-3711.
[5] P. Poutrel, M. V. Ivanova, X. Pannecoucke, P. Jubault, T. Poisson, *Chem. Eur. J.* **2019**, *25*, 15262-15266.
